# Brevilin A Ameliorates Acute Lung Injury and Inflammation Through Inhibition of NF-κB Signaling *via* Targeting IKKα/β

**DOI:** 10.3389/fphar.2022.911157

**Published:** 2022-06-14

**Authors:** Lu Liu, Xian Chen, Yifang Jiang, Yun Yuan, Luyao Yang, Qiongying Hu, Jianyuan Tang, Xianli Meng, Chunguang Xie, Xiaofei Shen

**Affiliations:** ^1^ TCM Regulating Metabolic Diseases Key Laboratory of Sichuan Province, Hospital of Chengdu University of Traditional Chinese Medicine, Chengdu University of Traditional Chinese Medicine, Chengdu, China; ^2^ College of Pharmacy, Chengdu University of Traditional Chinese Medicine, Chengdu, China; ^3^ Department of Pathology, Hospital of Chengdu University of Traditional Chinese Medicine, Chengdu, China; ^4^ Department of Laboratory Medicine, Hospital of Chengdu University of Traditional Chinese Medicine, Chengdu, China

**Keywords:** brevilin A, anti-inflammatory activity, acute lung injury, IKKα/β, NF-κB

## Abstract

Acute lung injury (ALI) is life-threatening disease characterized by uncontrolled inflammatory response. IKKα/β, the key kinases in the activation of NF-κB pathway, are implicated in inflammatory pulmonary injury, and represent attractive targets for ALI therapy. Brevilin A (BVA) is a sesquiterpene lactone from *Centipeda minima*, a Chinese herb used to treat inflammatory diseases. This study aims to investigate the inhibition of BVA on ALI, with focus on clarifying the molecular mechanisms involved in BVA-mediated anti-inflammatory activity in macrophages. Briefly, BVA significantly inhibited the production of NO and PGE_2_ by suppressing iNOS and COX2 expression, and suppressed the mRNA expression of IL-1β, IL-6, and TNFα in LPS/IFNγ-stimulated RAW264.7 macrophages. The anti-inflammatory activity of BVA was further confirmed in LPS/IFNγ-stimulated BMDMs and TNFα/IFNγ-exposed RAW264.7 cells. *In vivo*, BVA effectively attenuated LPS-induced lung damage, inflammatory infiltration, and production of pro-inflammatory cytokines, including MPO, IL-1β, IL-6, TNFα, and PGE_2_. Mechanistically, BVA could covalently bind to the cysteine 114 of IKKα/β, and effectively inhibiting the activity and function of IKKα/β, thereby resulting in the suppression of phosphorylation and degradation of IκBα and the subsequent activation of NF-κB signaling. Furthermore, pretreatment of DTT, a thiol ligand donor, significantly abolished BVA-mediated effects in LPS/IFNγ-stimulated RAW264.7 cells, suggesting the crucial role of the electrophilic α, β-unsaturated ketone of BVA on its anti-inflammatory activity. These results suggest that BVA ameliorates ALI through inhibition of NF-κB signaling via covalently targeting IKKα/β, raising the possibility that BVA could be effective in the treatment of ALI and other diseases harboring aberrant NF-κB signaling.

## Highlights

(1) Brevilin A inhibit LPS-induced acute lung injury
(2) Brevilin A covalently binds to IKKα/β, and inhibits IKKα/β-mediated NF-κB activation
(3) The α, β-unsaturated ketone of brevilin A is vital for its anti-inflammatory effect

## 1 Introduction

Normally, controllable inflammatory response is a protective immune response to eliminating endogenous and exogenous danger signals, including pathogens and tissues injury. Nevertheless, uncontrollable inflammation can cause severe tissue damage and organ dysfunction, which is harmful to the individual health, even is life-threatening (Chovatiya and Medzhitov, 2014; Horwitz et al., 2019). Acute lung injury (ALI), a typical uncontrollable inflammatory response, commonly results from sepsis, acute pancreatitis, and ischemia-reperfusion. Pathologically, ALI is characterized by increased permeability of pulmonary capillary, inflammatory cell infiltration, and diffuse pulmonary oedema, ultimately resulting in respiratory failure (Butt et al., 2016; Hughes and Beasley, 2017). Furthermore, acute respiratory distress syndrome, the most severe form of ALI, has been considered as the leading cause of death in coronavirus disease-19-induced pneumonia (Li et al., 2020). Currently, several therapeutic measures including mechanical ventilation and glucocorticoid has been used in ALI therapy, whereas the clinical benefits of these treatments are limited (Mokra et al., 2019). Therefore, it is of urgent need to develop novel therapies with higher safety and effectiveness for the treatment of ALI.

Fundamental to ALI is the acute onset of pulmonary inflammation, which is intimately related to the activation of alveolar macrophages (Laskin et al., 2019). Alveolar macrophages, the most abundant resident phagocytes in the lung interstitium and alveoli, represent the first line of defense against incoming pathogens and pollutants (Joshi et al., 2018). Lipopolysaccharide (LPS), an infectious stimulus from Gram-negative bacteria, is considered to be one of the main causes of ALI. Alveolar macrophages can efficiently recognize LPS by toll-like receptor 4 (TLR4). Upon binding to TLR4, LPS triggers a series of signaling cascades, including the activation of nuclear factor kappa-B (NF-κB) pathway and mitogen-activated protein kinases (MAPKs), thereby leading to the initiation, amplification, and maintenance of inflammation by producing pro-inflammatory cytokine (including interleukin 1β [IL-1β], IL-6, and tumor necrosis factor-α [TNFα]) and mediators (such as nitric oxide [NO] and prostaglandin-E2 [PGE_2_]) (Butt et al., 2016; Chen et al., 2020). Therefore, inhibition of the hyperactivation of alveolar macrophages by targeting the pro-inflammatory signaling may potentially be an effective strategy for the treatment of ALI (Arora et al., 2018; Mehta et al., 2020).

**FIGURE 1:**
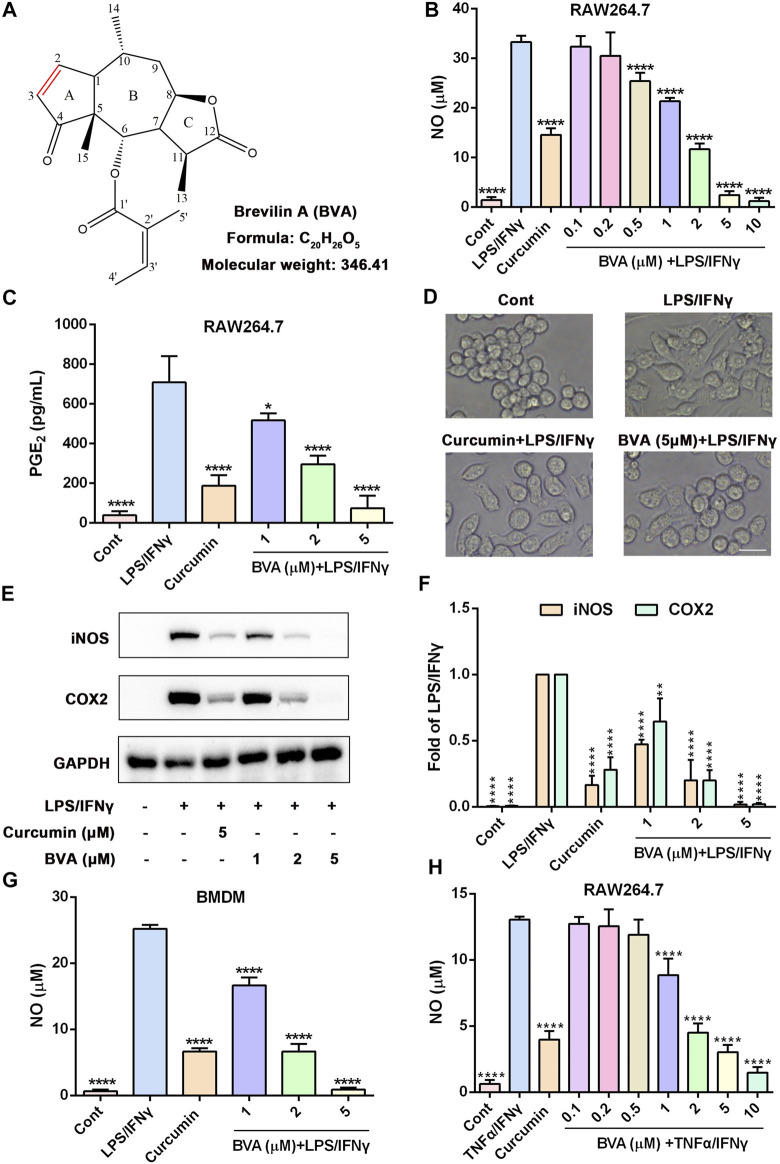
Brevilin A inhibits the inflammatory response in vitro. **(A)** The chemical structure of brevilin A (BVA). RAW264.7 cells were treated with BVA or curcumin (5 μM) for 1 h, and then stimulated with LPS/IFNγ (0.5 and 10 ng/ml) for 24 h. The production of NO **(B)** and PGE2 **(C)** were detected by Griess reagent or ELISA kit, respectively. **(D)** The representative morphological changes of RAW264.7 cells are shown. Scale bar = 25 μm. **(E)** The whole-cell lysates were immunoblotted with anti-iNOS and anti-COX2 antibodies, respectively. GAPDH was shown as the loading control. **(F)** The quantitative results of the ratio of iNOS or COX2 normalized to GAPDH are shown. BMDMs **(G)** or RAW264.7 **(H)** were treated with BVA or curcumin for 1 h, and following incubated with LPS/IFNγ or TNFα/IFNγ (10 and 10 ng/ml) for additional 24 h. The NO release was measured by Griess reagent. *p < 0.05, **p < 0.01, ***p < 0.001, ****p < 0.0001, vs. LPS/IFNγ or TNFα/IFNγ.

Increasing evidence suggests that natural products exhibit a great potential and prospect in ALI therapy (He et al., 2021). Brevilin A (BVA, Figure 1A) is a sesquiterpene lactone isolated from *Centipeda minima*, a Chinese herb that is frequently used to treat a variety of respiratory diseases, including cough and asthma (Li et al., 2020). Brevilin A has been proved to possess multiple pharmacological effects, including anti-inflammatory (Zhou et al., 2020; Qin et al., 2021), anti-tumor (Chen et al., 2013; Khan et al., 2020), anti-viral (Zhang et al., 2019), and anti-oxidative (Zhou et al., 2020) activities. Although previous studies have shown that brevilin A can inhibit neuro-inflammation and inflammasome activation (Zhou et al., 2020; Qin et al., 2021), the protective effect and the underlying mechanisms of brevilin A on ALI, as well as its protein targets is still unknown. Herein, we found that brevilin A effectively inhibits LPS/interferon-γ (IFNγ)- or TNFα/IFNγ-induced inflammatory response in RAW264.7 macrophages and bone-marrow derived macrophages (BMDMs) *in vitro*, as well as LPS-induced ALI *in vivo*. Furthermore, we identified that inhibitor of nuclear factor kappa B kinase α/β (IKKα/β) are the functional targets of brevilin A in macrophages. Mechanistically, brevilin A may covalently modify the cysteine 114 (Cys114) of IKKα/β, thereby inhibiting the activation and function of IKKα/β, ultimately blocking the activation of NF-κB pathway.

## 2 Materials and Methods

### 2.1 Chemical and Reagents

Brevilin A (BVA) was provided by PUSH Bio-Technology (Chengdu, China, Figure 1A, the purity ≥98.0%). LPS (*E. coli* 0111:B4) was purchased from Sigma (Shanghai, China). Murine IFNγ and TNFα were obtained from SinoBiological (Beijing, China). Curcumin, BAY 11-7082, MG132, and LY294002 were purchased from Beyotime (Shanghai, China). PD98059, SB203580, and SP600125 were provided by Selleck (Shanghai, China). Primary antibodies against inducible nitric oxide synthase (iNOS), cyclooxygenase 2 (COX2), poly [ADP-ribose] polymerase 1 (PARP1), GAPDH, and Tubulin were obtained from ProteinTech (Wuhan, China). The antibodies against IKKα/β, phosphorylated-IKKα/β, IκBα, phosphorylated-IκBα, NF-κB p65, and Flag were purchased from Beyotime (Shanghai, China). Extracellular regulated protein kinases (ERK), phosphorylated-ERK, c-Jun N-terminal kinases (JNK), phosphorylated-JNK, p38, phosphorylated-p38, RAC-alpha serine/threonine-protein kinases (Akt), and phosphorylated-Akt were provided by Signalway Antibody (Baltimore, United States).

### 2.2 Cell Culture

Murine RAW264.7 macrophages and HEK293T cells were obtained from Beyotime (Shanghai, China), and were cultured in Dulbecco’s Modified Eagle Medium (DMEM) high glucose medium (Gibco) supplemented with 10% fetal bovine serum (FBS, PAN Biotech, Germany) and 1% penicillin-streptomycin (Gibco) at 37°C in a humidified atmosphere of 5% CO_2_.

Murine BMDMs were obtained as described previously (Qin et al., 2021). Briefly, bone marrow cells were collected from the tibia and femurs of 8-weeks-old C57BL/6 mice by flushing. After being removed the red blood cells, cells were cultured in DMEM high glucose medium supplemented with 10% FBS and 1% penicillin-streptomycin, and then differentiated with 25 ng/ml mouse macrophage colony stimulating factor (M-CSF, Novoprotein, Shanghai, China) for 6 days.

### 2.3 Detection of Inflammatory Mediators

RAW264.7 cells or BMDMs were seeded into 96-well plates, and incubated at 37°C overnight. Cells were then treated with BVA or curcumin (positive control) for 1 h, following stimulated with LPS/IFNγ (0.5 and 10 ng/ml) or TNFα/IFNγ (10 and 10 ng/ml) for additional 24 h. Next, the cell culture mediums were collected to detect the levels of NO and PGE_2_ by Griess reagent (Beyotime) and enzyme-linked immunosorbent assay (ELISA) kits (R&D Systems, Minneapolis, MN, United States), respectively.

### 2.4 Real Time Quantitative Polymerase Chain Reaction (RT-qPCR)

Total RNAs were extracted from RAW264.7 cells or lung tissues of mice by using UNlQ-10 Column total RNA Purification Kit (Sangon Biotech, Shanghai, China). The total RNAs were then reverse-transcribed to cDNAs by using SureScript™ First-Strand cDNA Synthesis Kit (GeneCopoeia, Guangzhou, China). Next, RT-qPCR was performed on a 7500 Real Time PCR System (Applied Biosystems, United States) according to the instructions of BlazeTaq™ SYBR Green qPCR Mix 2.0 (GeneCopoeia, Guangzhou, China). The PCR primers were synthetized by Tsingke Biotech (Chengdu, China), and the primer sequences are shown in Supplementary Table S1. Relative expression levels of target genes were calculated based on 2^−ΔΔCt^ method. GAPDH was used as the internal control for normalization.

### 2.5 Western Blotting Assay

Briefly, RAW264.7 cells were treated with BVA and LPS/IFNγ or TNFα/IFNγ for the indicated times. Cells were then lysed with RIPA buffer containing 1% protease and phosphatase inhibitors (ApexBio, United States) for 15 min on ice, and total protein was collected. After being boiled with loading buffer, protein samples were separated using sodium dodecyl sulfate-polyacrylamide gel electrophoresis (SDS-PAGE), and then transferred into a polyvinylidene fluoride (PVDF) membrane. Membranes were blocked with 5% non-fat milk powder dissolved in PBS for 1 h at room temperature (RT). Afterwards, the membranes that immobilized target proteins were incubated specific primary antibodies (1:1000) at 4°C overnight. After being washed with tris-buffered saline containing 0.05% Tween 20 (TBST) buffer for 3 times, the membranes were incubated with horseradish peroxidase labeled-IgG (1:5000, ProteinTech, Wuhan, China) for 1 h at RT. Finally, the signals were detected by chemiluminescence reagent (4A Biotech, Beijing, China). The signals of each band were quantified by imageJ software.

### 2.6 LPS-Induced ALI Mice Model

Male ICR and C57BL/6 mice (SPF grade, 18–22 g, 6–8 weeks old) were obtained from Dashuo Biological Technology (Chengdu, China). Mice were kept in the plastic cages filled with poplar wood shavings on a 12-h light/dark cycle at 25 ± 1°C, and free to access clean food and water. All experiment procedures were approved by the Institutional Animal Care and Use Committee of Chengdu University of Traditional Chinese Medicine (2021-09).

After 1 week of adaptive feeding, mice were randomly divided into four groups as follows: Control group, LPS group, LPS+dexamethasone (DEX, 5 mg/kg) group, and LPS+BVA (20 mg/kg) group. Each group contained 8 mice. To induce ALI, mice were anesthetized by 10% chloral hydrate, and instilled intratracheally with LPS (25 μg/mice). The control mice were instilled intratracheally with equal volume of normal saline. Subsequently, mice were intraperitoneally injected with BVA, DEX, or vehicle (10% DMSO, 30% PEG400, and 60% normal saline) at 2 h before LPS exposure, as well as 6 and 18 h after LPS challenge.

### 2.7 The Cell Counting of Bronchoalveolar Lavage Fluid (BALF)

Two hours after the last administration of drugs, mice were anesthetized by 10% chloral hydrate. Next, the BALF was collected using the slow infusion and extraction of 1 ml of pre-cold normal saline for 2 times *via* endotracheal intubation. Firstly, the protein contents of BALF were measured by bicinchoninic acid (BCA) assay kit (Beyotime). Subsequently, the BALF was centrifuged at 1,000 g for 3 min at 4°C. The precipitated cells were collected and resuspended with 1 ml of normal saline. The numbers of cells in the BALF were counted by the cell counting plates under a light microscopy. The precipitated cells were then coated on the glass slides, and stained by Diff-Quick stain (Solarbio, Beijing, China). Finally, the numbers of neutrophils, macrophages, and epithelial cells were counted using a light microscopy.

### 2.8 Detection of Cytokines in Serum as Well as Examination of Myeloperoxidase Activity and Histopathology in Lung Tissues

After being anesthetized, the blood samples were collected via mice orbit. The blood samples were centrifuged at 1,000 g for 10 min, and the serums were collected to detect the release of IL-1β, IL-6, TNFα, and PGE_2_ using ELISA kits (Valukine, R&D, Minneapolis, United States) according to the manufacturer’s instructions. Next, mice were sacrificed by cervical dislocation, and lung tissues were isolated and divided into two parts. On the one hand, a portion of lung tissues were washed with cold-PBS, and homogenized with cold-PBS (1:9, w/v) supplemented with 1% protease and phosphatase inhibitors on ice. The MPO activity in lung tissues was measured by MPO assay kit (Nanjing Jiancheng Bioengineering Institute, Nanjing, China). Furthermore, the protein levels of IκBα and phosphorylated IKKα/β in lung tissues were detected by western blotting. On the other hand, lung tissues were fixed in 4% paraformaldehyde overnight. The lung tissues were then dehydrated, embedded in paraffin, and cut into sections. After dewaxing, the sections were stained with hematoxylin and eosin (HE). The histopathologic changes of lung tissues were scored as described previously (Matute-Bello et al., 2011).

### 2.9 Cytoplasmic and Nuclear Fractionation

After being treated with BVA and LPS/IFNγ for the indicated times, RAW264.7 cells were washed with PBS and collected by centrifuging (1,000 g) for 3 min at 4°C. Next, the cytoplasmic and nuclear proteins were extracted using a Nuclear and Cytoplasmic Protein Extraction Kit (Beyotime) according to the manufacturer’s instructions. The cytoplasmic and nuclear distribution of NF-κB p65 was detected using western blotting.

### 2.10 Immunofluorescence Assay

RAW264.7 cells (1 × 10^5^) adherent on the glass bottom dishes were pretreated with brevilin A (5 μM) or DMSO for 2 h, and following stimulated with or without LPS/IFNγ for 30 min. The cells were fixed with 4% paraformaldehyde for 15 min, permeabilized with 0.1% Triton X-100 for 10 min, and incubated with anti-p65 antibody (1:200) overnight at 4°C. Following incubation with Alexa Fluor 555-labeled secondary antibody (1:1000, Beyotime) and 4',6-diamidino-2-phenylindole (DAPI, 2.5 μg/ml), the cells were evaluated for the cellular localization of p65 using a confocal microscope (TCS SP8; Leica, Germany).

### 2.11 Target Validation

For cellular thermal shift assay (CETSA), RAW264.7 cells (1 × 10^7^) were collected, resuspend with PBS containing 1% protease inhibitor, and freeze-thawed three to 3 times using liquid nitrogen. Then, cell lysates were centrifuged at 12,000 g for 10 min at 4°C. Next, the supernatant was collected, divided into 2 aliquots, and treated with BVA (50 μM) or DMSO for 30 min at 37°C. Subsequently, BVA- and DMSO-treated cell lysates were respectively divided into 7 equal samples, and respectively heated at 45°C, 48°C, 51°C, 54°C, 57°C, 60°C, and 63°C for 3 min. After centrifugation at 12,000 g for 10 min at 4°C, soluble fractions were collected. The thermal stability of target proteins was evaluated by western blotting.

For drug affinity responsive target stability (DARTS) assay, RAW264.7 cells (1 × 10^7^) were lysed with 600 μl could M-PER buffer (Thermo Fisher Scientific) supplemented with 1% protease/phosphatase inhibitors. After centrifugation at 12,000 g for 15 min, the supernatant was collected, and mixed with 10 × TNC buffer (50 mM Tris-HCl, pH = 8.0, 50 mM NaCl, 10 mM CaCl_2_). The lysates were equally divided equally into two samples, and incubated with BVA (50 μM) or DMSO for 1 h at 25°C. Next, samples were then incubated with pronase (1:2,000, Roche) for 30 min at 25°C. The samples were boiled with loading buffer, and analyzed by SDS-PAGE followed by western blotting.

### 2.12 Liquid Chromatography-Mass Spectrum (LC-MS) Analyses

For determination of BVA-binding site on IKKβ, recombinant human IKKβ protein (ProteinTech, Wuhan, China) was incubated with BVA with a molar ratio of 1:10 for 8 h at 4°C, and the mixture was separated by SDS-PAGE, and stained by Coomassie brilliant blue. The protein band of interest was excised, destained, and reduced, followed by in-gel trypsin digestion. The peptides were extracted and analyzed by the LC-MS/MS experiments were performed using a Q Exactive HF-X mass spectrometer (Thermo Scientific). Mass spectrometric data were analyzed with Proteome Discoverer 2.4 software.

For detection of adduct, BVA (10 μM) was incubated with dithiothreitol (DTT, 50 μM) in PBS for 30 min at 37°C. The BVA-DTT adduct was then analyzed by the LC-MS system (Waters Vion^®^ IMS QT, Milford, United States). The mobile phase consisting of water and methanol (1:1) was delivered at a constant flow rate of 0.2 ml/min.

### 2.13 Molecular Docking

Molecular docking assay was performed with Autodock 4.2. The crystal structure of human IKKβ (PDB ID: 3RZF) was downloaded from the Protein Data Bank (PDB), and prepared by the Protein Preparation Workflow, including adding polar hydrogen and removing water and ligand. Then, global energy was minimized using the OPLS3e force field. The Cys114 was used to define the central site of the grid box using Glide. Covalent docking was performed using a Michael addition reaction. The optimal docking pose was visualized using Pymol.

### 2.14 *In Vitro* Kinase Assay

In brief, HEK293T cells were transfected with human Flag-IKKβ plasmid (GeneChem, Shanghai, China) for 24 h, and lysed with immune-precipitation buffer supplemented with 1% protease inhibitor on ice for 15 min. Next, the Flag-IKKβ protein was immunoprecipitated from the cell lysates by anti-Flag affinity gel (Beyotime), and then competitively eluted from gel using 3×Flag peptide (Beyotime). Subsequently, Flag-IKKβ (2 μg/ml) was treated with BVA, BAY 11-7082 or DMSO for 30 min at 37°C, and then incubated with human recombinant IκBα (2 μg/ml, ProspecBio, Rehovot, Israel) in kinase buffer (CST, United States) containing 5 mM ATP for 1 h at 25°C. The phosphorylation of IκBα was detected by western blotting.

### 2.15 Statistical Analysis

Data are expressed as mean values ± standard deviation (SD), and analyzed by one-way analysis of variance (ANOVA) followed by Dunnett’s multiple comparisons using GraphPad Prism 5.0 software. Differences were considered significant when *p* < 0.05.

## 3 Results

### 3.1 Brevilin A Inhibits the Inflammatory Response *In Vitro*

Brevilin A (Figure 1A) is a sesquiterpene lactone that isolated from *C. minima*, a medicinal plant that has been used in traditional Chinese medicine for the treatment of various inflammatory diseases, including rhinitis and cough (Li et al., 2020). Thus, we here attempted to examine the anti-inflammatory activity of brevilin A *in vitro*. As shown in Figure 1B, brevilin A dose-dependently inhibited NO generation in LPS/IFNγ-stimulated murine RAW264.7 macrophages with a half inhibitory concentration (IC_50_) value of 1.35 μM, but without obvious cytotoxicity (Supplementary Figure S1). Furthermore, brevilin A significantly decreased LPS/IFNγ-induced production of PGE_2_ in RAW264.7 cells (Figure 1C). In addition, we also observed an obvious morphological change in LPS/IFNγ-exposed RAW264.7 cells, including pseudopodia formation, which could be suppressed by brevilin A (Figure 1D).

Several pro-inflammatory enzymes, such as iNOS and COX2 are key enzymes in the biosynthesis of NO and PGE_2_ respectively, play an important role in inflammatory response (Mehta 2005; Fukunaga et al., 2005). Thus, we investigated whether the inhibition of brevilin A on the production of NO and PGE_2_ was owing to its downregulation of the expression of iNOS and COX2. As shown in Supplementary Figures S2A,B, as well as Figures 1E,F, brevilin A effectively diminished the mRNA and protein expression of iNOS and COX2 in a dose-dependent manner in LPS/IFNγ-activated RAW264.7 cells, respectively. Moreover, brevilin A treatment also markedly inhibited the mRNA expression of pro-inflammatory cytokines, including IL-1β (Supplementary Figure S2C), IL-6 (Supplementary Figure S2D), and TNFα (Supplementary Figure S2E) in LPS/IFNγ-stimulated RAW264.7 cells. In addition to RAW264.7 macrophage cells, we also found that brevilin A markedly decreased LPS/IFNγ-induced NO production in primary BMDMs (Figure 1G). Being similar to LPS, TNFα also can trigger a potent inflammatory response (Malaviya et al., 2017). Therefore, we further studied whether brevilin A inhibits inflammatory response in TNFα/IFNγ-stimulated RAW264.7 cells. As is shown in Figure 1H, TNFα/IFNγ-induced NO generation could be significantly decreased by brevilin A in a dose-dependent manner in RAW264.7 cells. Taken together, these results suggest that brevilin A exhibits a potent anti-inflammatory effect *in vitro*.

### 3.2 Brevilin A Alleviates Lipopolysaccharide-Induced Acute Lung Injury *In Vivo*

**FIGURE 2:**
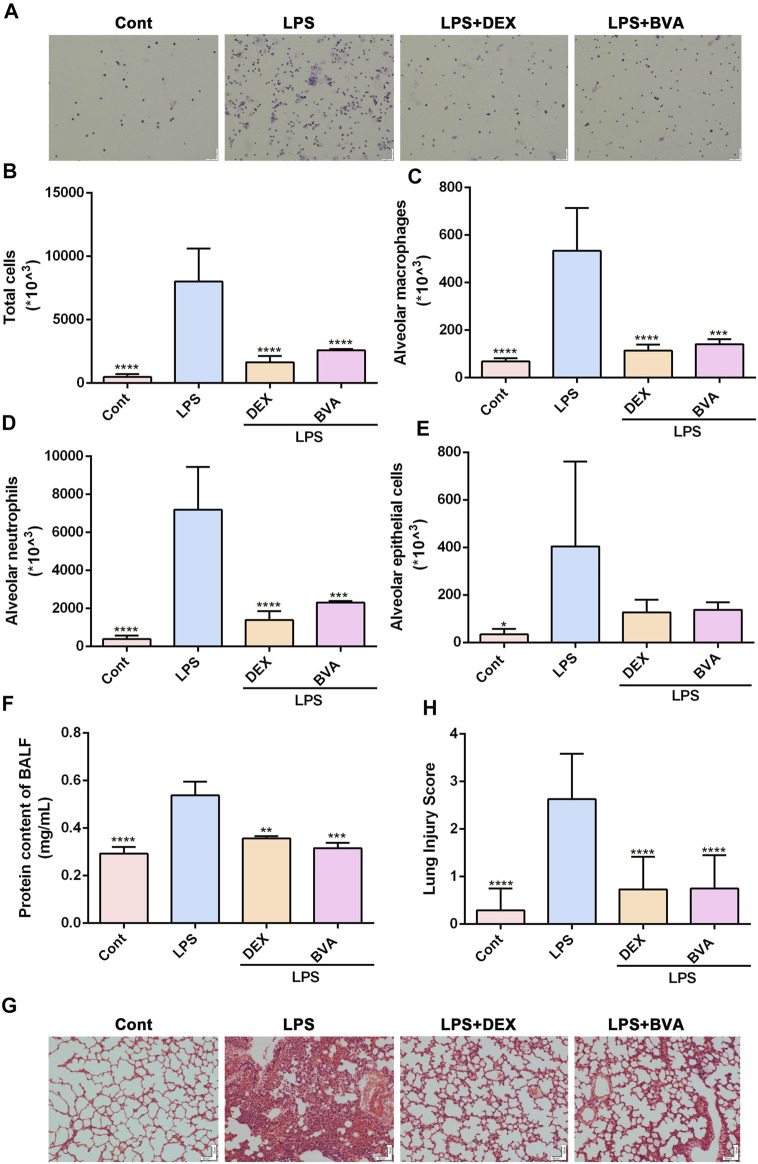
Brevilin A attenuates LPS-induced ALI in vivo. After being exposed to LPS, mice were treated with BVA (20 mg/kg), DEX (5 mg/kg), or vehicle. **(A)** The representative Diff-Quick staining images of BALF cells were shown. The numbers of total cells **(B)**, alveolar macrophage **(C)**, neutrophils **(D)**, and epithelial cells **(E)** in BALF were counted. **(F)** The protein contents in BALF were measured using BCA method. The HE staining was used to assess the degree of inflammatory lung injury. **(G)** The representative histopathological images of lung tissues are shown. Scale bar = 50 μm. **(H)** The quantitative results of pathological scores are presented. DEX, dexamethasone. *p < 0.05, **p < 0.01, ***p < 0.001, ****p < 0.0001, vs. LPS.

To further investigate the anti-inflammatory effect of brevilin A *in vivo*, we established an ALI mice model by intratracheal instillation of LPS. Firstly, we found that intratracheal instillation of LPS caused a significant elevation of total cells in BALF, which could be greatly attenuated by the treatment of brevilin A (Figures 2A,B). Furthermore, Diff-Quik Stain showed that the numbers of alveolar macrophages, neutrophils, and epithelial cells in BALF were highly elevated in LPS-challenged mice, while intraperitoneal administration of brevilin A or dexamethasone was shown to cause a significant suppression in the abnormal increase of these three cells in BALF (Figures 2A,C–E). Additionally, BCA assay showed that LPS challenge resulted in a marked increase in protein content of BALF, as compared with the normal control. Treatment of brevilin A effectively inhibited LPS-induced increased content of protein in BALF (Figure 2F). Finally, the substantial pathological alterations including edema, hyperemia, alveolar collapse, and inflammatory cell infiltration were observed in LPS-exposed mice via histological examination, whereas treatment with brevilin A markedly ameliorated these pathological changes (Figure 2G), and decreased the pathological scores (Figure 2H), including neutrophil in the alveolar space, neutrophil in the interstitial space, hyaline membranes, protrinaceous debris filling airspaces, and alveolar septal thickening. These data suggested that brevilin A can effectively alleviate LPS-induced ALI in mice.

### 3.3 Brevilin A Suppresses the Activity of MPO and Expression of Pro-Inflammatory Cytokines in LPS-Exposed Mice

**FIGURE 3:**
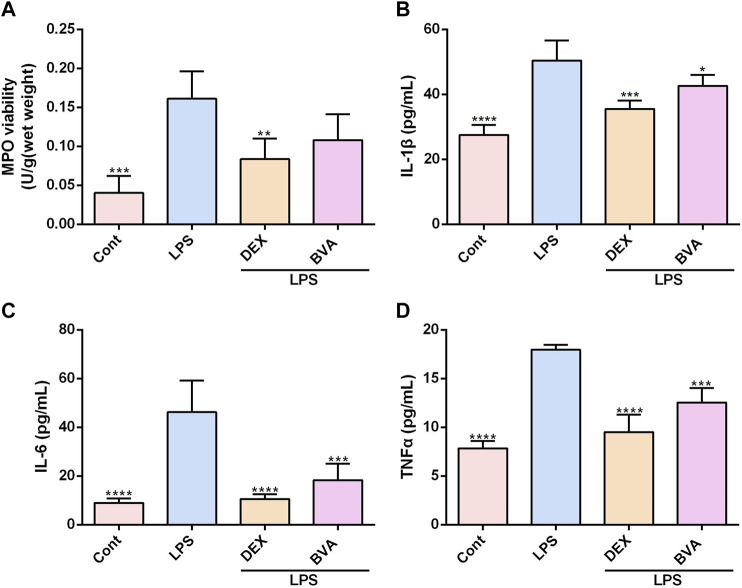
Brevilin A inhibits MPO activity and synthesis of cytokines in LPS-challenged mice. After LPS expose, mice were treated with BVA, DEX, or vehicle. The lung tissues were used to detect the activity of MPO **(A)**. The serums were collected to examine the levels of IL-1β **(B)**, IL-6 **(C)**, and TNFα **(D)** using ELISA kits. *p < 0.05, **p < 0.01, ***p < 0.001, ****p < 0.0001, vs. LPS.

To further verify the anti-inflammatory effect of brevilin *in vivo*, we assessed the levels of several pro-inflammatory factors in the lung tissues or serum in LPS-challenged mice. As shown in Figure 3A, the activity of MPO, a well-known biomarker of neutrophil infiltration and ALI (Meyer et al., 2017), was significantly increased in the lung tissues in the mice exposed to LPS. The LPS-induced up-regulation of MPO activity tended to be decreased in brevilin A-treated mice, as compared with the vehicle-treated mice, although the difference was statistically insignificant. Overproduction of pro-inflammatory cytokines and mediators is the main feature of several inflammatory diseases, including ALI (Goodman et al., 2003). In this study, the increased serum levels of various pro-inflammatory cytokines including IL-1β, IL-6, and TNFα were also observed in LPS-stimulated mice, whereas brevilin A greatly repressed the release of these cytokines (Figures 3B–D). Furthermore, LPS-induced elevation of COX2 mRNA in lung tissues (Supplementary Figure S3A) and PGE_2_ release in serums (Supplementary Figure S3B) were markedly suppressed by brevilin A treatment. In addition, LPS expose caused a decreased mRNA expression of IL-10, an anti-inflammatory cytokine in lung tissues of mice. However, brevilin A treatment could not significantly enhance the mRNA expression of IL-10 (Supplementary Figure S3C) in LPS-challenged mice. As such, it can be concluded that brevilin A exhibits a potent therapeutic effect in LPS-induced ALI, and has great potential as an anti-inflammatory agent.

### 3.4 Brevilin A Acts as a Potent Inhibitor of NF-κB Pathway *In Vitro* and *In Vivo*

**FIGURE 4:**
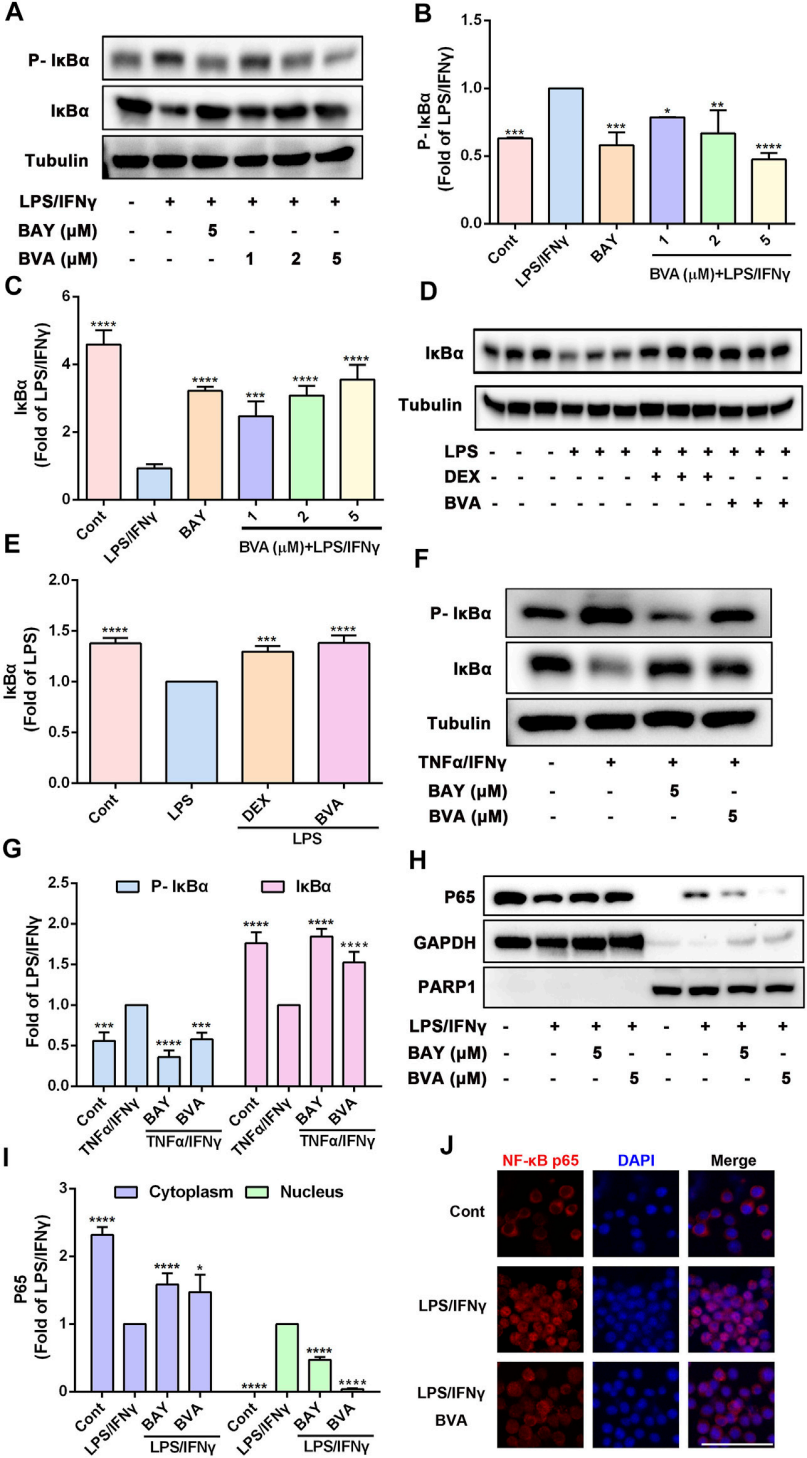
Brevilin A suppresses the activation of NF-κB pathway in vitro and in vivo. RAW264.7 cells treated with BVA (1, 2, and 5 μM) or BAY (BAY11-7082, 5 μM) for 2 h and then stimulated with LPS/IFNγ (0.5 and 10 ng/ml) for 5 min or TNFα/IFNγ (10 and 10 ng/ml) for 10 min, respectively. Whole-cell lysates from LPS/IFNγ-activated cells **(A)** or TNFα/IFNγ-stimulated cells **(F)** were used to detect the phosphorylation and degradation levels of IκBα by western blotting, respectively. The quantification of the ratio of phosphorylated IκBα (**B,G**) or total IκBα (**C,G**) normalized to tubulin are shown. **(D,E)** Protein levels of IκBα in lung tissues from BVA-, DEX-, or vehicle-treated mice that had been stimulated with LPS were determined by immunoblotting and normalized using Tubulin. **(H)** RAW264.7 cells were treated with BVA or BAY for 2 h, followed by stimulating with LPS/IFNγ for 30 min. The cytoplasmic and nuclear fractions were then subjected to detect the distribution of NF-κB p65 *via* immunoblotting. GAPDH and PARP1 were used as loading controls, respectively. **(I)** Statistical results of the ratio of p65 normalized to GAPDH or PARP1 are presented. **(J)** Immunofluorescent assessment of nuclear translocation of NF-κB p65 (red) in RAW264.7 cells. Cells were counterstained with DAPI (blue). Scale bar = 25 μm *p < 0.05, **p < 0.01, ***p < 0.001, ****p < 0.0001, vs. LPS, LPS/IFNγ or TNFα/IFNγ.

The potent anti-inflammatory activity of brevilin A *in vitro* and *in vivo* prompted us to investigate its underlying molecular mechanisms. Because NF-κB pathway is responsible for the trigger, amplification, and maintenance of inflammation, we thus further tested whether brevilin A could regulate the activation of NF-κB signaling. Phosphorylation of IκB is a key step in the activation of NF-κB signaling cascade (Liu et al., 2017). As expected, LPS/IFNγ markedly enhanced the phosphorylation of IκBα in RAW264.7 cells, which was effectively blocked by brevilin A treatment in a dose-dependent fashion (Figures 4A,B), which also as does BAY11-7082, a well-identified IκB inhibitor. Upon phosphorylation, IκBα can be degraded via a proteasome-dependent pathway, which contributes to liberate free NF-κB dimers as well as subsequent activation (Liu et al., 2017). As shown in Figures 4A,C, brevilin A significantly suppressed LPS/IFNγ-induced degradation of IκBα in RAW264.7 cells in a dose-dependent manner, as compared with the vehicle control. Furthermore, in the presence of MG-132, a potent proteosome inhibitor that inhibits proteasome-mediated degradation of IκBα, brevilin A still blocked LPS/IFNγ-induced IκBα phosphorylation in RAW264.7 cells (Supplementary Figure S4). We also investigated the influence of brevilin A on the degradation of IκBα in lung tissues obtained from LPS-challenged mice. The degradation of IκBα was remarkedly inhibited in the mice that had been treated with brevilin A, but not in the vehicle-treated mice (Figures 4D,E), which was consistent with the results obtained from the *in vitro* experiments.

Because TNFα is a potent activator of NF-κB pathway, and brevilin A also could inhibit the NO release in TNFα/IFNγ-stimulated RAW264.7 cells, we therefore detected whether brevilin A blocks TNFα/IFNγ-induced phosphorylation and degradation of IκBα. As shown in Figures 4F,G, brevilin A not only inhibited the phosphorylation of IκBα, but also inhibited the degradation of IκBα in TNFα/IFNγ-treated RAW264.7 cells. As IκBα is degraded, free NF-κB can be able to translocate from the cytoplasm to the nucleus, where it can induce the expression of inflammatory genes (Liu et al., 2017). Therefore, we then evaluated the effect of brevilin A on the nuclear translocation of NF-κB p65. Immunoblotting assay showed that brevilin A effectively abolished LPS/IFNγ-induced the reduction of NF-κB p65 in cytoplasmic fractionations and increase of NF-κB p65 in nuclear fractionations, suggesting that brevilin A inhibited the nuclear translocation of NF-κB p65 (Figures 4H,I). Similarly, *in situ* detection of fluorescent NF-κB p65 further corroborated that brevilin A blocked LPS/IFNγ-induced nuclear translocation of NF-κB in RAW264.7 cells (Figure 4J). Collectively, these findings demonstrate that brevilin A acts as a potent inhibitor of NF-κB signaling induced by diverse pro-inflammatory irritants *in vitro* and *in vivo*.

### 3.5 Brevilin A Does Not Exhibit Obvious Inhibition on MAPKs and PI3K/Akt Pathways

MAPKs, a family of serine/threonine protein kinases contained JNK, ERK, and p38, regulate a variety of biological processes and cellular responses to external stimuli. Hyperactivation of MAPKs is implicated in various inflammatory and autoimmune diseases via promoting the induction of pro-inflammatory cytokines and mediators at transcriptional and translational levels (Arthur and Ley, 2013). Although LPS/IFNγ incubation significantly enhanced the phosphorylation levels of JNK (Supplementary Figures S5A,B), ERK (Supplementary Figures S5C,D), and p38 (Supplementary Figures S5E,F), brevilin A treatment at dose of 1, 2, and 5 μM could not repress the activation of these MAPKs. Except for MAPKs, PI3K/Akt signaling is also involved in the regulation of immunity *via* orchestrating the response to different metabolic and pro-inflammatory signals in macrophages (Vergadi et al., 2017). Hence, intervention in PI3K/Akt pathway has been considered as an effective strategy for anti-inflammatory therapeutics. Unexpectedly, the inhibition of brevilin A on LPS/IFNγ-induced activation of Akt was not observed at concentrations of 1–5 μM in RAW264.7 cells (Supplementary Figures S5G,H). Conversely, JNK inhibitor SP600125, ERK inhibitor PD98059, p38 inhibitor SB203580, and PI3K inhibitor LY294002 could down-regulate the phosphorylation of MAPKs and Akt in LPS/IFNγ-stimulated RAW264.7 cells, respectively. These above data strongly suggested that brevilin A at concentrations of 1–5 μM tends to inhibit the activation of NF-κB signaling pathway rather than the MAPKs and PI3K/AKt pathways.

### 3.6 The α, β-Unsaturated Ketone Moiety of Brevilin A is Crucial for Its Anti-Inflammatory Activity

**FIGURE 5:**
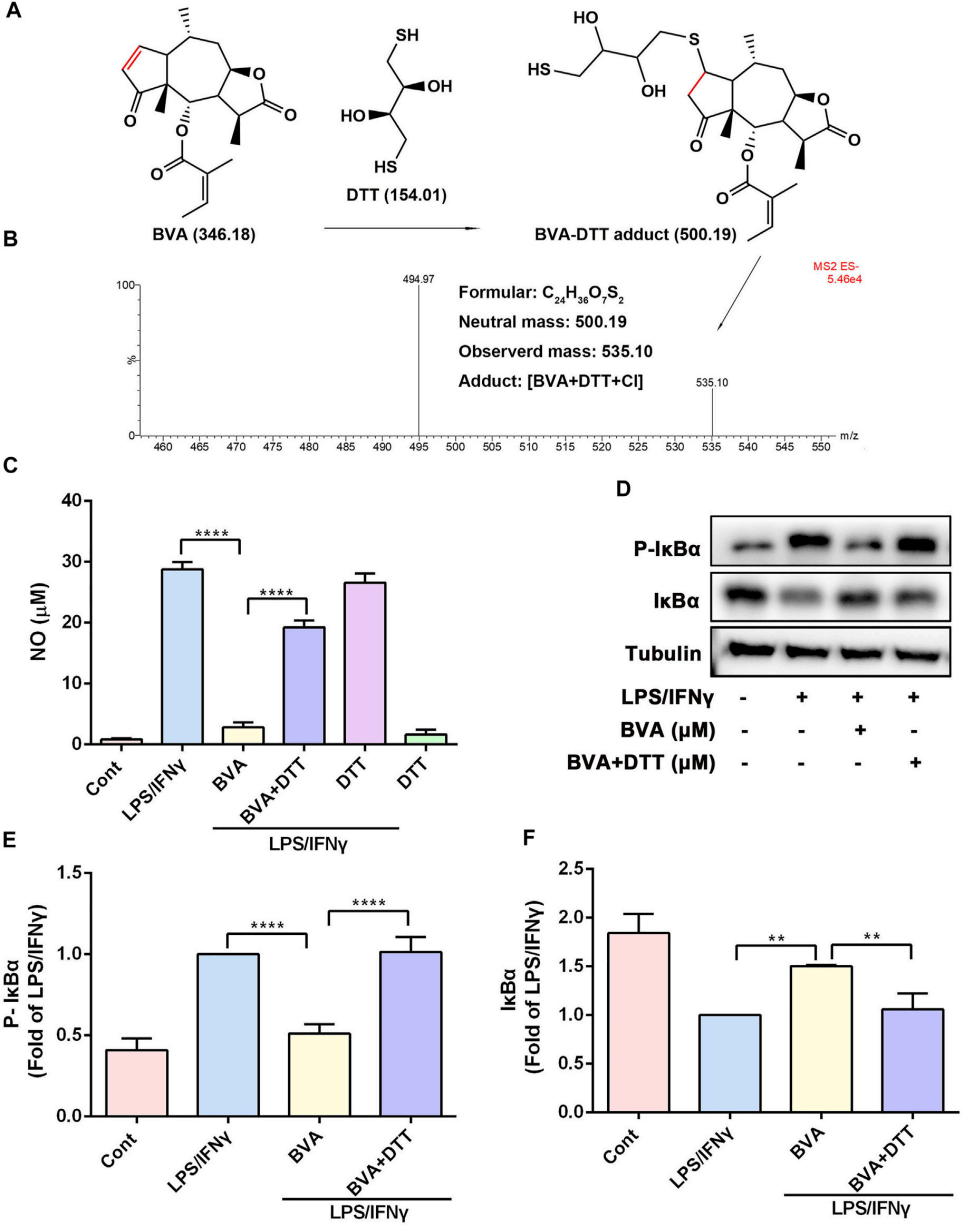
The α, β-unsaturated ketone moiety of brevilin A is crucial for its anti-inflammatory activity in vitro. **(A)** Michael addition reaction between BVA and DTT. **(B)** The adduct product of BVA and DTT was analyzed by LC-MS. Molecular weights of the molecules are indicated. **(C)** The comparison of DTT-treated and vehicle-treated BVA on NO production in LPS/IFNγ-stimulated RAW264.7 cells. **(D)** RAW264.7 cells were treated with BVA or DTT-incubated BVA for 2 h, and then exposed to LPS/IFNγ for 5 min. Then, the levels of p-IκBα and IκBα, as well as Tubulin were detected by immunoblotting. Densitometry analysis of p-IκBα **(E)** and IκBα **(F)** normalized against Tubulin, respectively. *p < 0.05, **p < 0.01, ***p < 0.001, ****p < 0.0001, vs. BVA.

Several studies have been suggested that the α, β-unsaturated ketone is crucial for the anti-inflammatory activity of a series of natural products (Liao et al., 2017; Liu et al., 2019). We then assessed the contribution of the α, β-unsaturated carbonyl group of brevilin A to its inhibitory activity *in vitro*. As shown in Figure 5A, DTT, a reducing agent with a nucleophilic thiol, might be covalently modified by the electrophilic C2=C3 double bond of brevilin A. The LC-MS result showed that a new addition product at m/z 535.10 [BVA+DTT+Cl] was detected (Figure 5B), and indicating the covalent binding of brevilin A to the thiol *in vitro*. As shown in Figure 5C, pre-incubation with DTT significantly abolished brevilin A-mediated repression on LPS/IFNγ-induced NO production in RAW264.7 cells. Furthermore, the immunoblotting assay indicated that DTT-incubated brevilin A failed to inhibit LPS/IFNγ-induced phosphorylation (Figures 5D,E) and degradation (Figures 5D,F) of IκBα in RAW264.7 cells. These results suggest that brevilin A almost completely lost its anti-inflammatory activity when its unsaturated double bond was reduced by DTT. Therefore, the α, β-unsaturated ketone moiety is the most important contributor to the inhibitory effect of brevilin A.

### 3.7 Brevilin A Directly Interacts With IKKα/β, Thereby Inhibiting Their Activation

**FIGURE 6:**
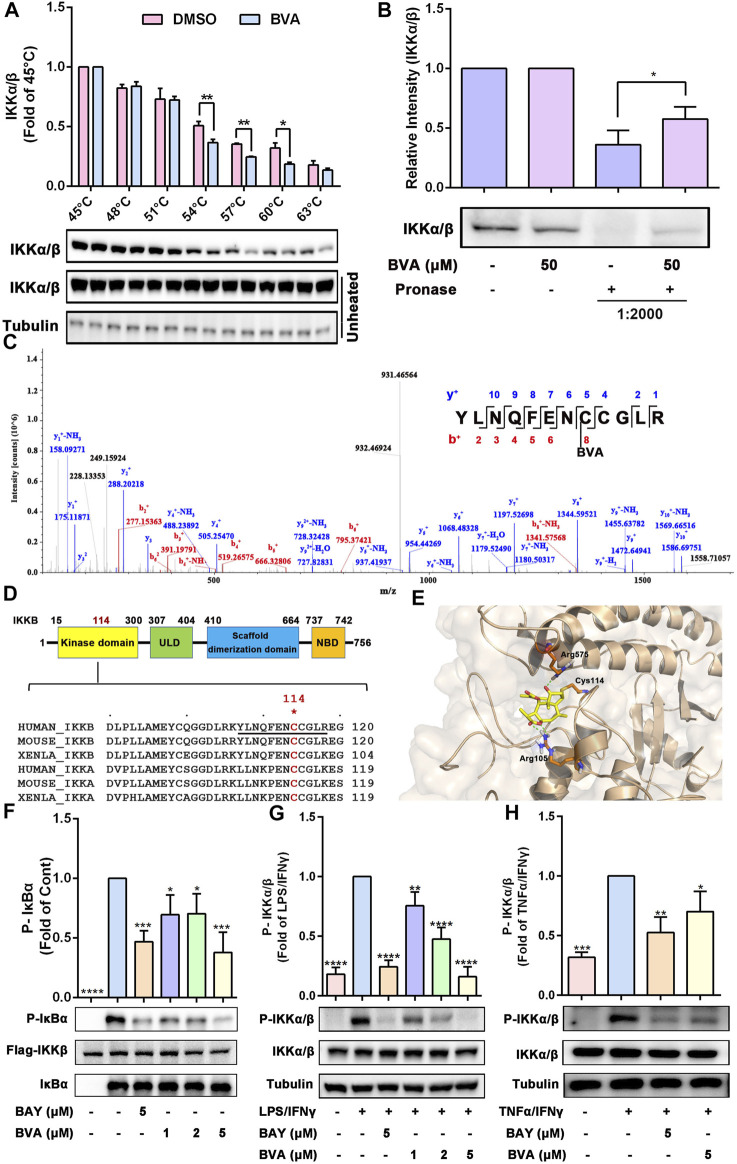
Brevilin A directly binds IKKα/β to inhibiting their activation. **(A)** The effects of BVA on the thermal stability of IKKα/β was analyzed by CETSA assay. The top panel shows the quantification of the thermal stability of IKKα/β in different temperature. The optical density of IKKα/β was normalized with respect to those obtained at 45°C. **(B)** The effects of BVA on the proteolytic susceptibility of IKKα/β was analyzed by DARTS assay. The densitometry analysis of IKKα/β was shown. **(C)** LC-MS/MS analysis of the recombinant human IKKβ protein incubated with BVA for 8 h at 4°C. The covalent binding site is shown. **(D)** The top panel shows the schematic diagram of domains in IKKβ. The bottom panel shows the sequence comparison analysis of IKKα/β from different species and the Cys114 is highlighted in red. **(E)** Molecular docking of BVA with IKKβ (PDB: 3RZF). The covalent bond and hydrogen bond are shown. **(F)** Purified human Flag-IKKβ was used as the kinase, recombinant human IκBα was used as the substrate. Immunoblotting using the p-IκBα antibody reflects the effect of BVA on the kinase activity of IKKβ. **(G,H)** RAW264.7 cells were treated with BVA and BAY for 2 h, and followed by stimulating with LPS/IFNγ and TNFα/IFNγ, respectively. The phosphorylated and total IKKα/β as well as Tubulin were detected by western blotting. The quantitative results of the ratio of p-IKKα/β normalized to IKKα/β are shown. *p < 0.05, **p < 0.01, ***p < 0.001, ****p < 0.0001, vs. vehicle, LPS/IFNγ, or TNFα/IFNγ.

To explore the most likely protein targets of brevilin A associated with the NF-κB pathway, the online tool SwissTargetPrediction was utilized for target prediction in this study. As shown in Supplementary Table S2, brevilin A can directly target IKKβ, which is a key upstream kinase that control the activation of NF-κB signaling via phosphorylating IκBα (Liu et al., 2017; Durand and Baldwin, 2017). A large number of evidences have proved that IKKβ is a promising anti-inflammatory drug target (Durand and Baldwin, 2017). To verify the predicted result, we performed CETSA and DARTS to examine the direct interaction of brevilin A and IKKβ. As expected, brevilin A (50 μM) treatment decrease the thermal stability of IKKα/β at 54–60°C (Figure 6A), and decreased the protease sensitivity of IKKα/β (Figure 6B) in RAW264.7 cell lysates, as compared with DMSO. Furthermore, CETSA assay also showed that brevilin A treatment could not obviously alter the thermal stability of IκBα (Supplementary Figure S6), another important signaling molecule in NF-κB pathway. CETSA and DARTS, two valuable tools for the validation of drug targets, are based on the biophysical principle of ligand-induced thermal shift or reduction in protease sensitivity of target proteins, respectively (Lomenick et al., 2009; Martinez Molina et al., 2013). According to these two principles, the present results both suggest a direct interaction between brevilin A and IKKα/β.

Because brevilin A possesses a α, β-unsaturated ketone moiety, which may act as a Michael acceptor and may thus react with the active cysteine (Cys) of its protein target(s) *via* a covalent bond. To evaluate which cysteine residue was covalently modified by brevilin A, we incubated the recombinant human IKKβ protein with brevilin A, followed by LC-MS/MS assay. As shown in Figure 6C, data analysis of tryptic reaction products identified a peptide (YLNQFENCCGLR) with a calculated mass of 1805.74 Da. MS/MS spectrometry of this peptide revealed that a 346.09 Da mass shift occurred starting from the y4 to the y5 fragment ions (Figure 6C). The mass difference of 346.09 Da exactly matches the molecular weight of one molecule of brevilin A, indicating that the Cys114 residue was covalently modified by brevilin A. A sequence comparison in Figure 6D suggests that the Cys114 residue is conserved in IKKα/β among various species, including human and mouse. Molecular docking simulation represented that the α, β-unsaturated carbonyl group of brevilin A covalently bound to the reactive sulfur atom of Cys114 of IKKβ by Michael addition (Figure 6E). Furthermore, two hydrogen bonds were formed between C4 carbonyl of brevilin A and the amino-group of Arg575, as well as C12 carbonyl of brevilin A and the amino-group of Arg105 (Figure 6E). The binding energy of brevilin A and IKKβ was −29.187 kcal/mol.

To further assess this binding event whether altered the kinase activity of IKKβ, we performed an *in vitro* kinase assay using purified human Flag-IKKβ as the kinase, and recombinant human IκBα as the substrate. As shown in Figure 6F, pre-treated with brevilin A significantly suppressed human Flag-IKKβ-mediated phosphorylation of IκBα *in vitro*. Furthermore, in RAW264.7 cells, we also found that brevilin A obviously attenuated LPS/IFNγ- (Figure 6G) and TNFα/IFNγ-induced phosphorylation of IKKα/β (Figure 6H), which represents the activation state of IKKα/β. Consistent with the result obtained from RAW264.7 cells, brevilin A also suppressed IKKα/β phosphorylation in lung tissues in LPS-exposed mice (Supplementary Figure S7). Additionally, DTT-treated brevilin A, who lost the covalent binding ability to the active cysteines, could not inhibit the LPS/IFNγ-induced activation of IKKα/β in RAW264.7 cells (Supplementary Figure S8). Taken together, these results indicate that brevilin A might covalently modify the Cys114 of IKKα/β via a Michael addition, thereby inhibiting the activity and function of IKKα/β.

## 4 Discussion

Conserved NF-κB pathway functions as a master regulator in the innate immune and inflammatory response by regulating the transcription of a series of target genes, including cytokines, chemokines, and pro-inflammatory enzymes (Liu et al., 2017). Abnormal hyperactivation of NF-κB pathway is associated with various inflammatory diseases, including ALI (Fan et al., 2001). IKKα/β are the key upstream kinases for the phosphorylation of IκBα, which is responsible for the activation of NF-κB signaling. Hence, pharmacological inhibition of IKKα/β holds promise for the treatment of NF-κB-driven inflammatory diseases (Durand and Baldwin, 2017; Chen et al., 2019). In this study, we demonstrated that a natural product, brevilin A, act as a potent inhibitor of IKKα/β, thereby blocking the activation of NF-κB signaling *in vitro* and *in vivo*.

Recently, sesquiterpene lactones, a class of natural products with excellent anti-inflammatory and anti-tumor activities, have received growing attention from the scientific community (Shulha and Zidorn, 2019; García Manzano et al., 2020). Brevilin A is an active sesquiterpene lactone isolated from *C. minima*, and has been proved to possess anti-inflammatory, anti-cancer, anti-viral, and anti-oxidative effects (Chen et al., 2013; Zhang et al., 2019; Khan et al., 2020; Zhou et al., 2020; Qin et al., 2021). Although brevilin A has been shown to repress LPS-induced neuroinflammation via inhibition of NF-κB signaling pathway and NOD-like receptor family pyrin domain-containing 3 (NLRP3) inflammasome activation (Zhou et al., 2020; Qin et al., 2021), its precise molecular targets and mode of action are still uncharacterized. In this study, using SwissTargetPrediction database, we found that TLR9 and IKKβ were the most likely target proteins of brevilin A. TLR9 is responsible for the recognition of single-stranded DNA CpG containing unmethylated CpG motifs derived from DNA viruses (such as human papilloma virus and herpes simplex virus) and bacteria (such as *Salmonella enterica* subsp. and *Mycobacterium tuberculosis*) (Lind et al., 2021), which are not the main pathogens of ALI. Furthermore, we also found that brevilin A effectively blocked LPS/IFNγ- or TNFα/IFNγ-induced inflammatory response in macrophages. Whereas LPS and TNFα are both potent activators of NF-κB pathway. Therefore, we focused on IKKα/β, the key upstream kinases of NF-κB pathway.

Using a CETSA and DARTS assay, we found that brevilin A not only decreased the thermal stability of IKKα/β, but also inhibited the proteolysis of IKKα/β by the pronase subtilisin. CETSA is an approach to validate the direct bind of small molecule and its target protein by detecting the melt curve of target protein which can shift to a higher or lower temperature when bound by its ligand (Martinez Molina et al., 2013; Gao et al., 2021). DARTS is another universally applicable strategy to investigate ligand-target interactions, which takes advantage of a reduction in the protease susceptibility of the target protein upon ligand binding (Lomenick et al., 2009; Yuan et al., 2022). The results of CETSA and DARTS both suggested the direct binding capability of brevilin A to IKKα/β. Furthermore, CETSA analysis also showed that brevilin A could not obviously alter the thermal stability of IκBα, another important regulator of NF-κB activation, suggesting the relative specificity of brevilin A to IKKα/β. *In vitro* kinase assay demonstrated that brevilin A effectively inhibited IKKβ-mediated phosphorylation of IκBα, indicating the inhibitory effect of brevilin A on the activity and function of IKKβ. The inhibitory effect of brevilin A on IKKα/β activation was further confirmed in LPS/IFNγ- or TNFα/IFNγ-stimulated RAW264.7 macrophages, as well as LPS-induced ALI in mice. In addition, the overall effect of IKKα/β inhibition by brevilin A was attenuation of ALI in mice undergo intratracheal instillation of LPS, including decreased infiltration of inflammatory cells, reduced synthesis and release of pro-inflammatory cytokines.

Brevilin A contains an α, β-unsaturated ketone moiety, which is electrophilic and thiol reactive, and may act as a Michael acceptor. Consistently, LC-MS result demonstrated that brevilin A formed a covalent bond with the thiol of DTT via Michael addition reaction. Although brevilin A possesses another unsaturated C2’=C3’ double bond, the presence of 5′-methyl may decrease the thiol reactivity of this double bond (Gao et al., 2021). Therefore, LC-MS showed an addition of only one molecule of brevilin A to one molecule of DTT *via* its C2=C3 double bond. More importantly, DTT-pretreated brevilin A was deprived of the inhibitory effects on LPS/IFNγ-induced activation of IKKα/β, phosphorylation and degradation of IκBα, and NO production in RAW264.7 cells. In view of the importance of α, β-unsaturated ketone moiety in the anti-inflammatory activity of sesquiterpene lactones, we thus speculate that the unsaturated C2=C3 double bond of brevilin A is crucial for its anti-inflammatory activity. Furthermore, previous studies have shown that covalently modifying the thiol groups of the reactive cysteines of IKKα/β by small molecule compounds could lead to the inactivation of IKKα/β (Ogino et al., 2004; Heyninck et al., 2014; Dong et al., 2015; Liu et al., 2019). Thus, we also speculated that electrophilic brevilin A may react with thiols of the cysteines in the IKKα/β proteins via Michael addition. Interestingly, LC-MS/MS analysis showed that brevilin A could covalently bind to Cys114 in human IKKβ. Cys114 is conserved in both human and mouse IKKα/β, and locates in the kinase domain of IKKα/β. Structural analysis of IKKβ revealed that Cys114 mediate a critical interaction between the kinase domain (KD) and scaffold dimerization domain (SDD). SDD domain participates in IKKβ dimerization, which plays a critical role for both IKKβ activation and IKKγ binding (Mercurio et al., 1997; May et al., 2000; Xu et al., 2011). More importantly, Leung and colleagues have confirmed that hypochlorous acid (HOCl) oxidizes IKKβ irreversibly at Cys114/115 to cysteic acid in order to decrease the kinase activity and activation of IKKβ, thereby inhibiting NF-κB signaling in human keratinocytes (Leung et al., 2013). According to the crystal structure of IKKβ, compared to Cys115, the thiol group of Cys114 is exposed in the solution, which is easy to covalently modified by brevilin A. Therefore, Cys114, a key residue at the junction between the KD and SDD, has been considered as a druggable site for IKKβ inhibition. Based on these views, we suggested that the covalent modification of Cys114 by brevilin A results in obvious inhibitory effects on the kinase activity of recombinant human IKKβ and activation of IKKα/β in LPS/IFNγ- or TNFα/γ-stimulated murine RAW264.7 cells, as well as LPS-stimulated mice. These data also explain the underlying mechanism of anti-inflammatory activity of brevilin A *in vitro* and *in vivo*.

Previously, brevilin A has been identified as a janus kinases (JAKs) inhibitor, and exhibits a potent repression on signal transducer and activator of transcription 3 (STAT3) signaling and cancer cell growth (Chen et al., 2013; Khan et al., 2020). Because STAT3 is another important transcription factor in the regulation of inflammation (Zhao et al., 2016), inhibition of JAKs/STAT3 signaling may thus contribute to the anti-inflammatory role of brevilin A. Furthermore, JAKs also can activate IKKβ *via* a double-stranded RNA-activated protein kinase (PRK)-dependent manner (Kai et al., 2010). Therefore, we speculated that the inhibition of brevilin A on JAKs activity may also contribute to its inhibition on IKKβ. In addition to the crucial role in the regulation of inflammatory response, hyperactivation of IKKs/NF-κB pathway has been shown to inhibit tumor apoptosis and promote tumor proliferation and invasion (Perkins, 2012), which indicated that the anti-cancer effect of brevilin A is due, at least in part, to its suppression on IKKα/β. Moreover, although several studies have shown that brevilin A treatment results in activation of JNK and p38 (Wang et al., 2018), and inactivation of Akt in cancer cells (Qu et al., 2020), our results showed that brevilin A did not affect LPS/IFNγ-induced activation of MAPKs and Akt in RAW264.7 cells. The different phenotypes of brevilin A on MAPKs and Akt signaling may depend on the variation of cellular contexts and microenvironment.

## 5 Conclusion

**FIGURE 7:**
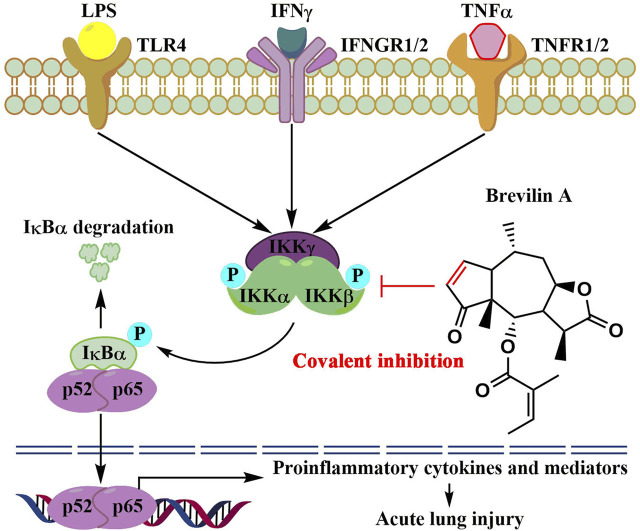
The proposed molecular action model of brevelin A (BVA)-mediated anti-inflammatory activity.

In summary, we report new findings that the natural product, brevilin A, covalently binds to IKKα/β, thereby inhibiting IKKα/β-mediated activation of NF-κB signaling, therefore reducing LPS/IFNγ- or TNFα/IFNγ-induced inflammation *in vitro* and protecting mice against LPS-induced ALI *in vivo* (Figure 7). Our findings also suggest that brevilin A can be used as a promising candidate to develop new therapeutics for human diseases whose pathogenesis involve hyperactivation of IKKs/NF-κB signaling cascade.

## Data Availability

The original contributions presented in the study are included in the article/Supplementary Material, further inquiries can be directed to the corresponding authors.

## Glossary

Akt: RAC-alpha serine/threonine-protein kinases
ALI: acute lung injury
BALF: bronchoalveolar lavage fluid
BMDMs: bone-marrow derived macrophages
BVA: brevilin A
CETSA: cellular thermal shift assay
COX2: cyclooxygenase 2
Cys: cysteine
DARTS: drug affinity responsive target stability
DEX: dexamethasone
DMSO: dimethyl sulfoxide
DTT: dithiothreitol
ELISA: enzyme linked immunosorbent assay
ERK: extracellular regulated protein kinases
IFNγ: interferon γ
IκBα: inhibitor of nuclear factor kappa B alpha
IKKα/β: inhibitor of nuclear factor kappa B kinase α/β
IL-1β: interleukin 1β
iNOS: inducible nitric oxide synthase
JNK: c-Jun N-terminal kinases
LC-MS: Liquid chromatography mass spectrum
LPS: lipopolysaccharide
MAPKs: mitogen-activated protein kinases
MPO: myeloperoxidase
NF-κB: nuclear factor kappa-B
NO: nitric oxide
PGE_2_: prostaglandin-E2
RT: room temperature
RT-qPCR: real time quantitative polymerase chain reaction
TLR4: toll-like receptor 4
TNFα: tumor necrosis factor-α
