# Supplementary figures and images for: Brevilin A Ameliorates Acute Lung Injury and Inflammation Through Inhibition of NF-κB Signaling via Targeting IKKα/β (part 2 of 2)
Source: Front Pharmacol. 2022 Jun 14;13:911157. doi: 10.3389/fphar.2022.911157 (PMC9237443; doi:10.3389/fphar.2022.911157)

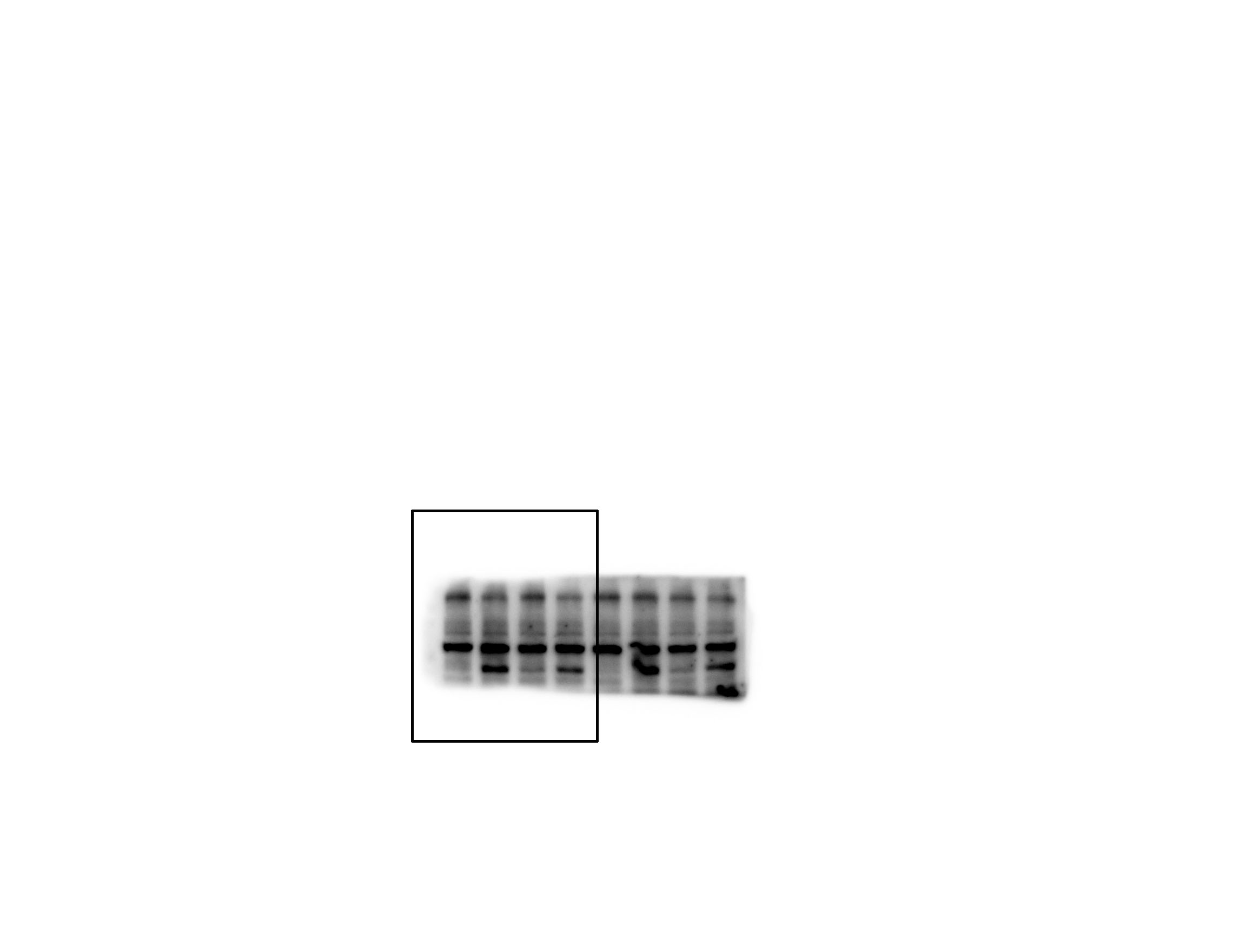

Supplement: Supplementary file 1 [file DataSheet1.ZIP › Original data/Supplementary figure 8-original data/P-IKK-DTT-BVA-3.jpg]
