# Supplementary figures and images for: Brevilin A Ameliorates Acute Lung Injury and Inflammation Through Inhibition of NF-κB Signaling via Targeting IKKα/β (part 1 of 2)
Source: Front Pharmacol. 2022 Jun 14;13:911157. doi: 10.3389/fphar.2022.911157 (PMC9237443; doi:10.3389/fphar.2022.911157)

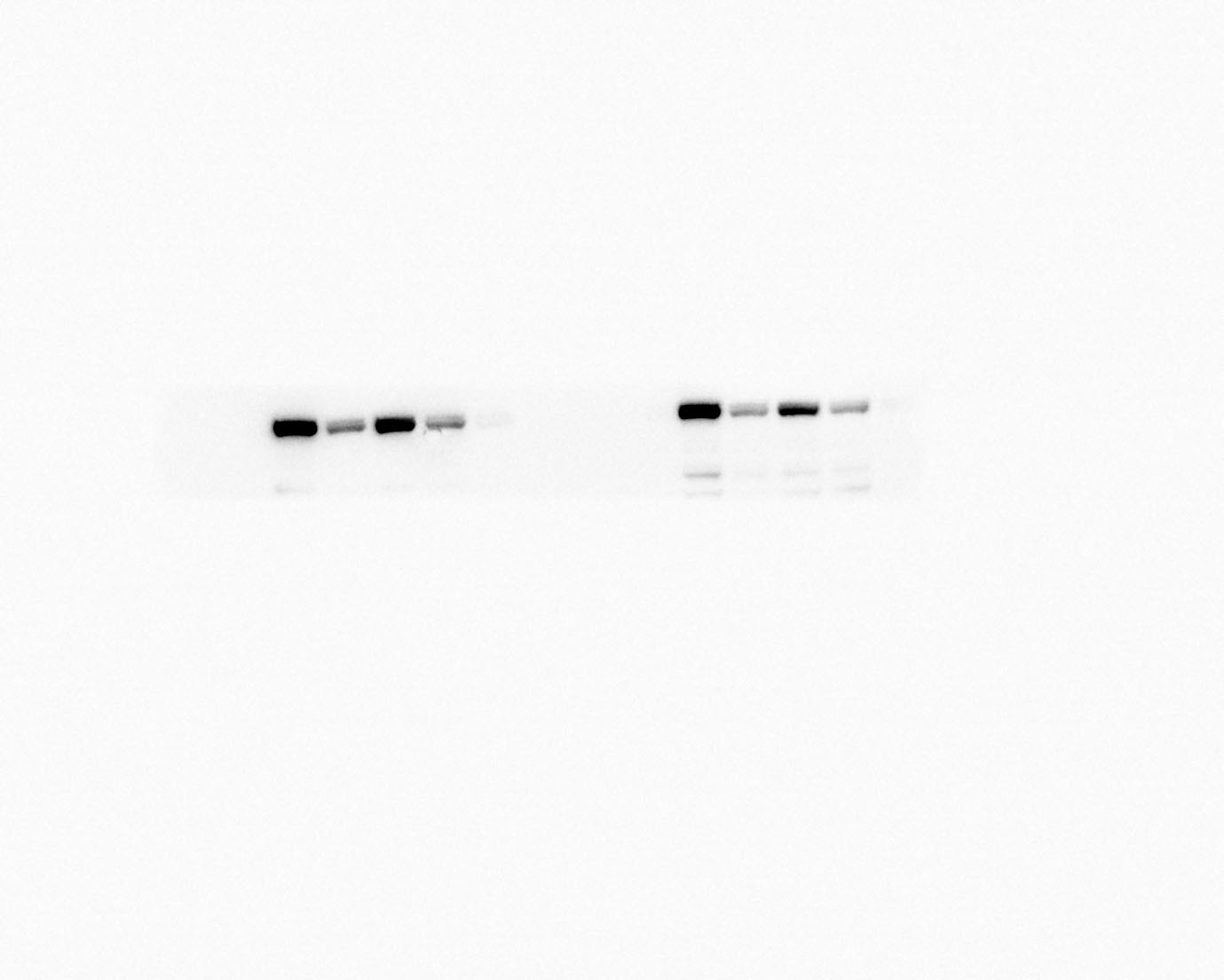

Supplement: Supplementary file 1 [file DataSheet1.ZIP › Original data/figure1-original data/COX2-BVA-1,2.jpg]

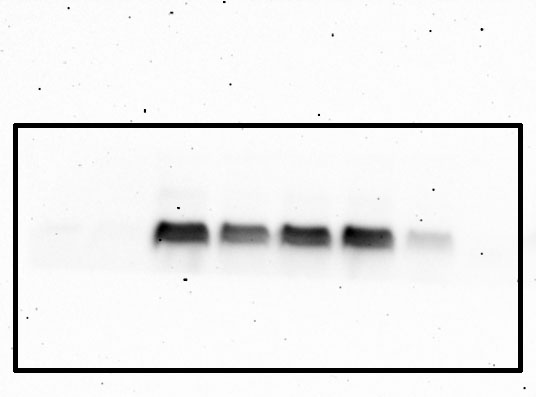

Supplement: Supplementary file 1 [file DataSheet1.ZIP › Original data/figure1-original data/COX2-BVA-3.jpg]

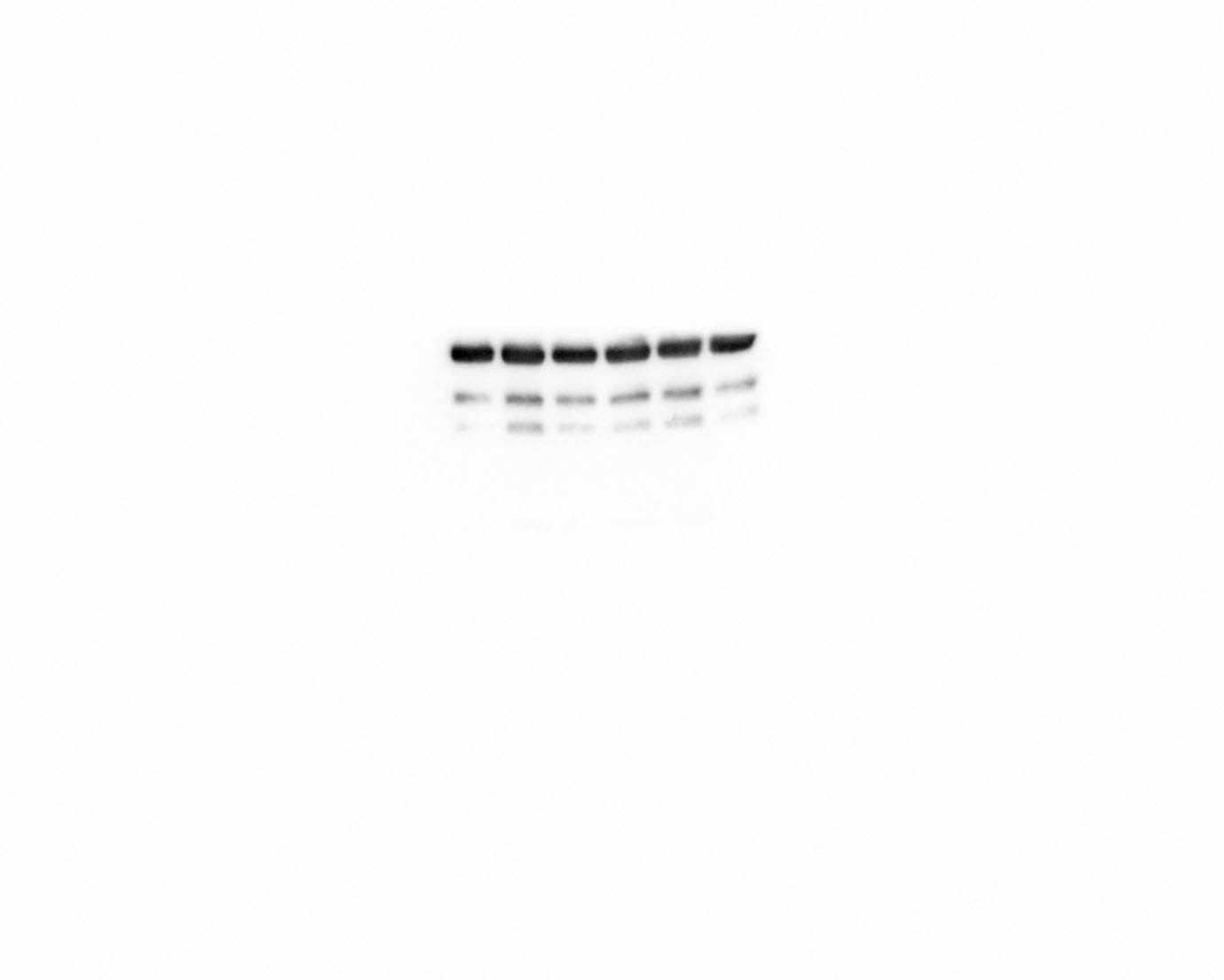

Supplement: Supplementary file 1 [file DataSheet1.ZIP › Original data/figure1-original data/GAPDH-INOS-COX2-3.jpg]

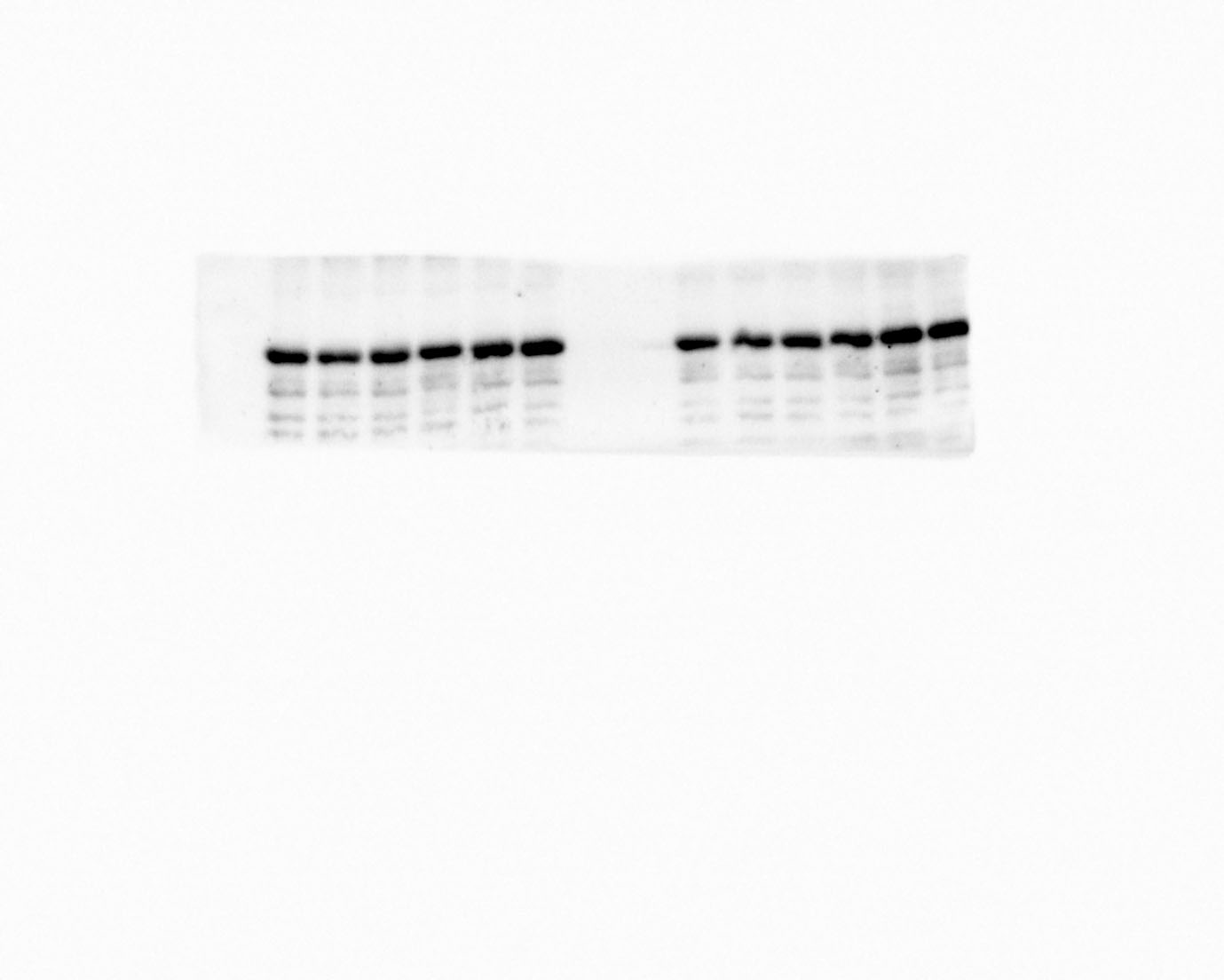

Supplement: Supplementary file 1 [file DataSheet1.ZIP › Original data/figure1-original data/GAPDH-INOS-COX2-BVA-1,2.jpg]

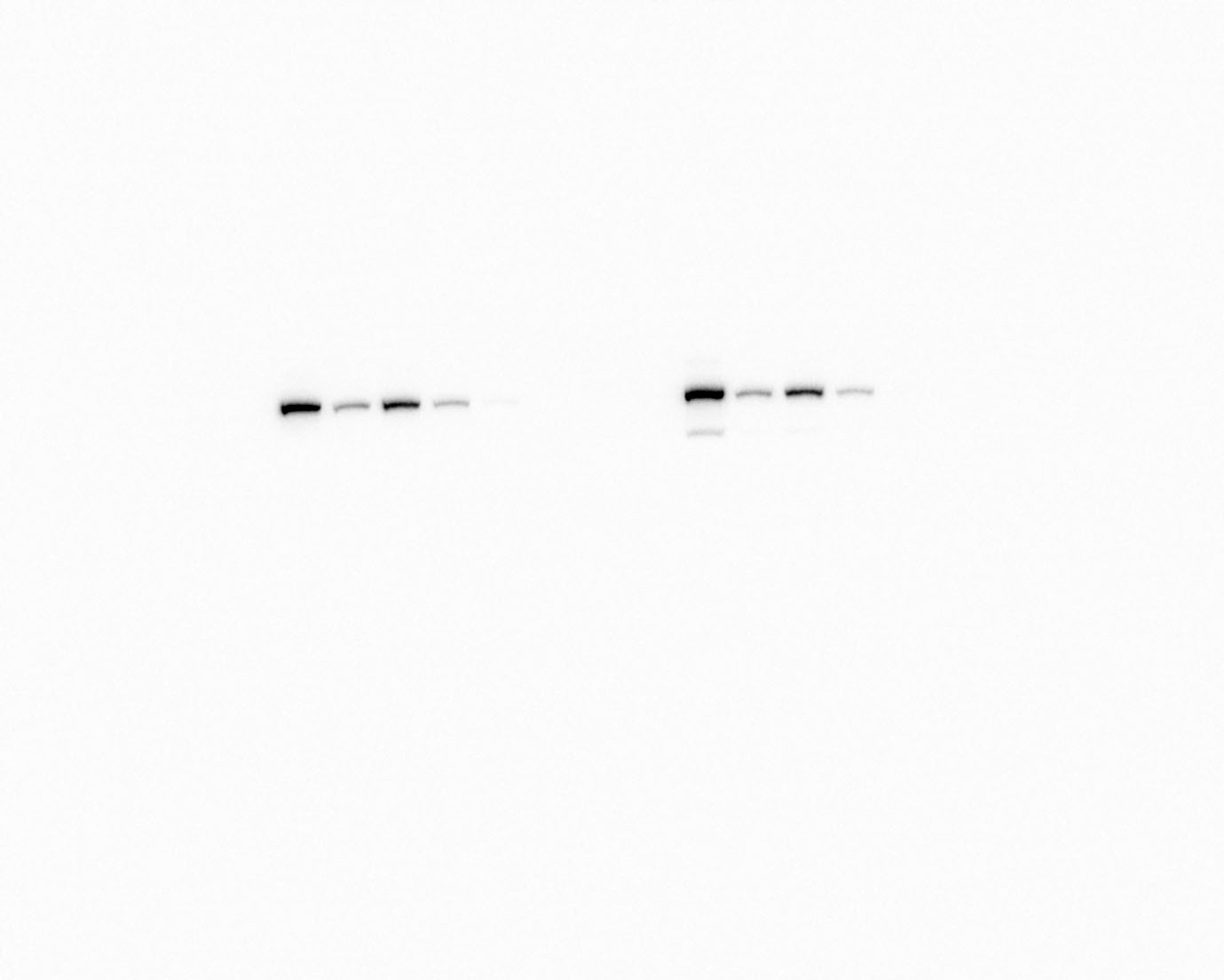

Supplement: Supplementary file 1 [file DataSheet1.ZIP › Original data/figure1-original data/INOS-BVA-1,2.jpg]

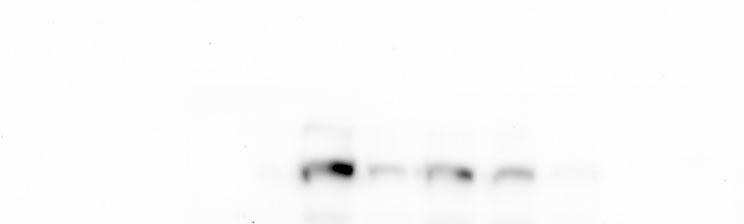

Supplement: Supplementary file 1 [file DataSheet1.ZIP › Original data/figure1-original data/INOS-BVA-3-figure.jpg]

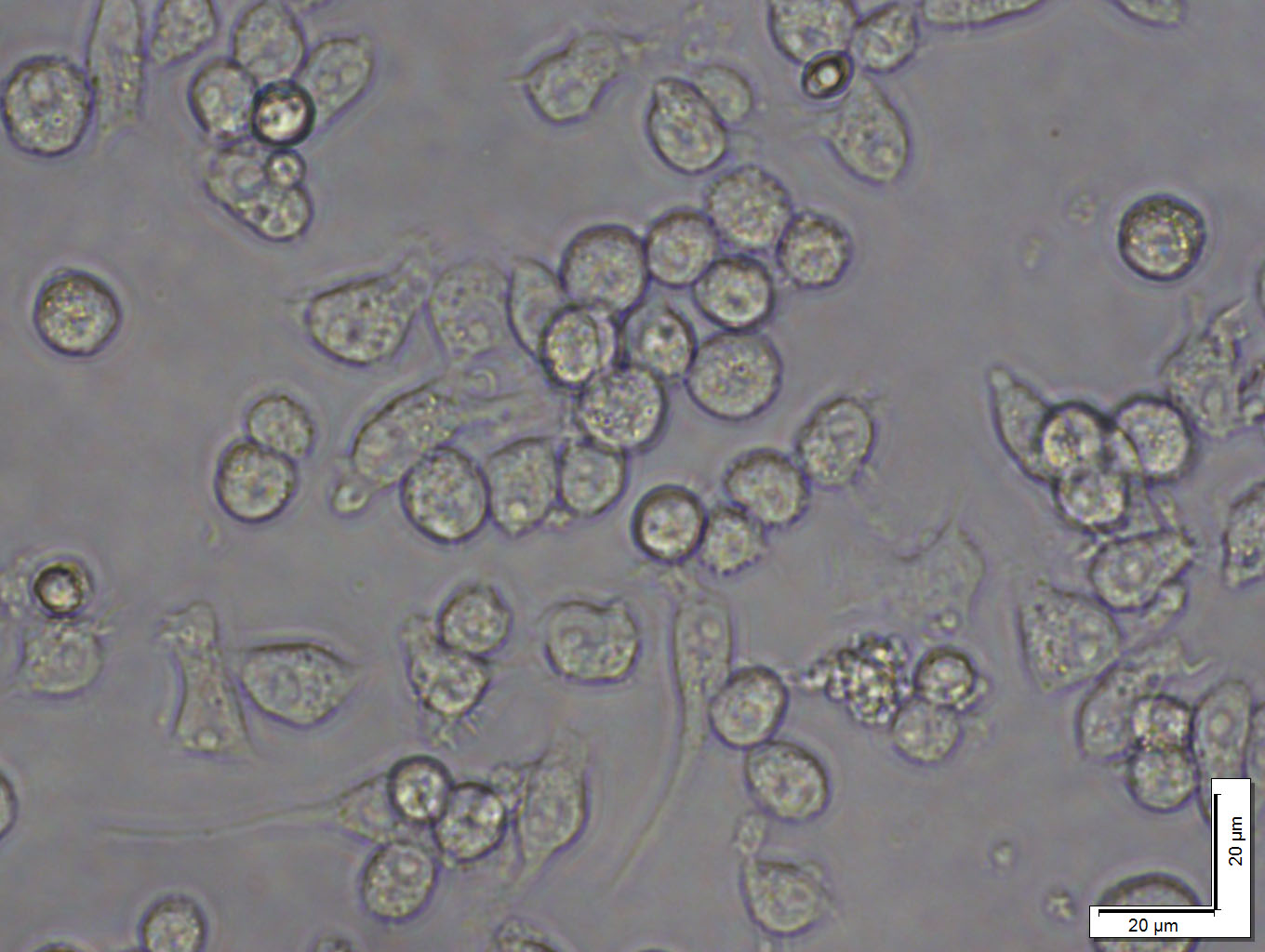

Supplement: Supplementary file 1 [file DataSheet1.ZIP › Original data/figure1-original data/Morphology/BVA-1.jpg]

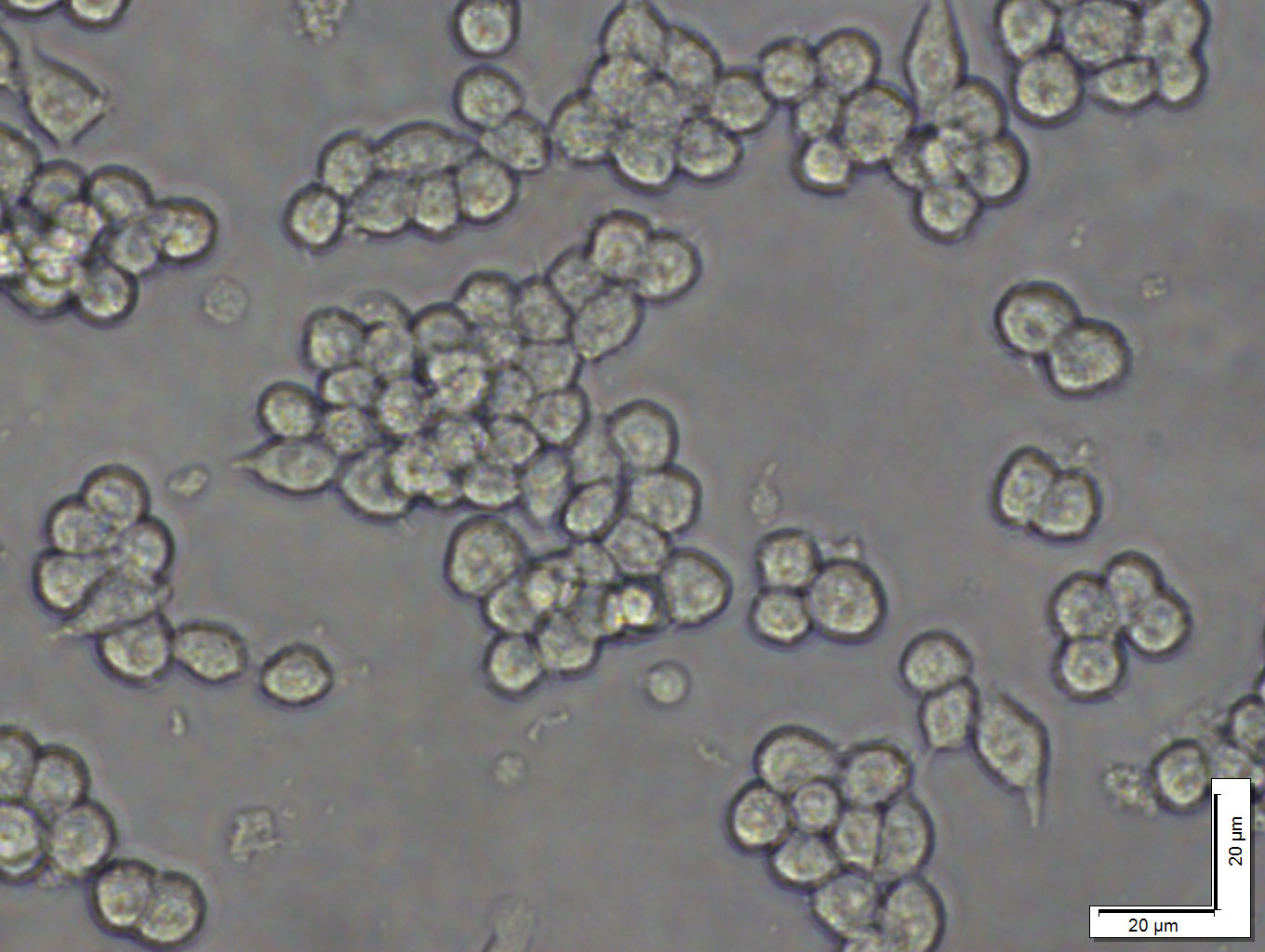

Supplement: Supplementary file 1 [file DataSheet1.ZIP › Original data/figure1-original data/Morphology/CK-1.jpg]

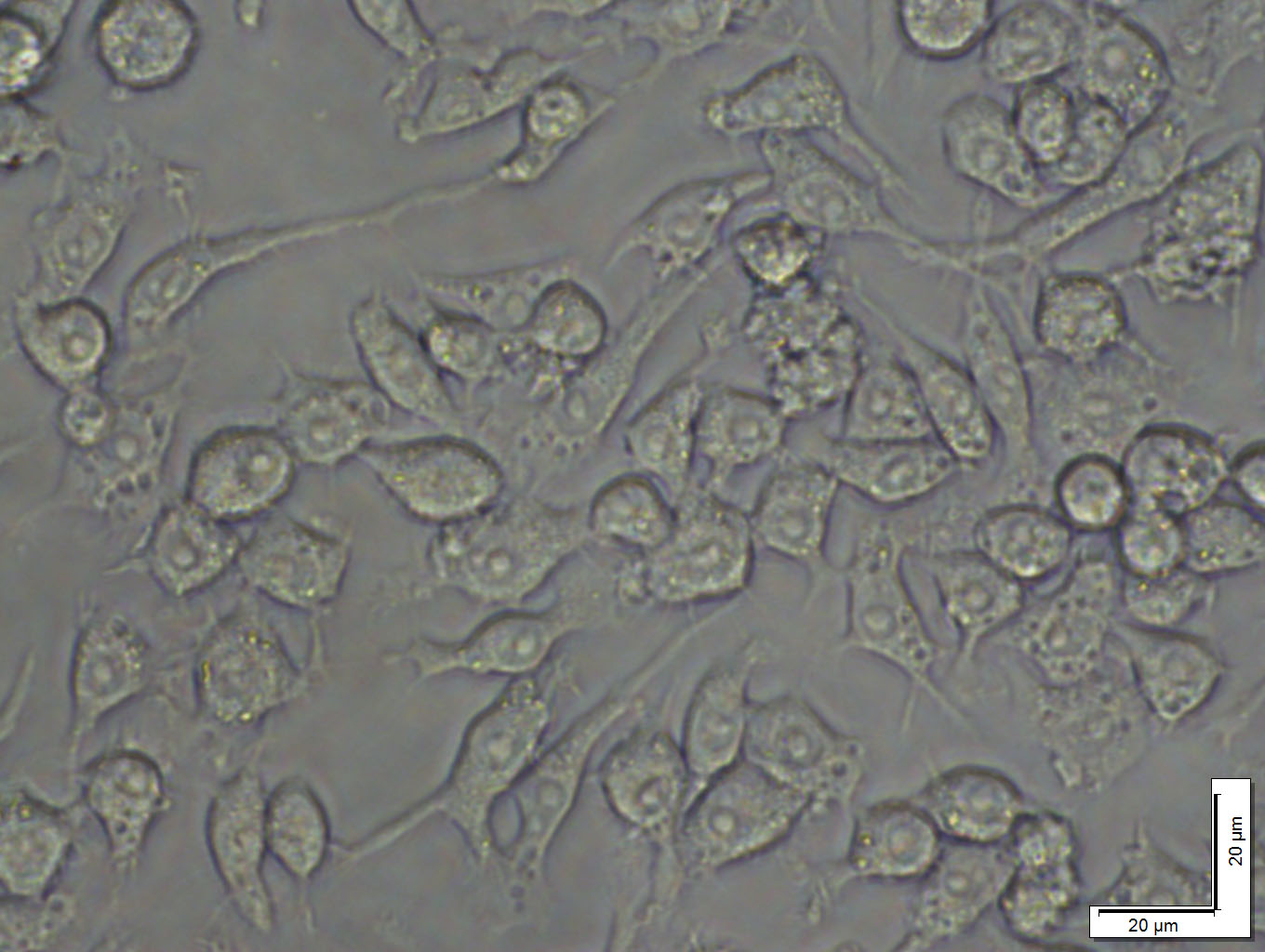

Supplement: Supplementary file 1 [file DataSheet1.ZIP › Original data/figure1-original data/Morphology/LPS-3.jpg]

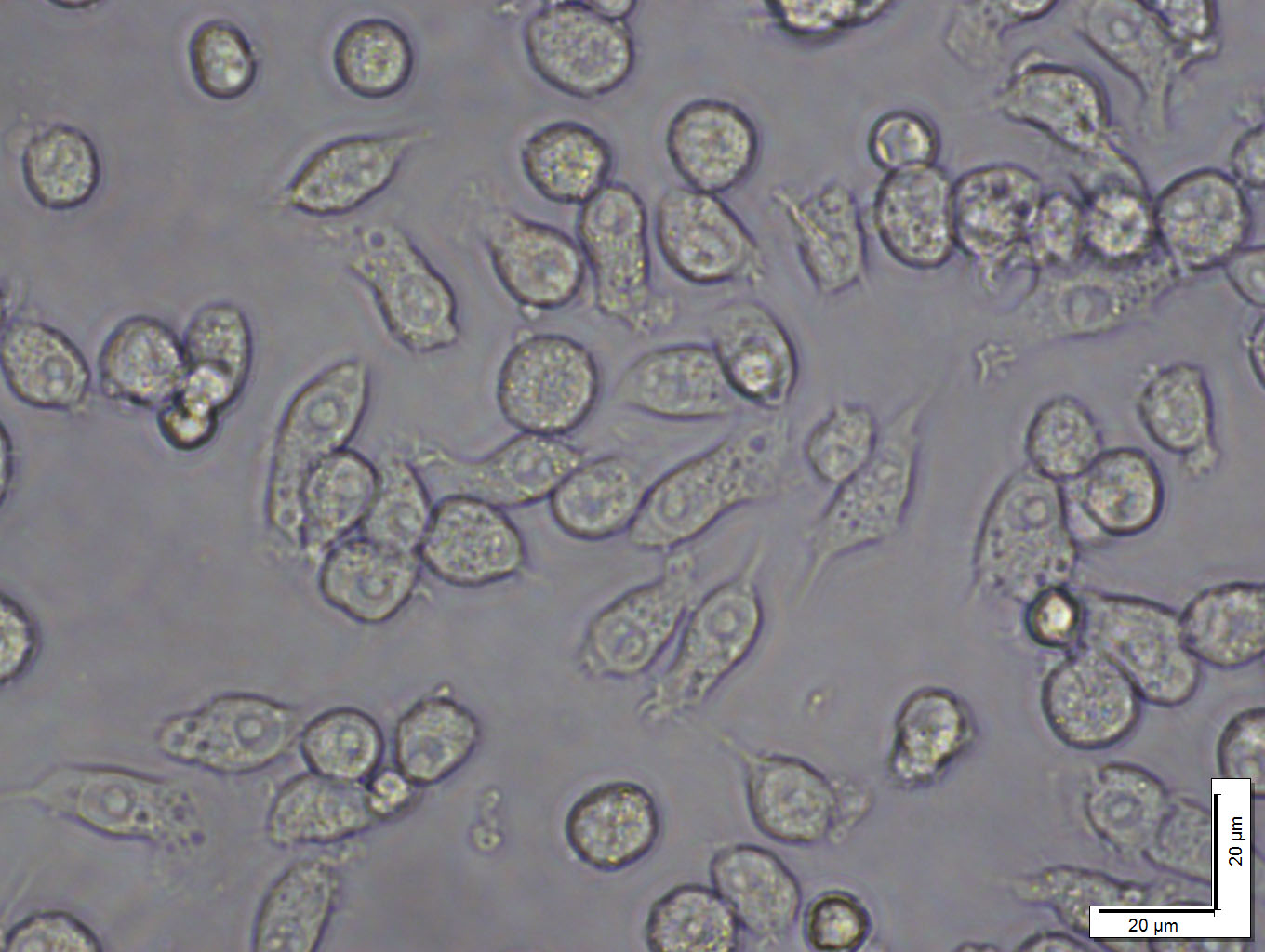

Supplement: Supplementary file 1 [file DataSheet1.ZIP › Original data/figure1-original data/Morphology/PC-1.jpg]

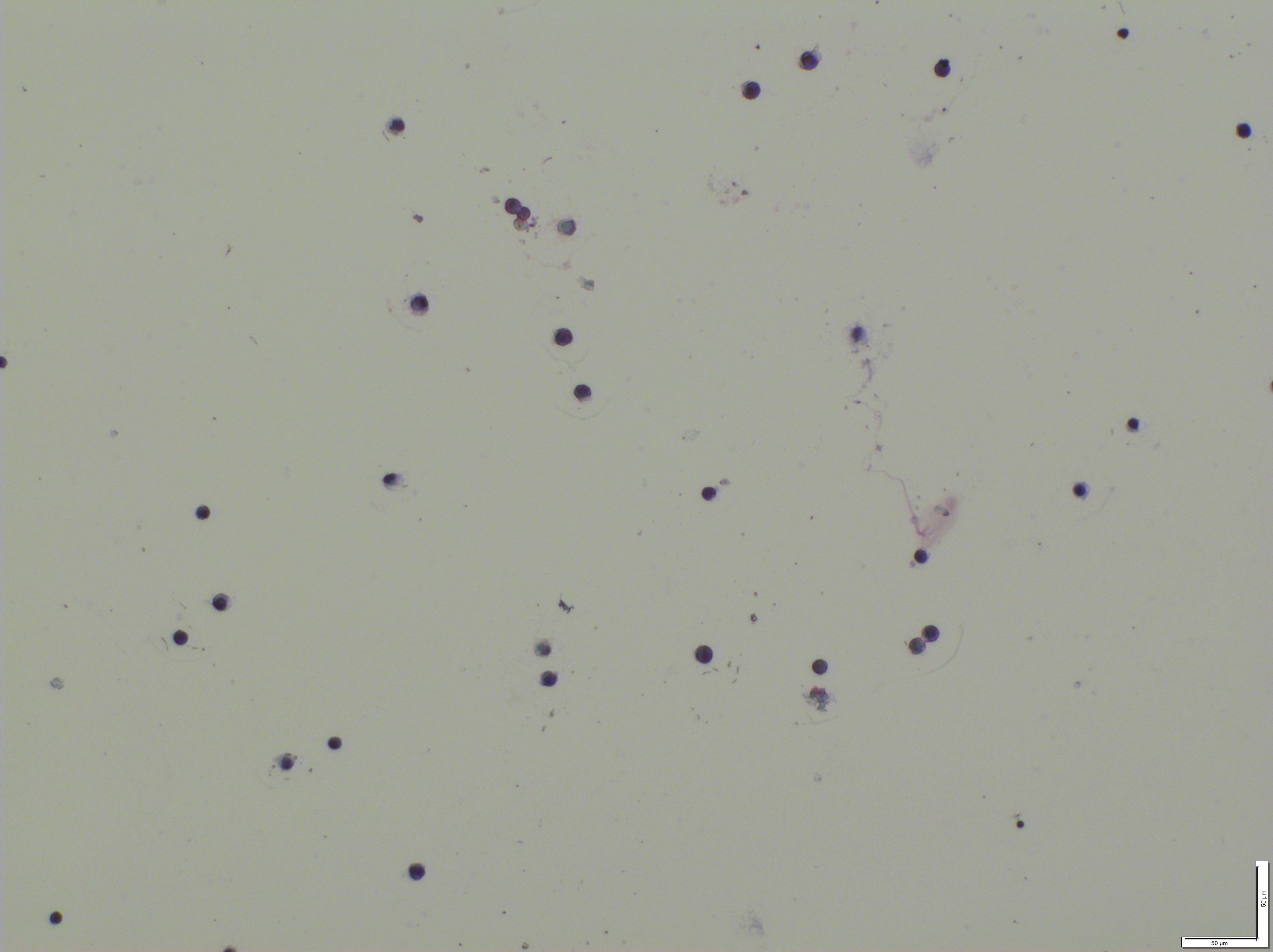

Supplement: Supplementary file 1 [file DataSheet1.ZIP › Original data/figure2-original data/BALF/Cont.jpg]

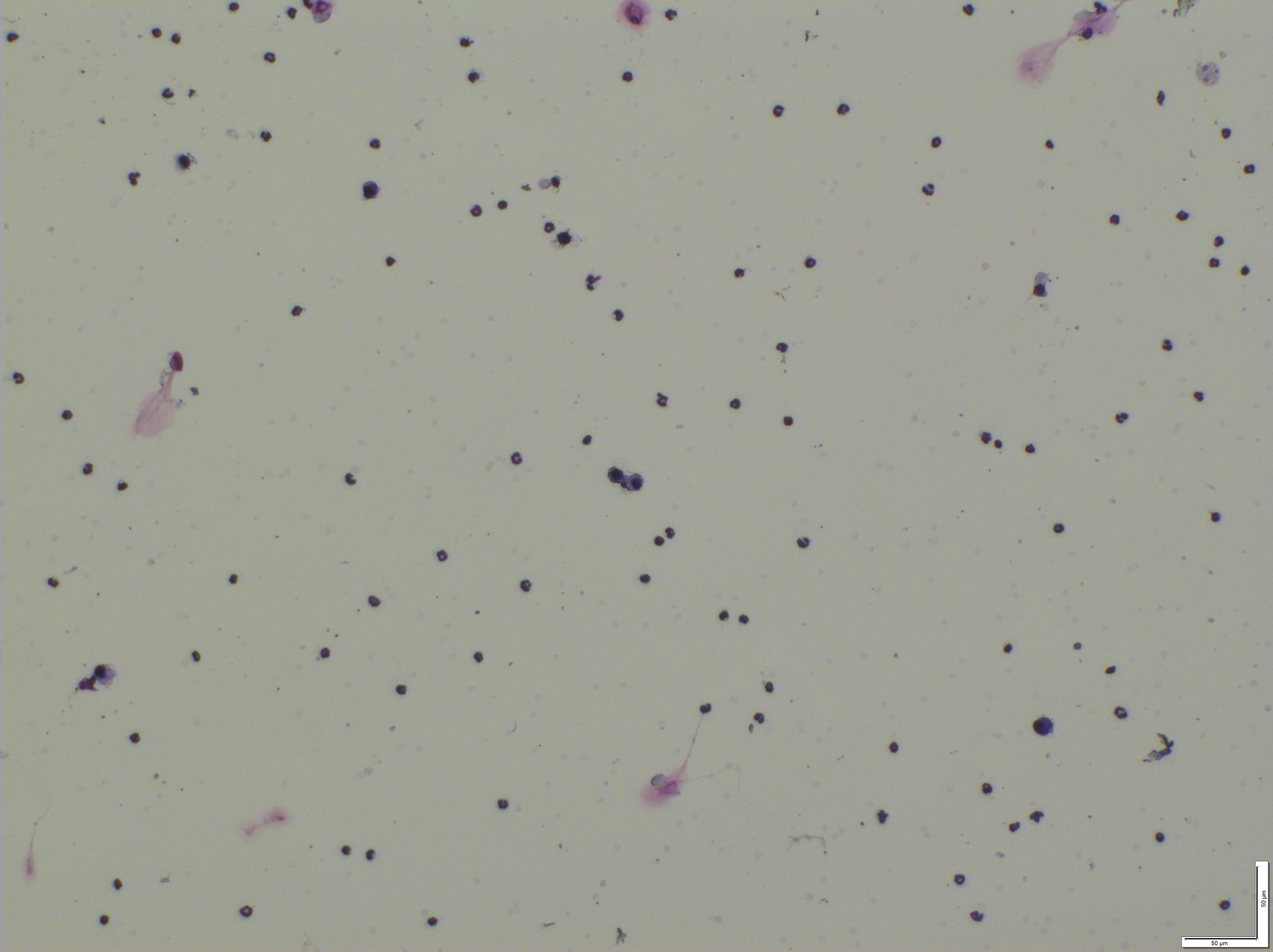

Supplement: Supplementary file 1 [file DataSheet1.ZIP › Original data/figure2-original data/BALF/LPS+BVA.jpg]

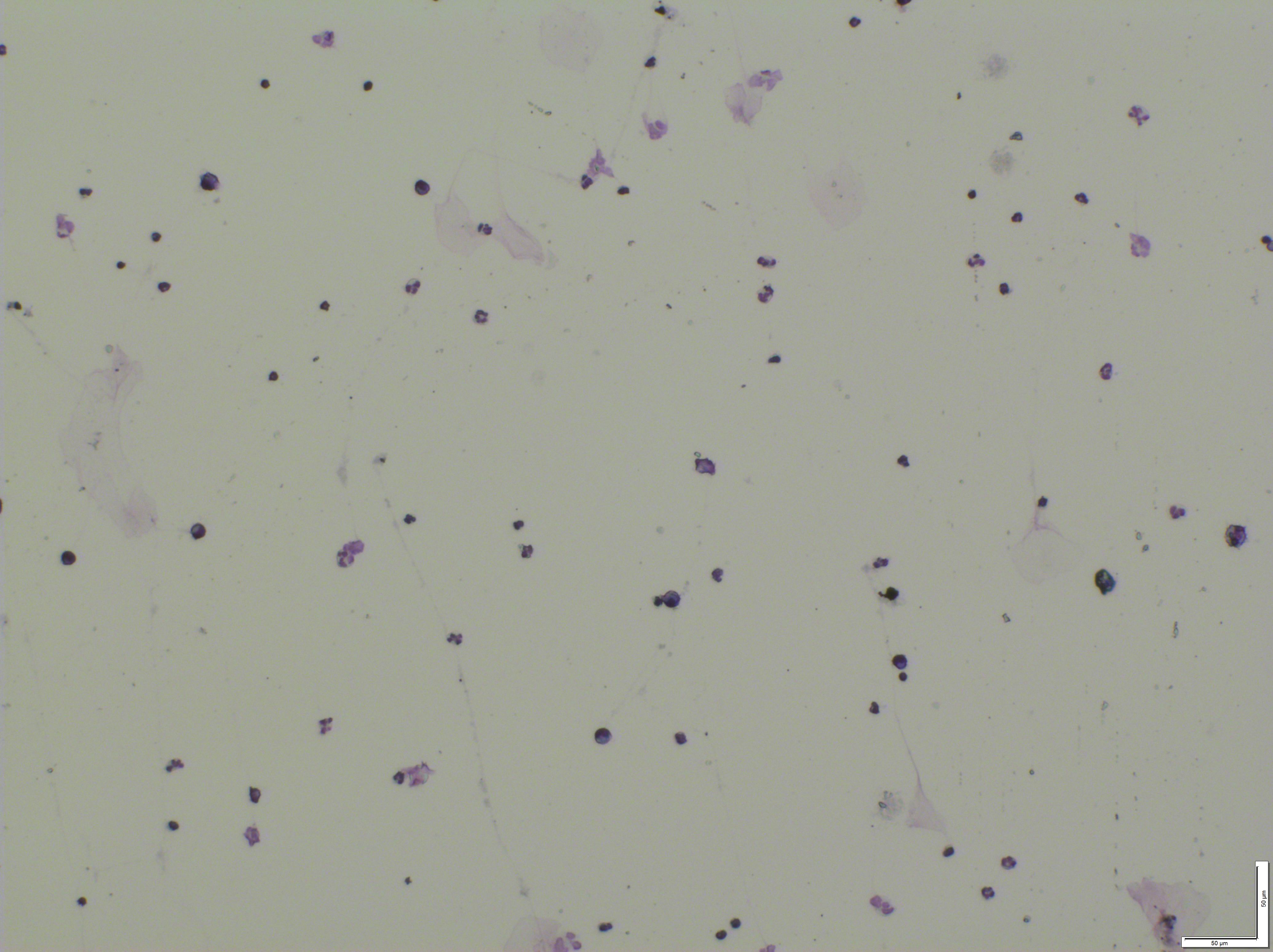

Supplement: Supplementary file 1 [file DataSheet1.ZIP › Original data/figure2-original data/BALF/LPS+DEX.jpg]

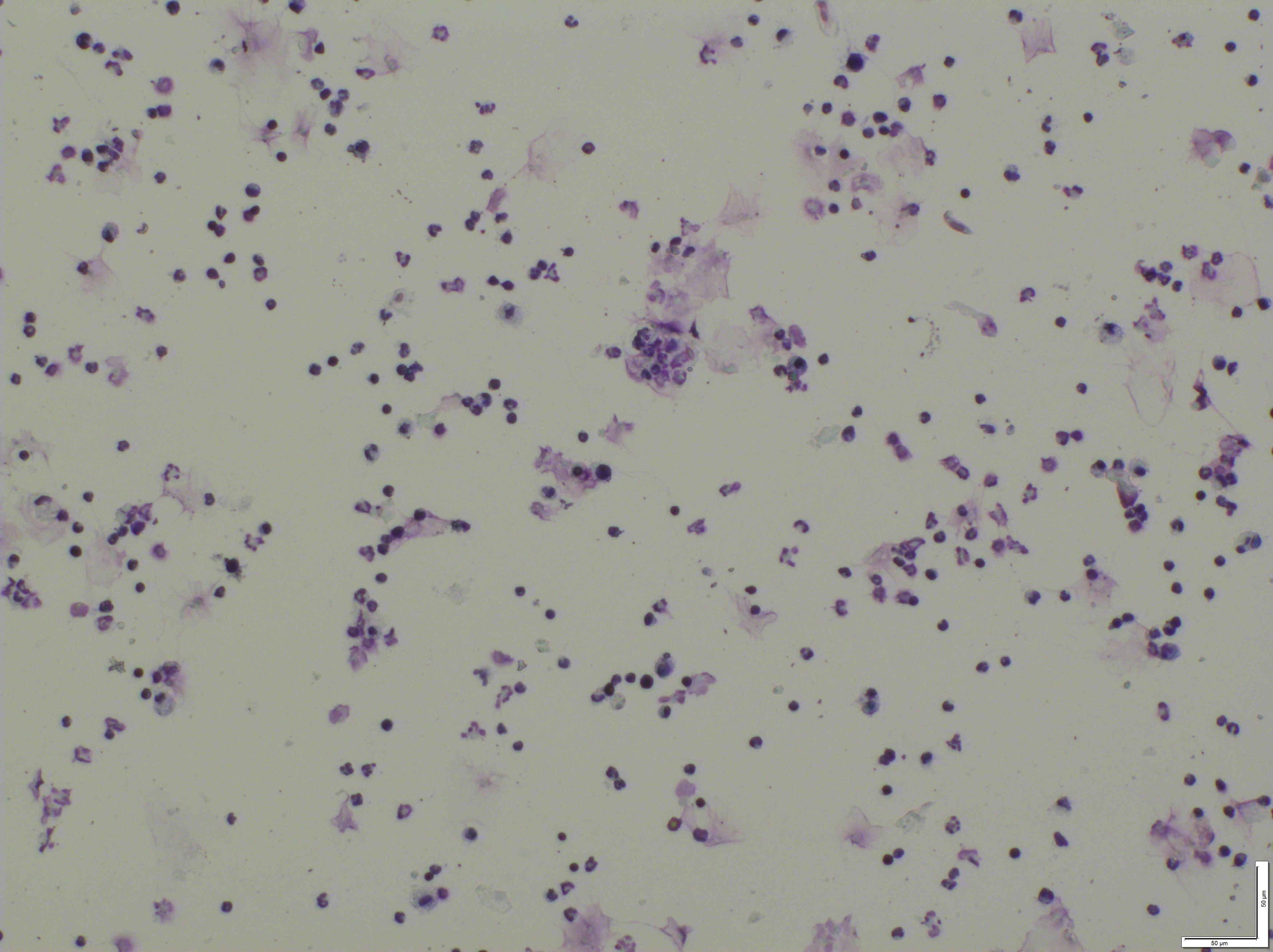

Supplement: Supplementary file 1 [file DataSheet1.ZIP › Original data/figure2-original data/BALF/LPS.jpg]

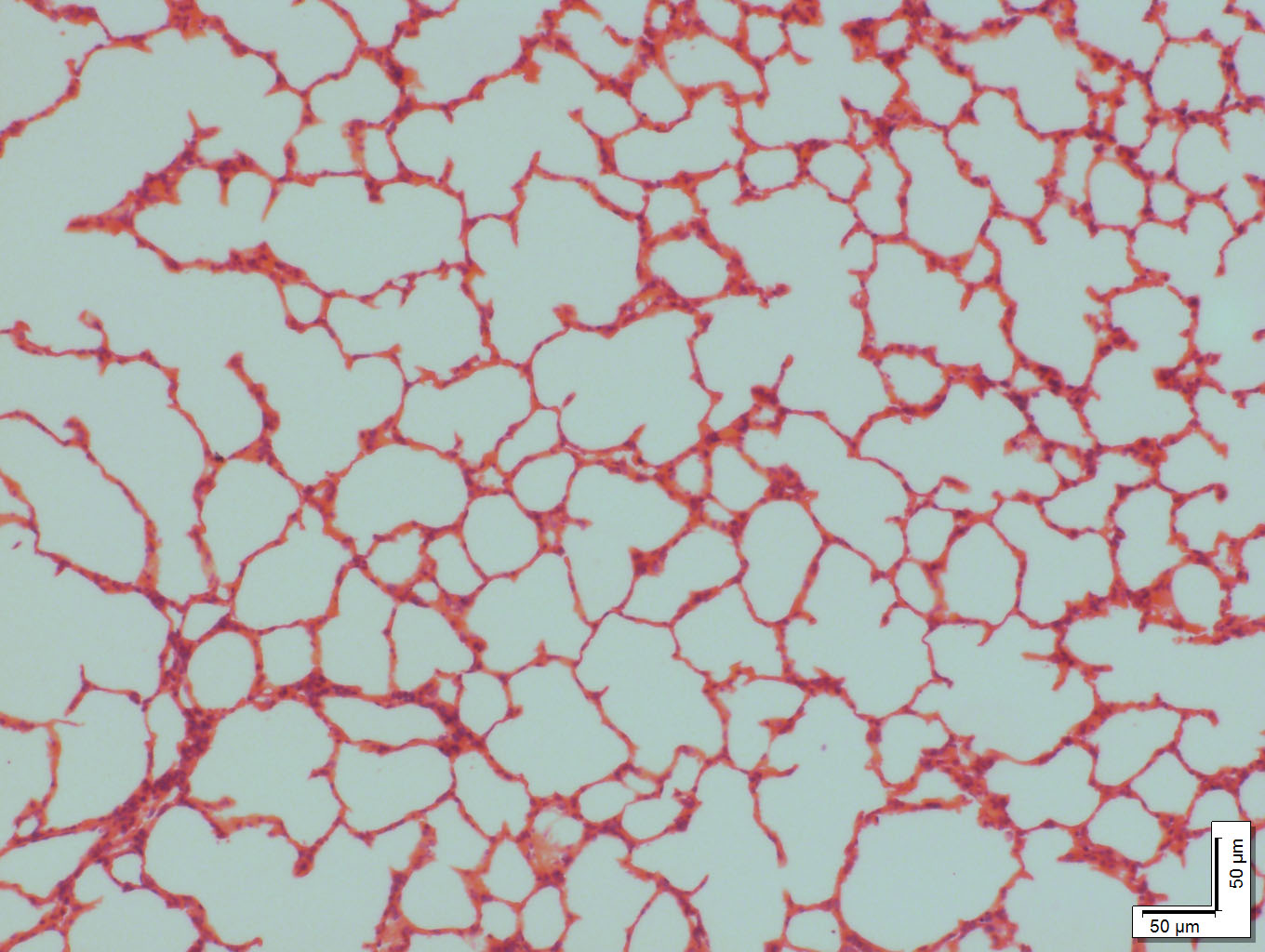

Supplement: Supplementary file 1 [file DataSheet1.ZIP › Original data/figure2-original data/HE/Cont.jpg]

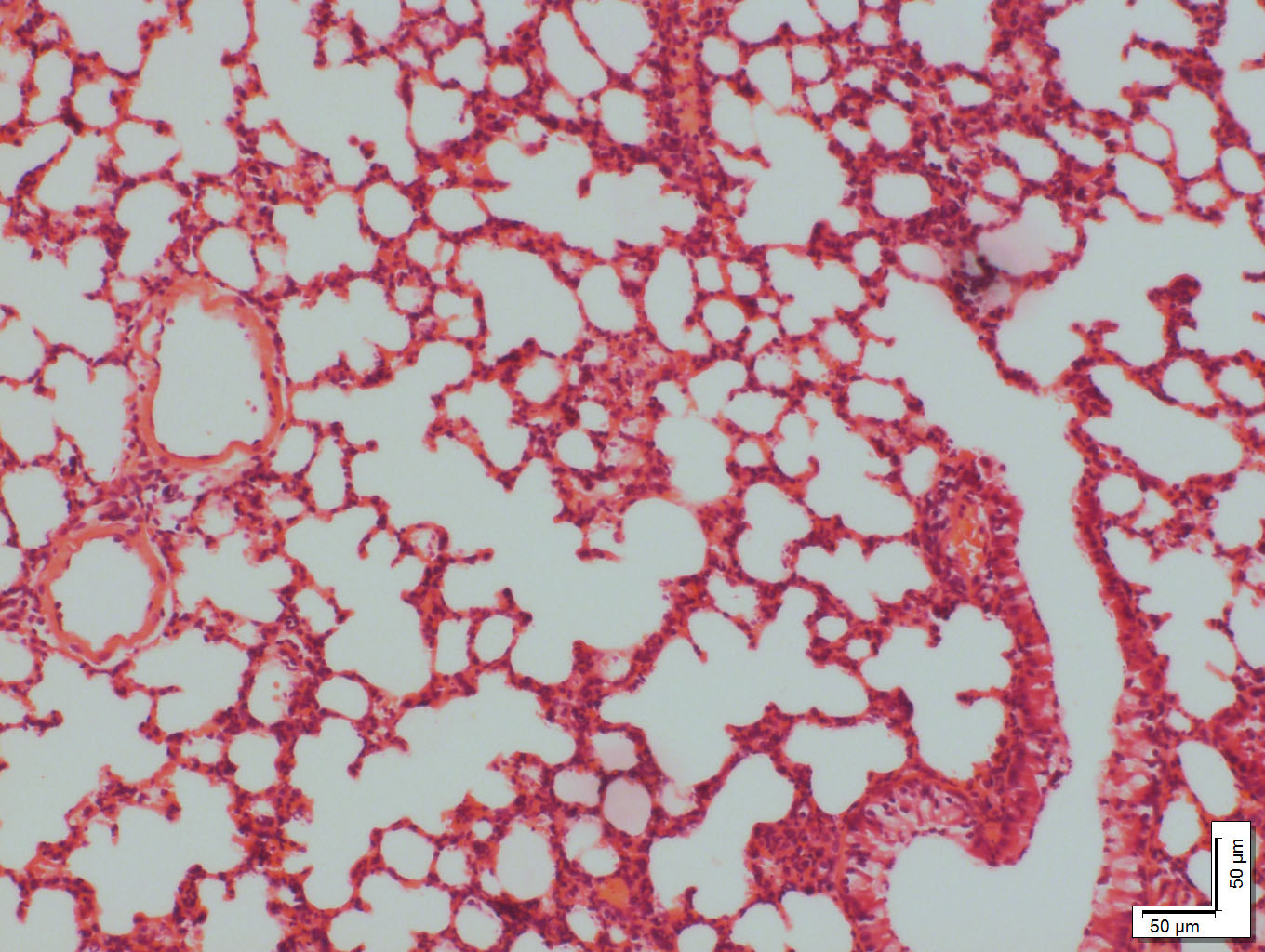

Supplement: Supplementary file 1 [file DataSheet1.ZIP › Original data/figure2-original data/HE/LPS+BVA.jpg]

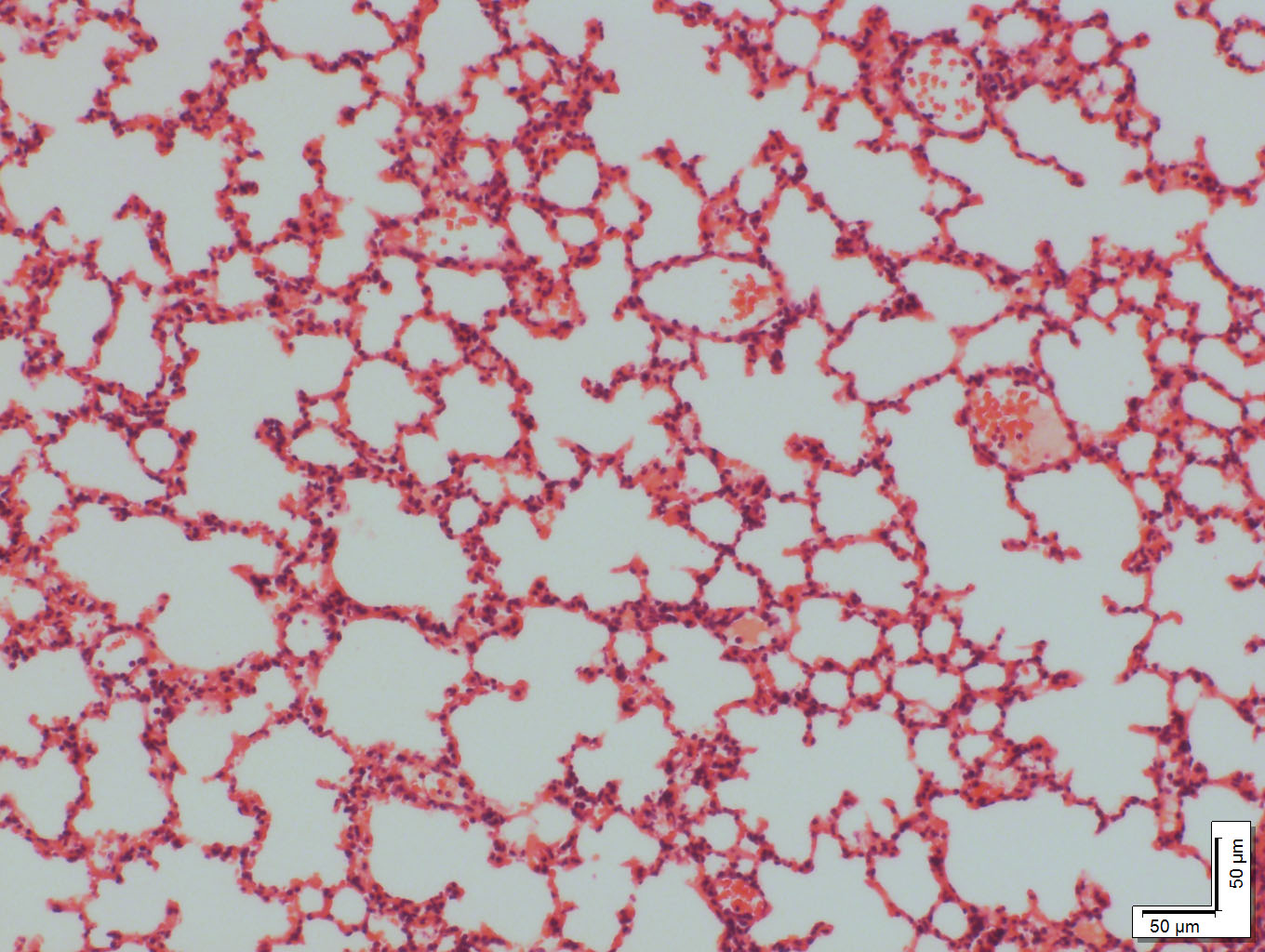

Supplement: Supplementary file 1 [file DataSheet1.ZIP › Original data/figure2-original data/HE/LPS+DEX.jpg]

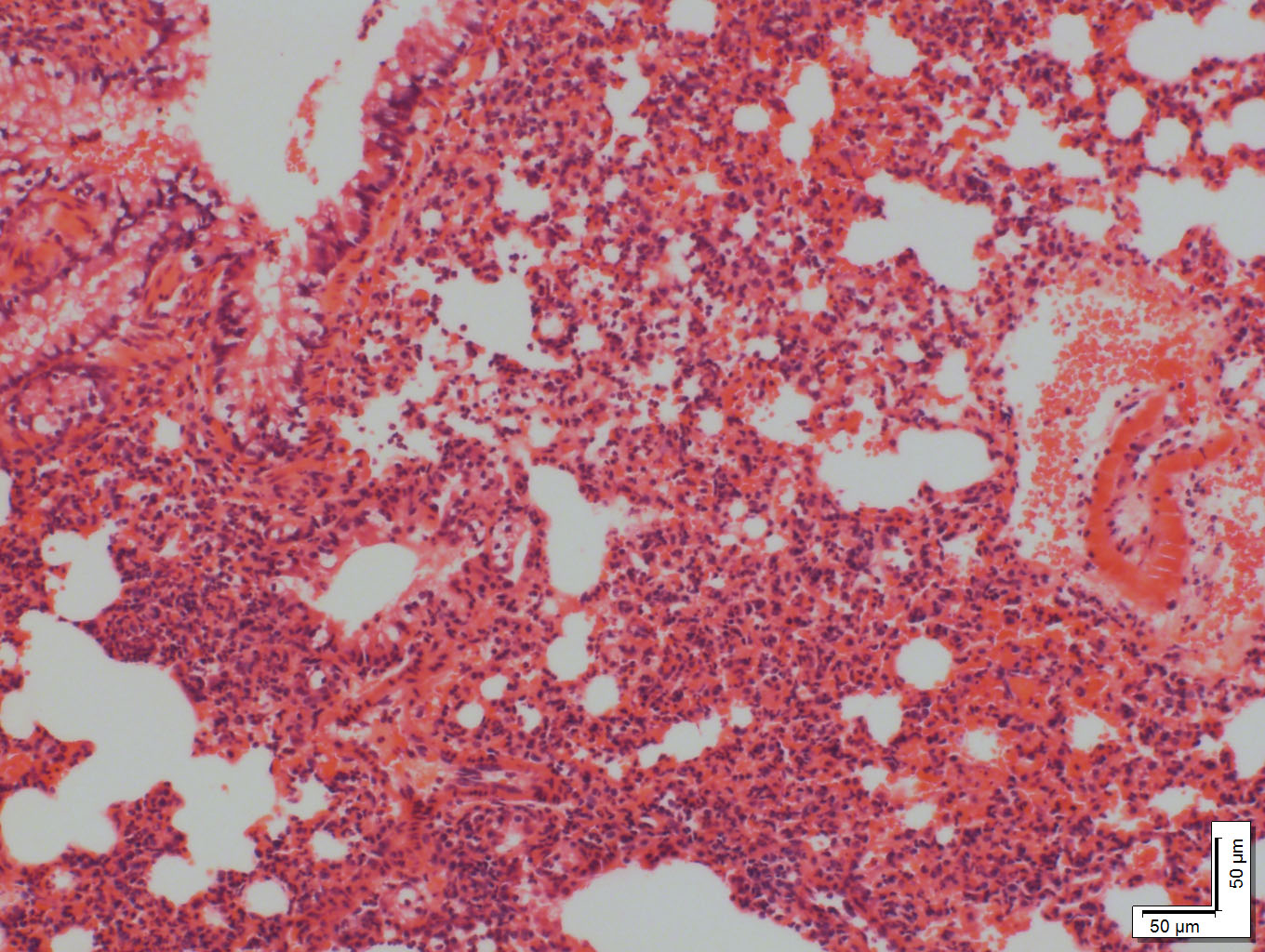

Supplement: Supplementary file 1 [file DataSheet1.ZIP › Original data/figure2-original data/HE/LPS.jpg]

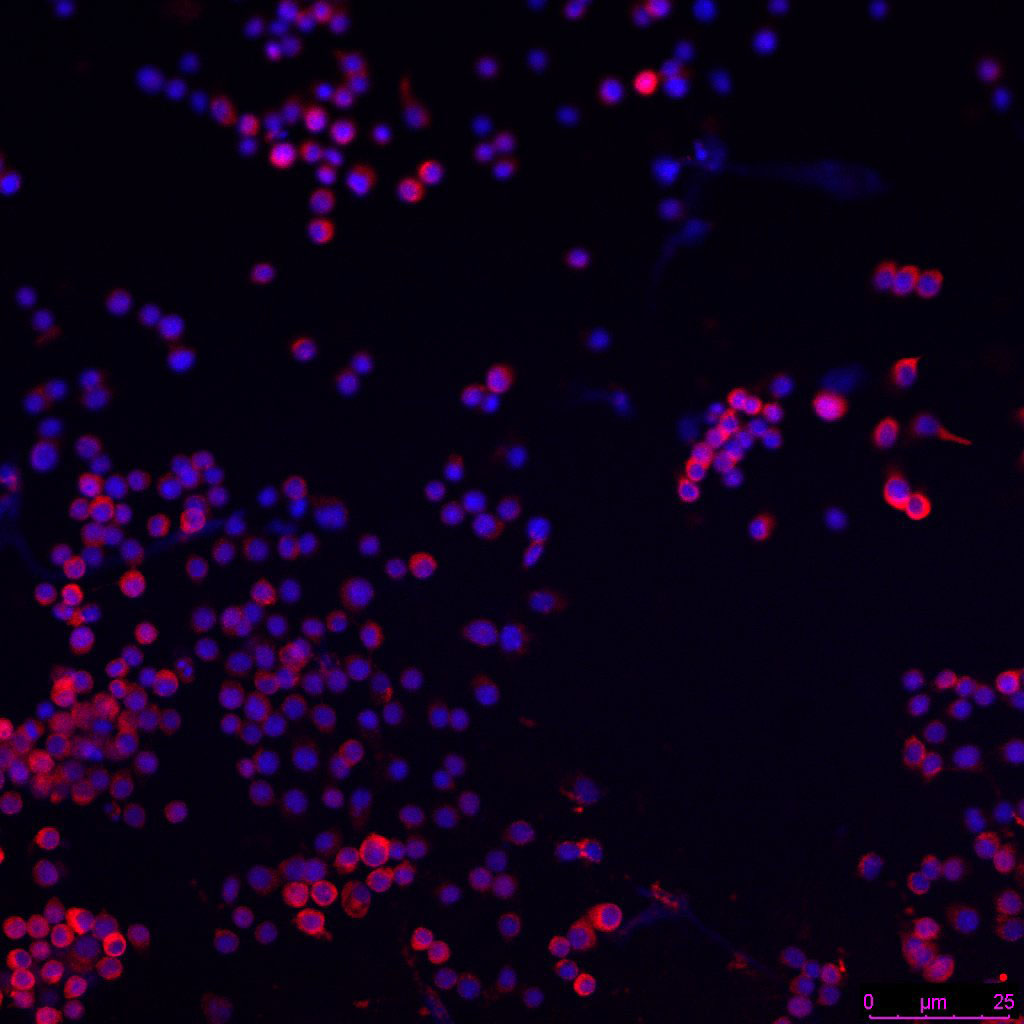

Supplement: Supplementary file 1 [file DataSheet1.ZIP › Original data/figure4-original data/IF-P65/BVA.jpg]

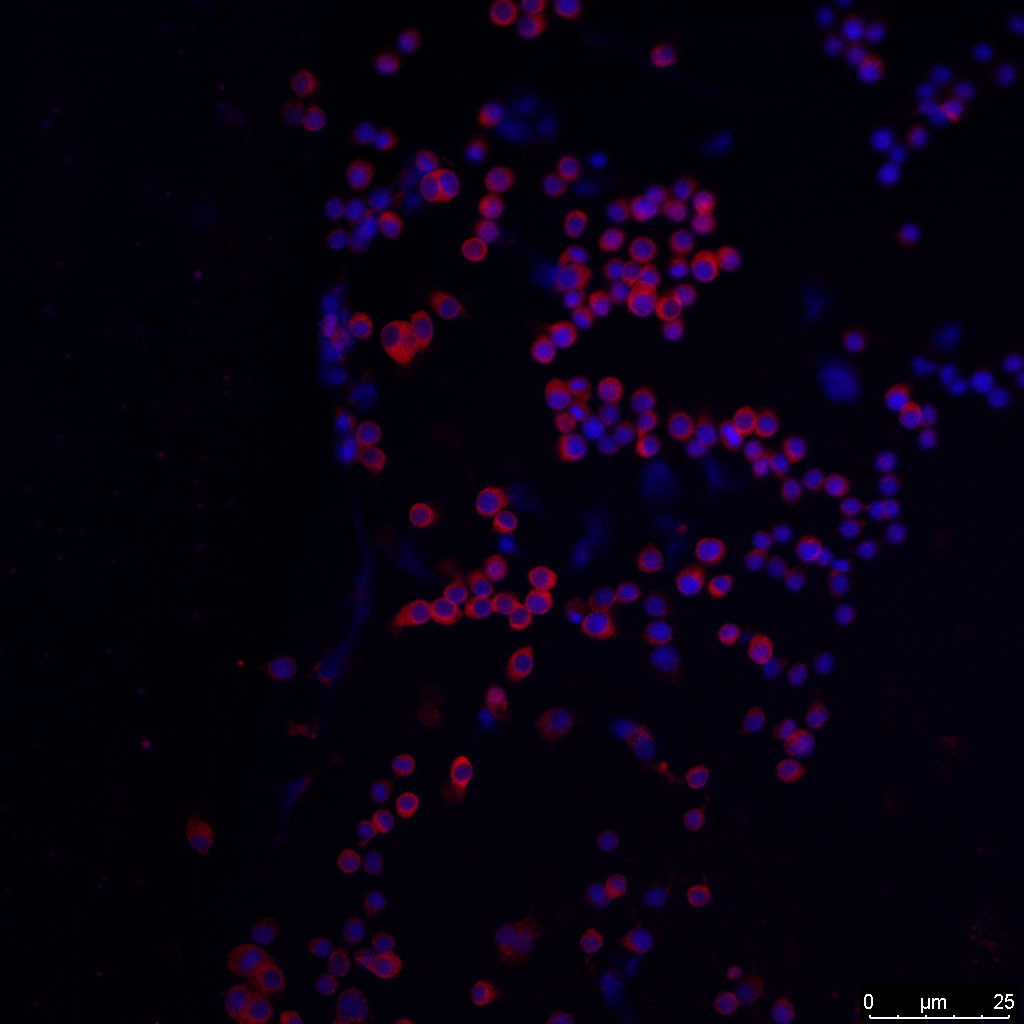

Supplement: Supplementary file 1 [file DataSheet1.ZIP › Original data/figure4-original data/IF-P65/CK.jpg]

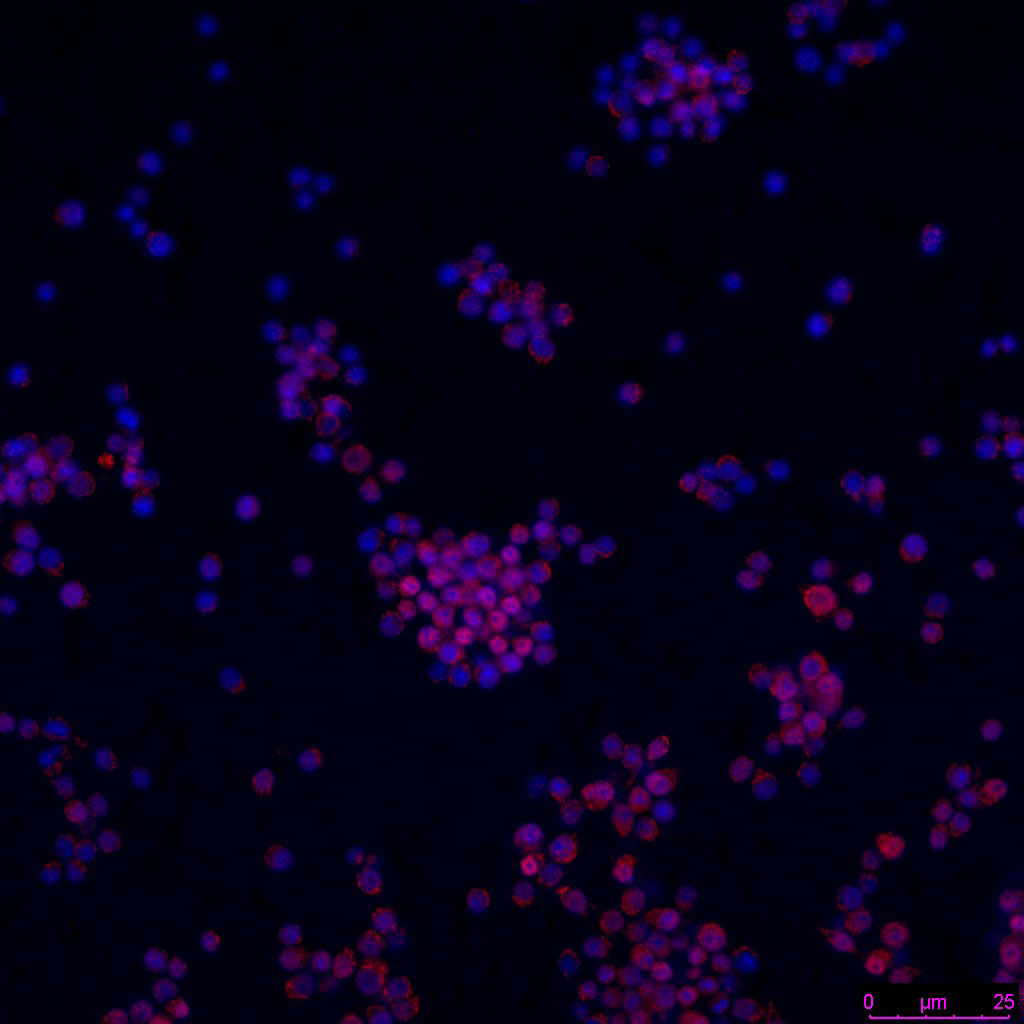

Supplement: Supplementary file 1 [file DataSheet1.ZIP › Original data/figure4-original data/IF-P65/LPS.jpg]

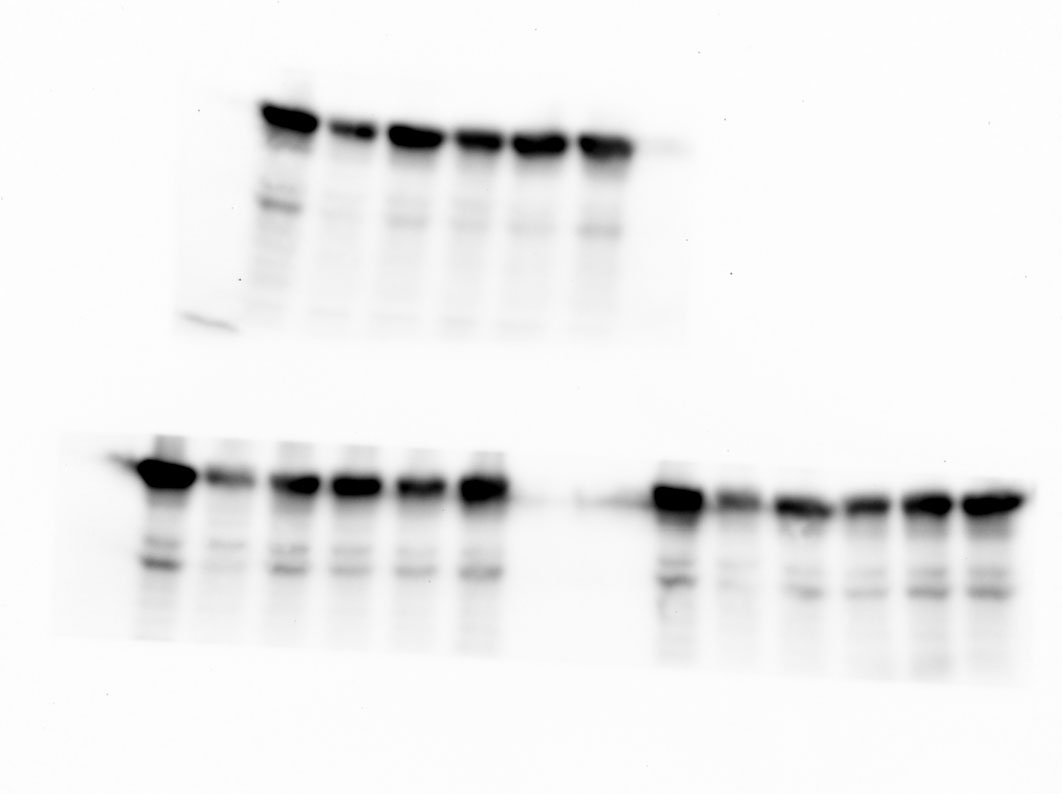

Supplement: Supplementary file 1 [file DataSheet1.ZIP › Original data/figure4-original data/IκBα+P-IκBα+LPS+IFNγ/IκBα+LPS+IFNγ-1,2,3.jpg]

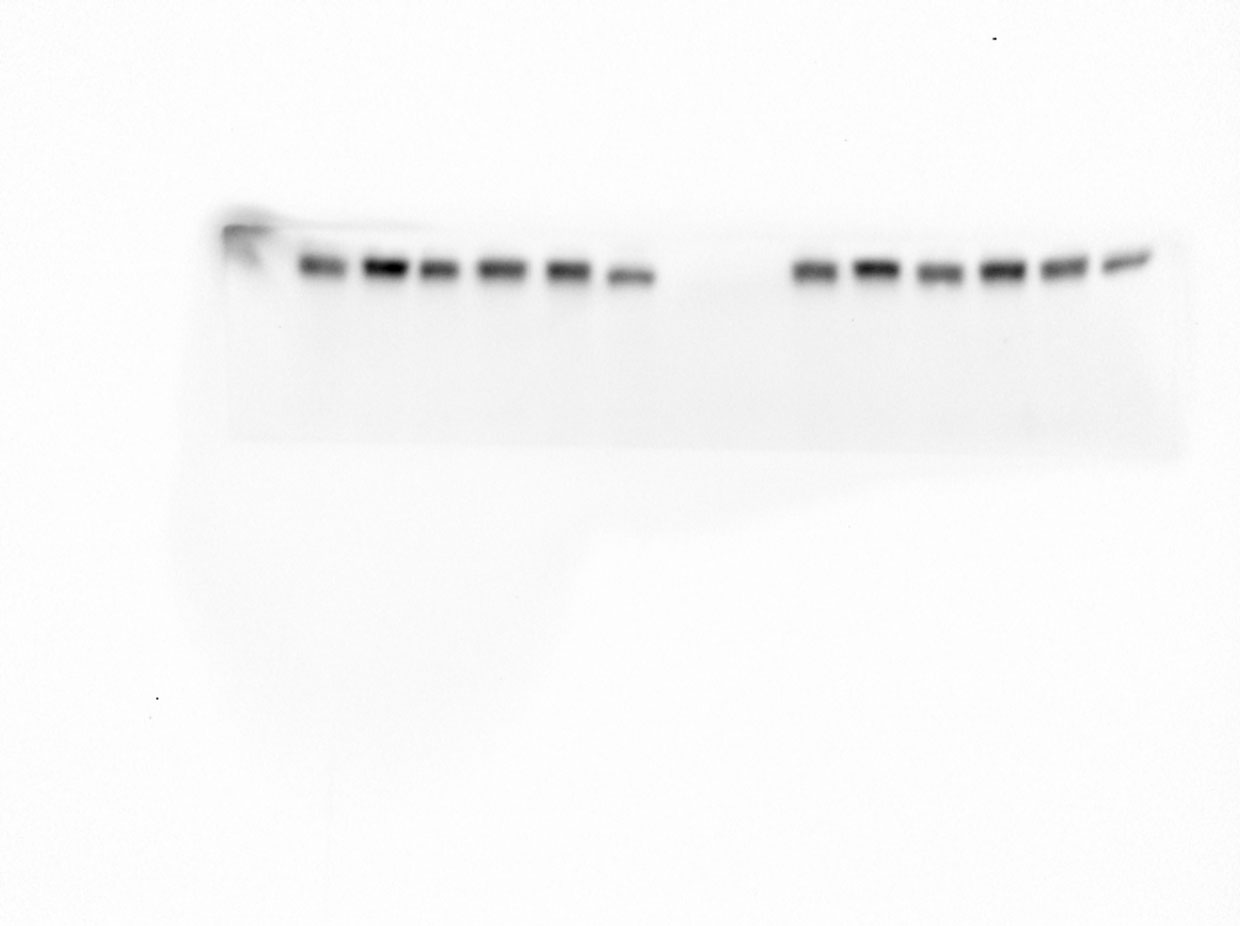

Supplement: Supplementary file 1 [file DataSheet1.ZIP › Original data/figure4-original data/IκBα+P-IκBα+LPS+IFNγ/P-IκBα+LPS+IFNγ-1,2.jpg]

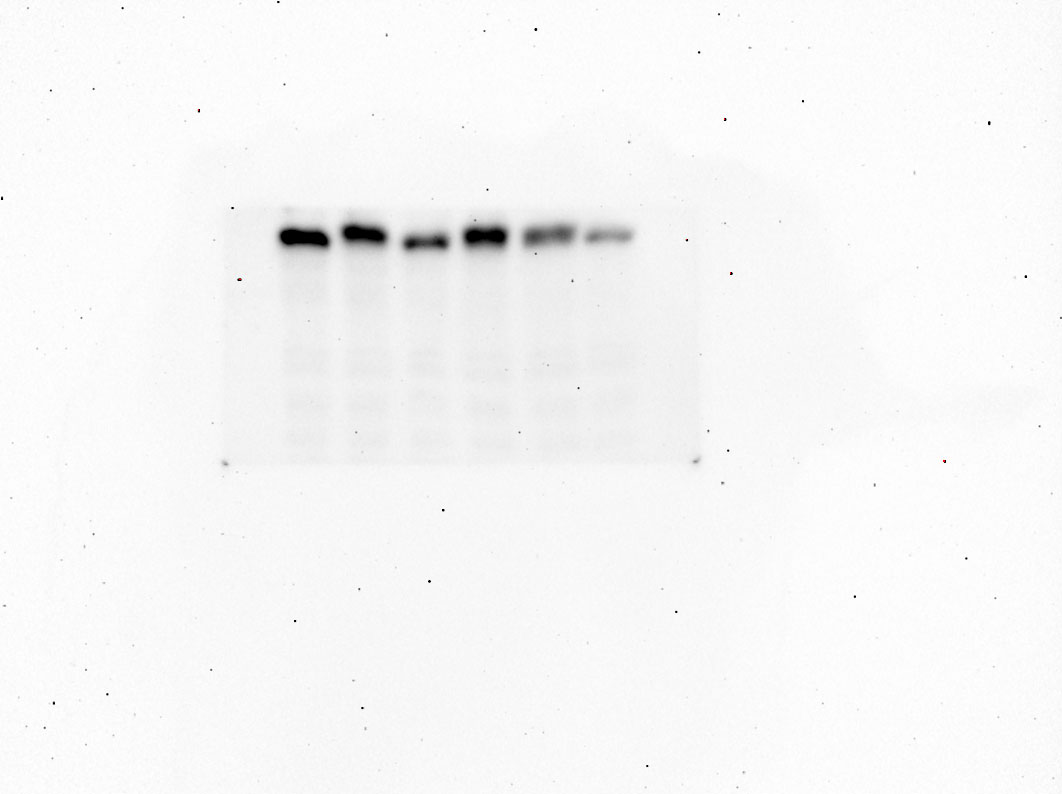

Supplement: Supplementary file 1 [file DataSheet1.ZIP › Original data/figure4-original data/IκBα+P-IκBα+LPS+IFNγ/P-IκBα+LPS+IFNγ-3.jpg]

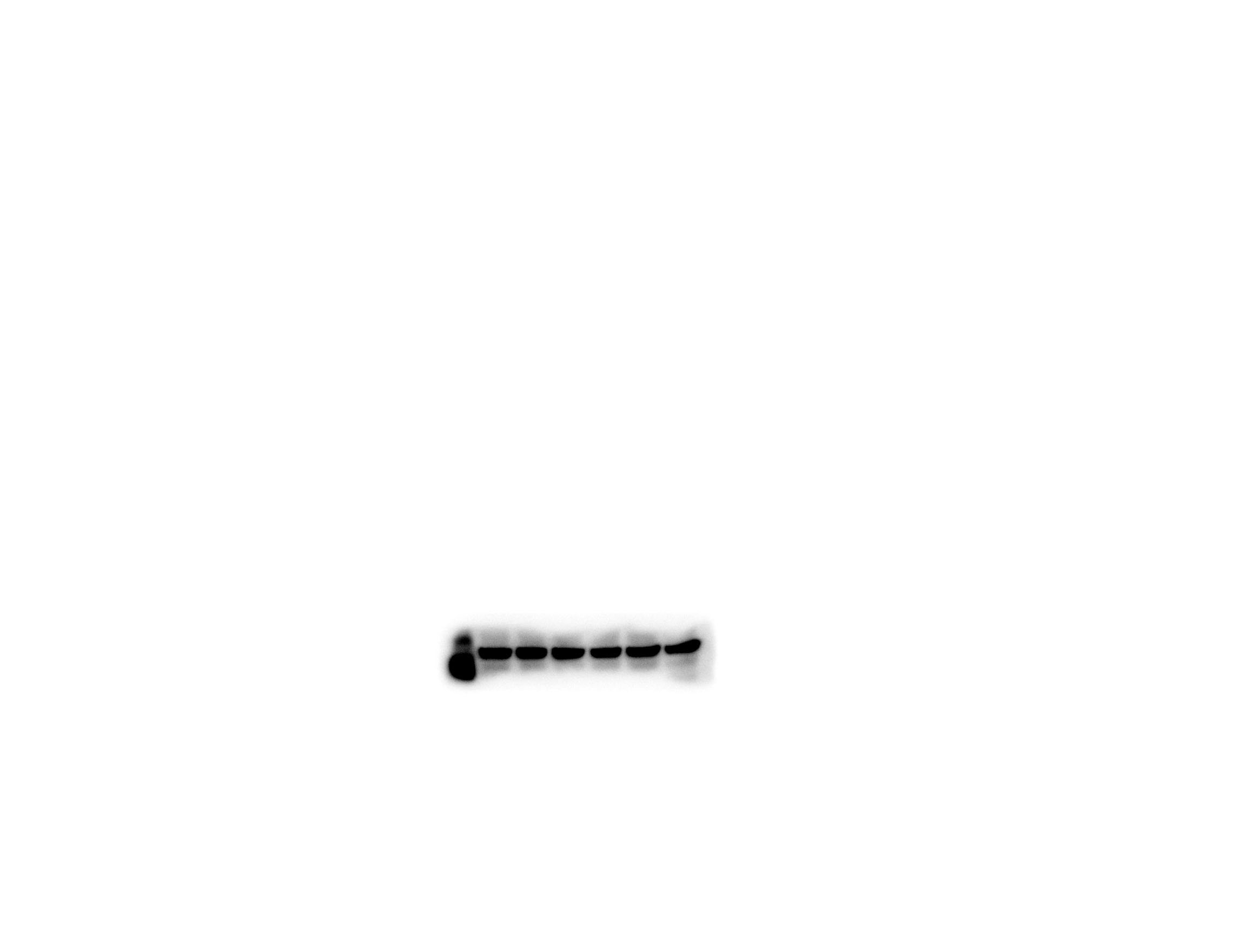

Supplement: Supplementary file 1 [file DataSheet1.ZIP › Original data/figure4-original data/IκBα+P-IκBα+LPS+IFNγ/Tubulin-IκBα+P-IκBα+LPS+IFNγ-1.jpg]

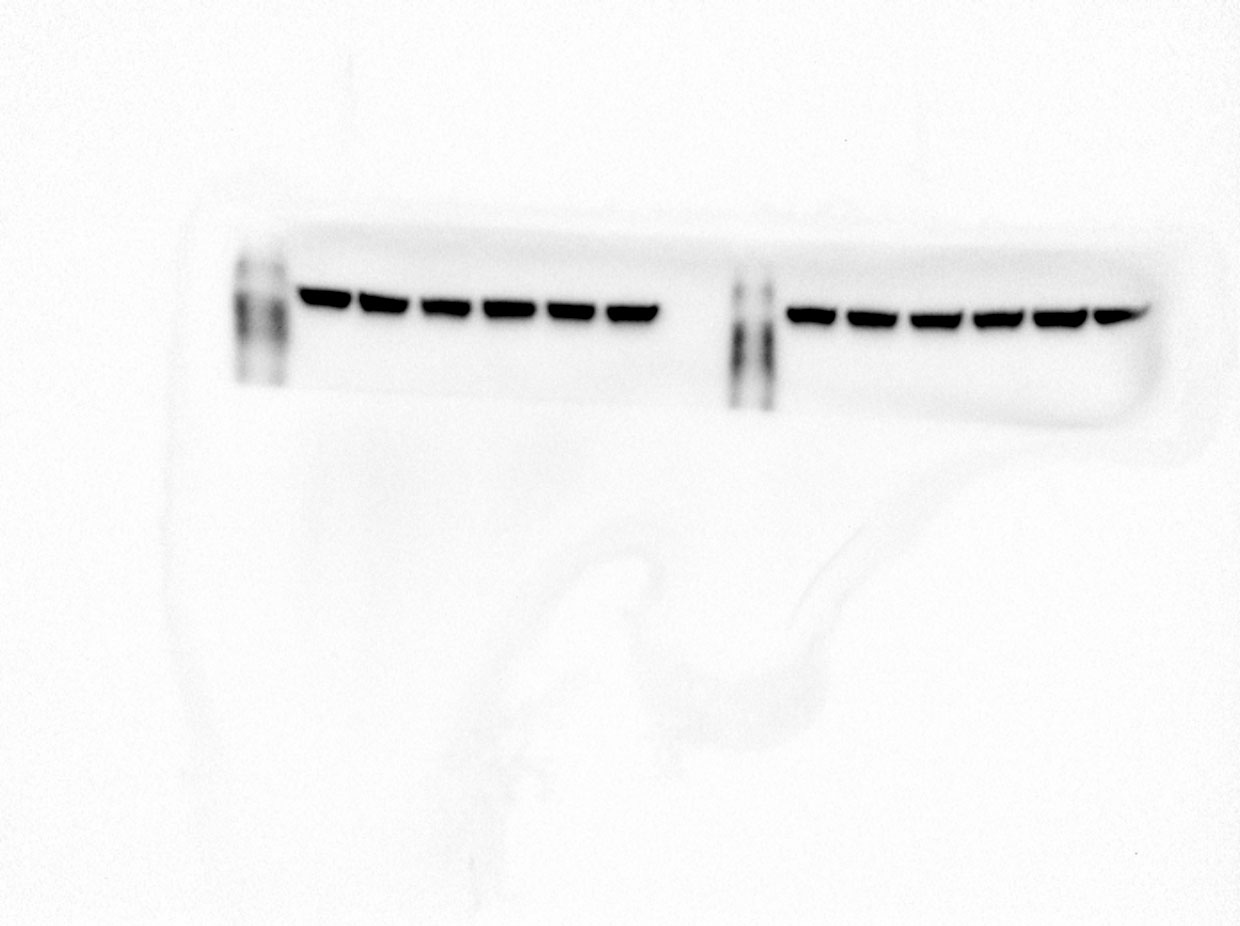

Supplement: Supplementary file 1 [file DataSheet1.ZIP › Original data/figure4-original data/IκBα+P-IκBα+LPS+IFNγ/Tubulin-IκBα+P-IκBα+LPS+IFNγ-2,3.jpg]

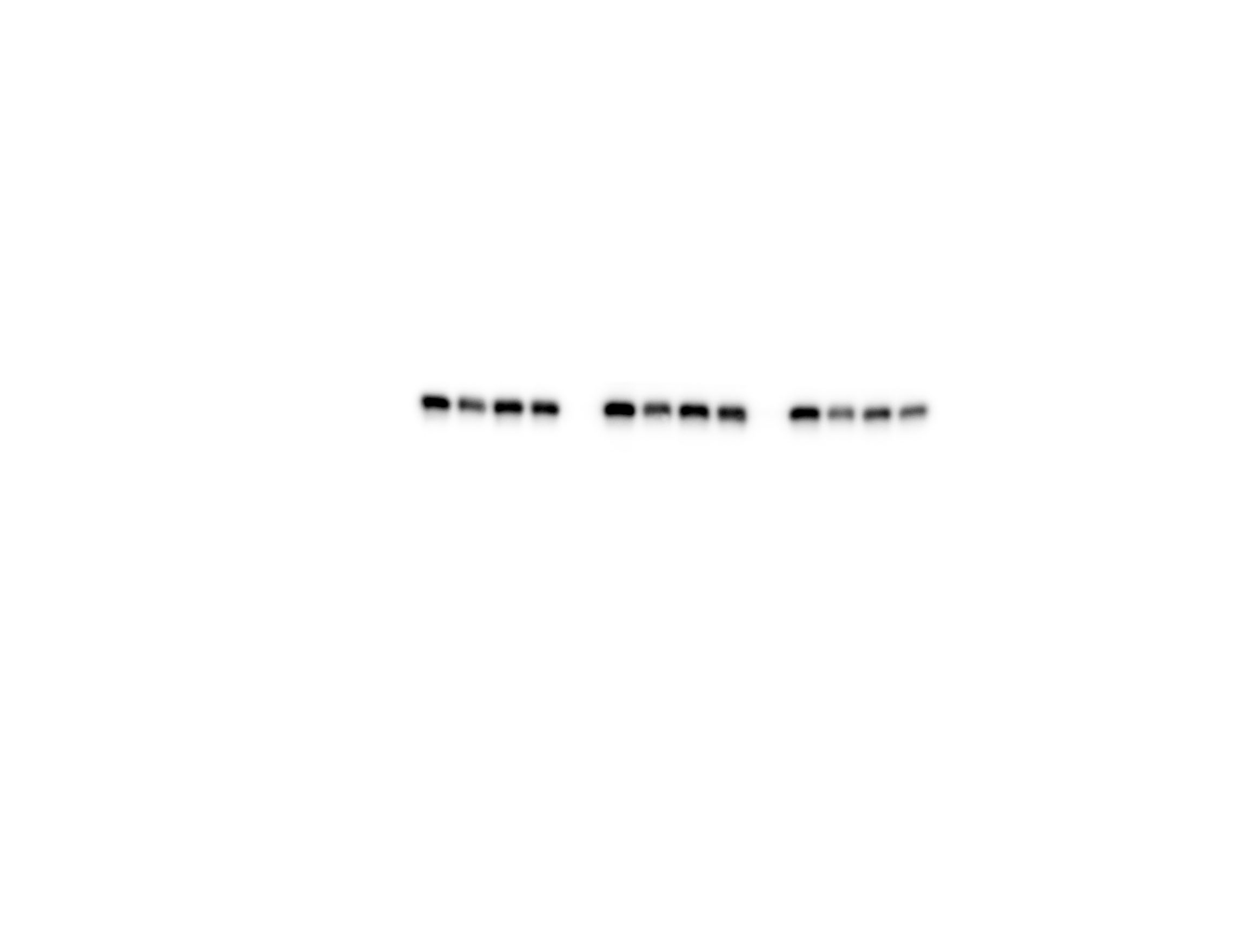

Supplement: Supplementary file 1 [file DataSheet1.ZIP › Original data/figure4-original data/IκBα+P-IκBα+TNFα+IFNγ/IκBα-TNFα+IFNγ-1,2,3.jpg]

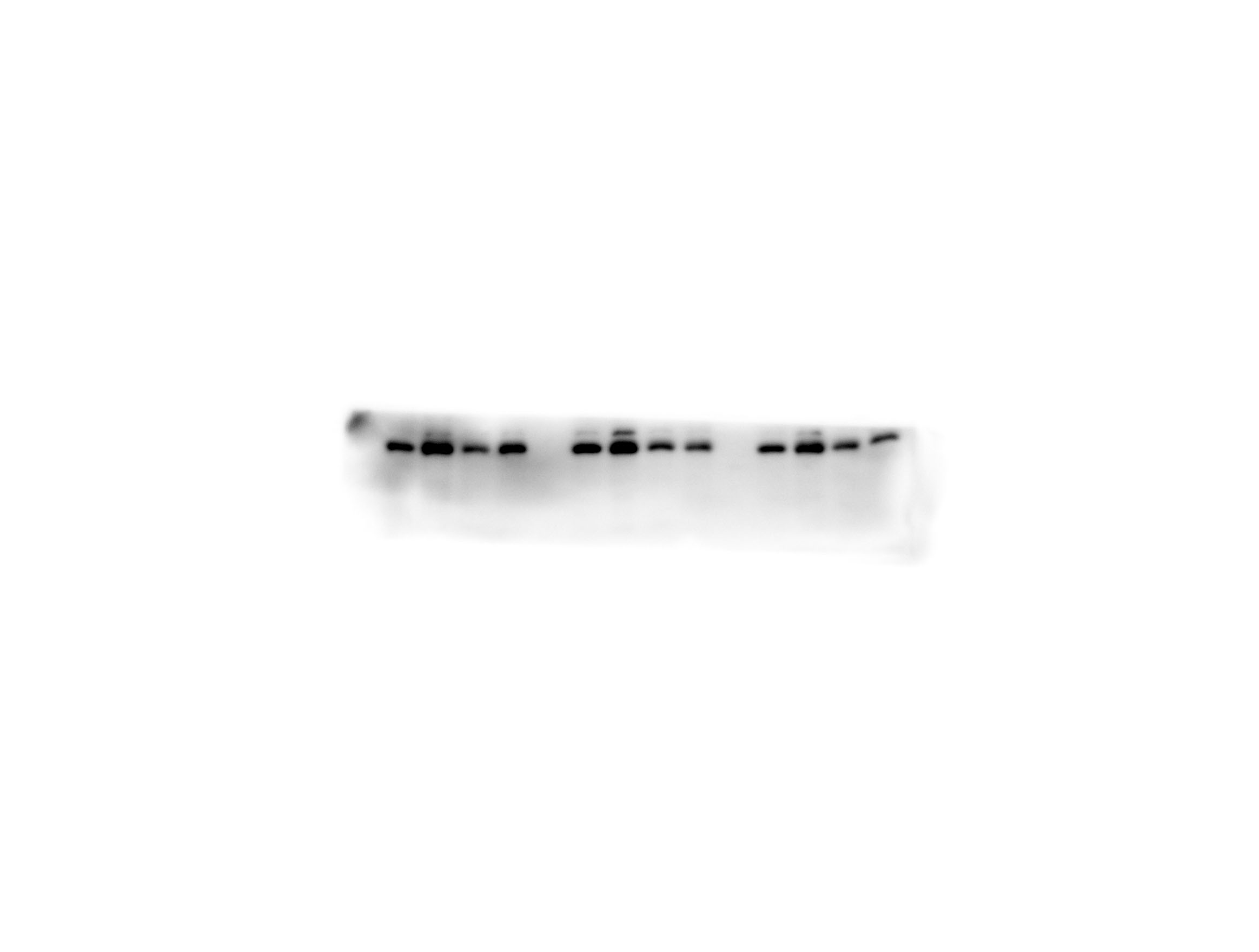

Supplement: Supplementary file 1 [file DataSheet1.ZIP › Original data/figure4-original data/IκBα+P-IκBα+TNFα+IFNγ/P-IκBα-TNFα+IFNγ-1,2,3.jpg]

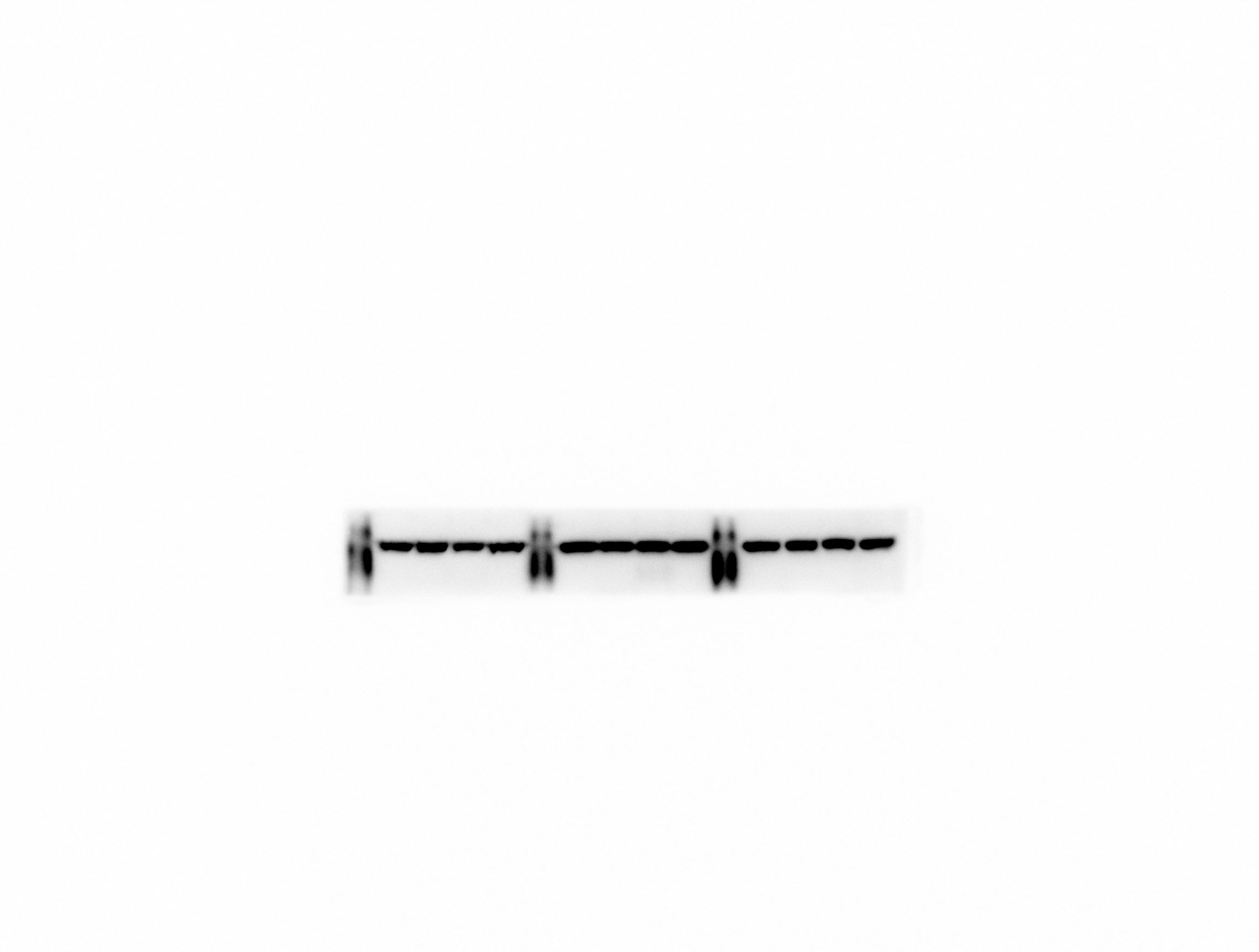

Supplement: Supplementary file 1 [file DataSheet1.ZIP › Original data/figure4-original data/IκBα+P-IκBα+TNFα+IFNγ/Tubulin--TNFα+IFNγ-1,2,3.jpg]

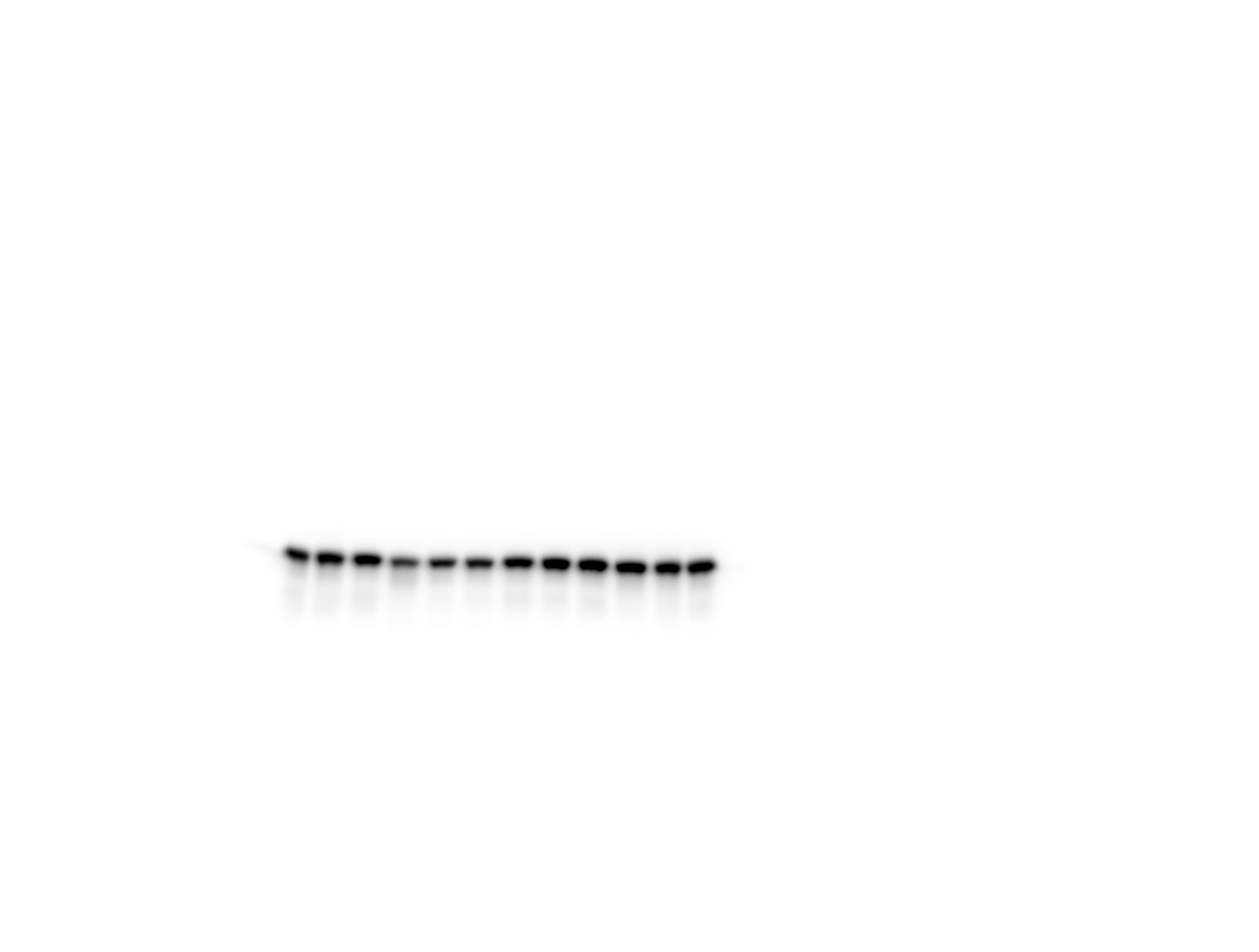

Supplement: Supplementary file 1 [file DataSheet1.ZIP › Original data/figure4-original data/mice-IκBα/IκBα-BVA-Mice-Lung-1,2,3.jpg]

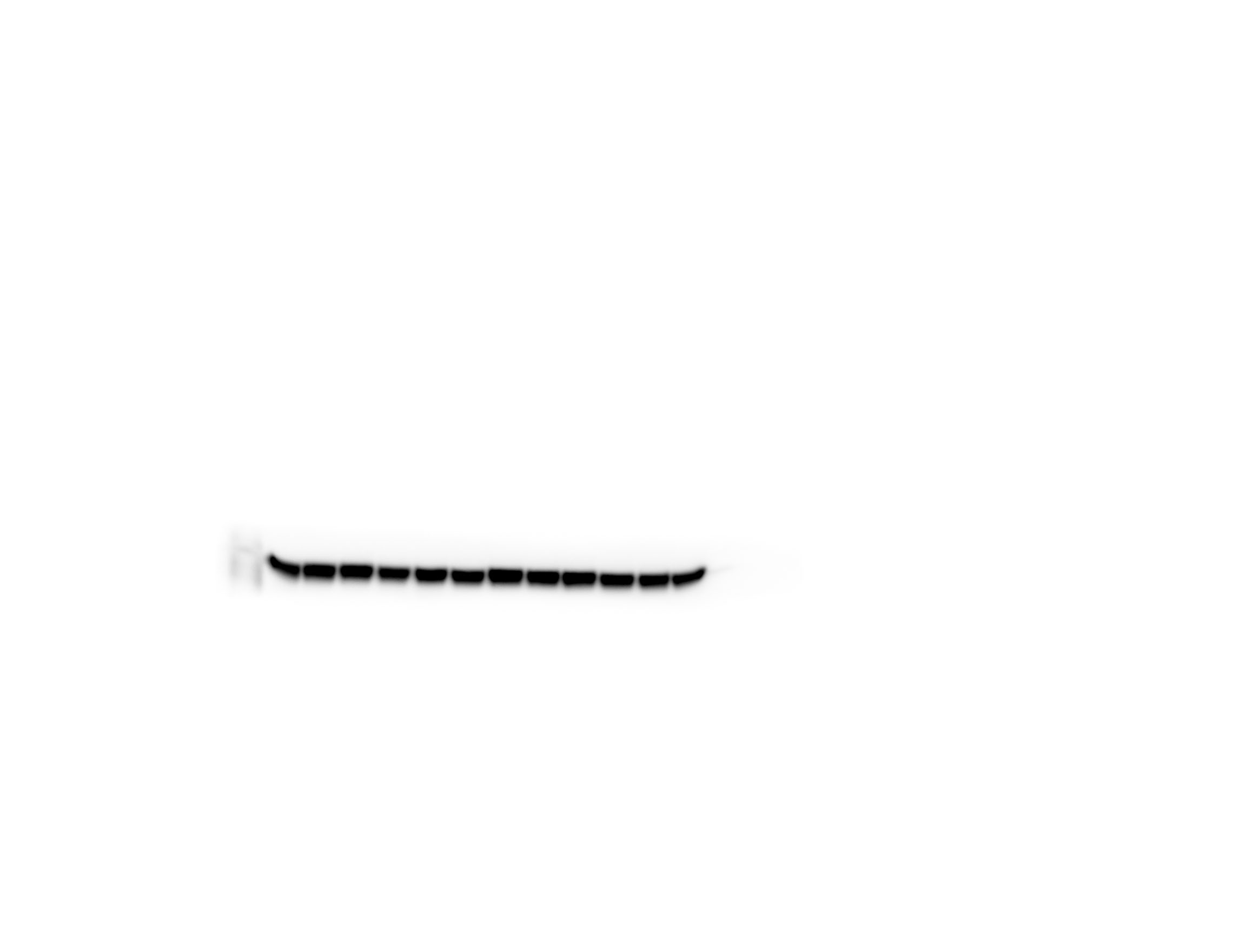

Supplement: Supplementary file 1 [file DataSheet1.ZIP › Original data/figure4-original data/mice-IκBα/Tubulin-BVA-Mice-Lung-1,2,3.jpg]

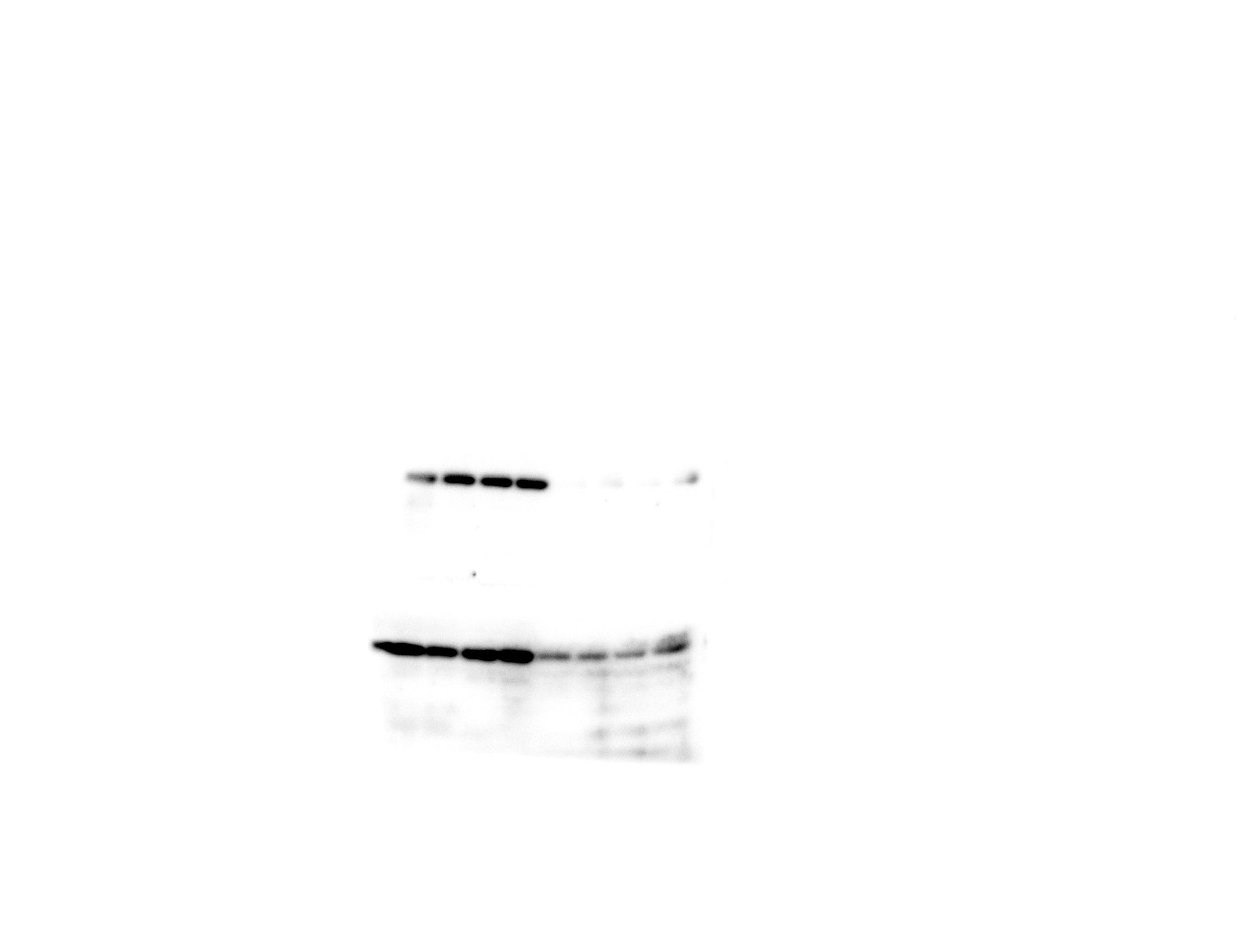

Supplement: Supplementary file 1 [file DataSheet1.ZIP › Original data/figure4-original data/P65-C-N/GAPDH-C-N-1,2.jpg]

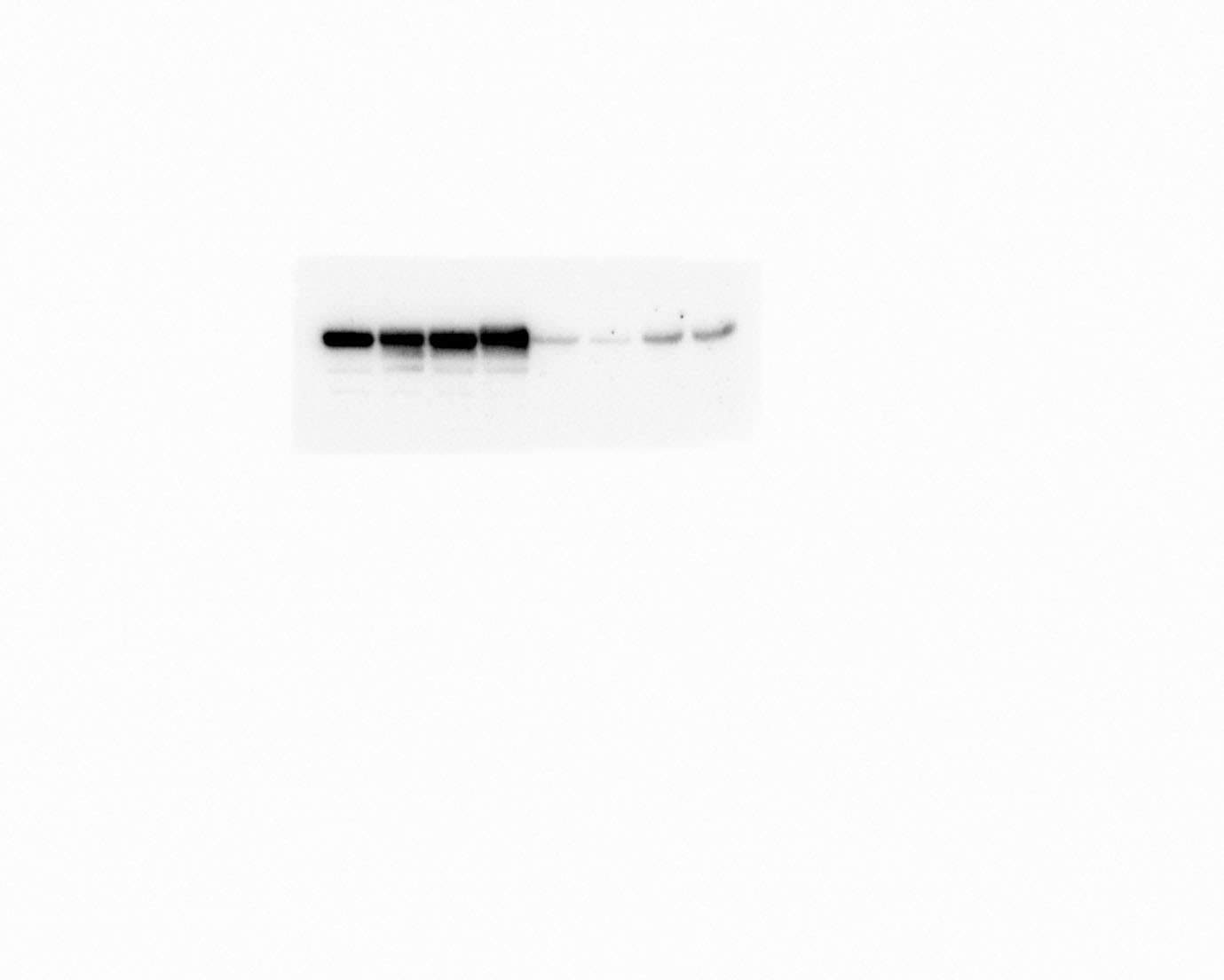

Supplement: Supplementary file 1 [file DataSheet1.ZIP › Original data/figure4-original data/P65-C-N/GAPDH-C-N-3.jpg]

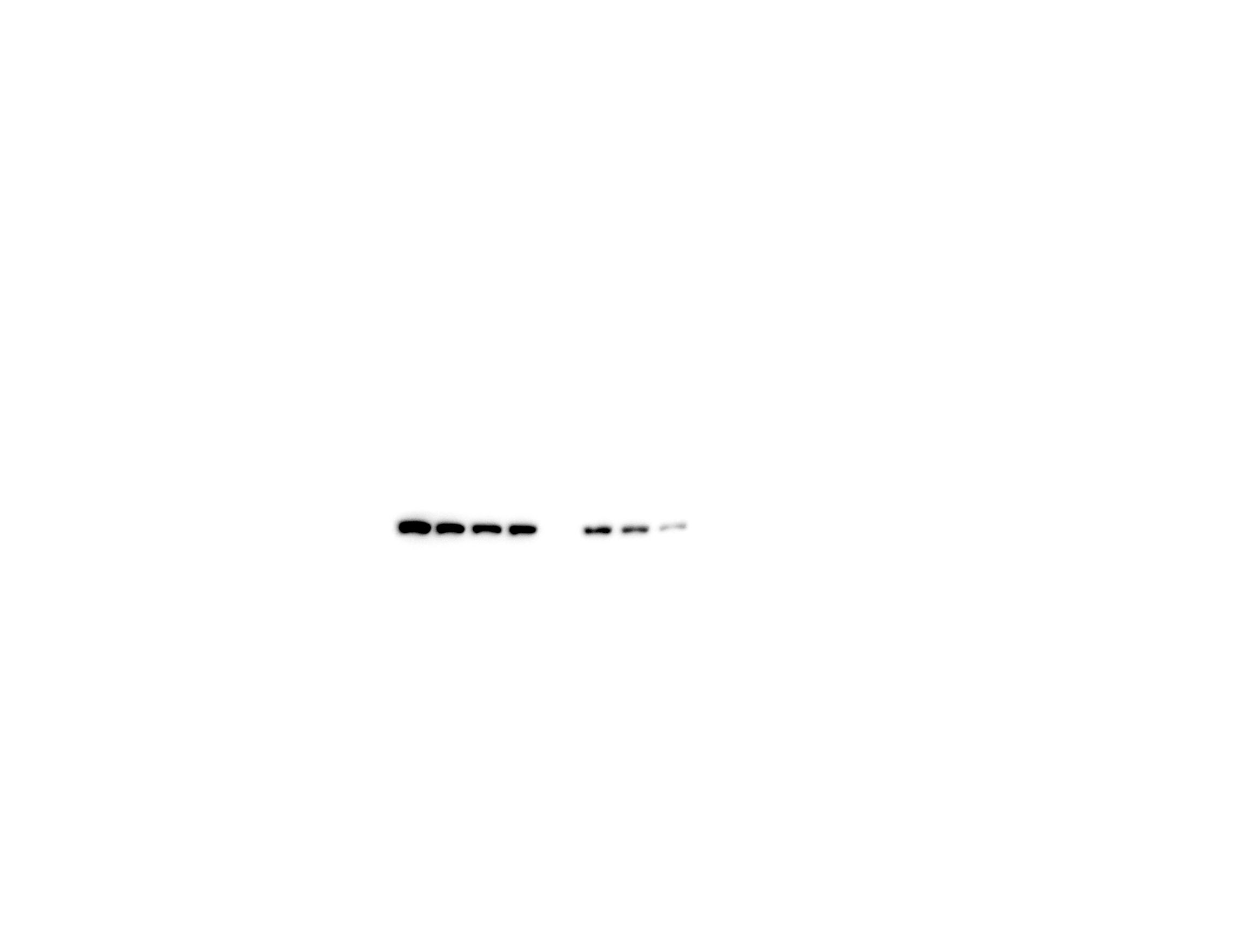

Supplement: Supplementary file 1 [file DataSheet1.ZIP › Original data/figure4-original data/P65-C-N/P65-C-N-1.jpg]

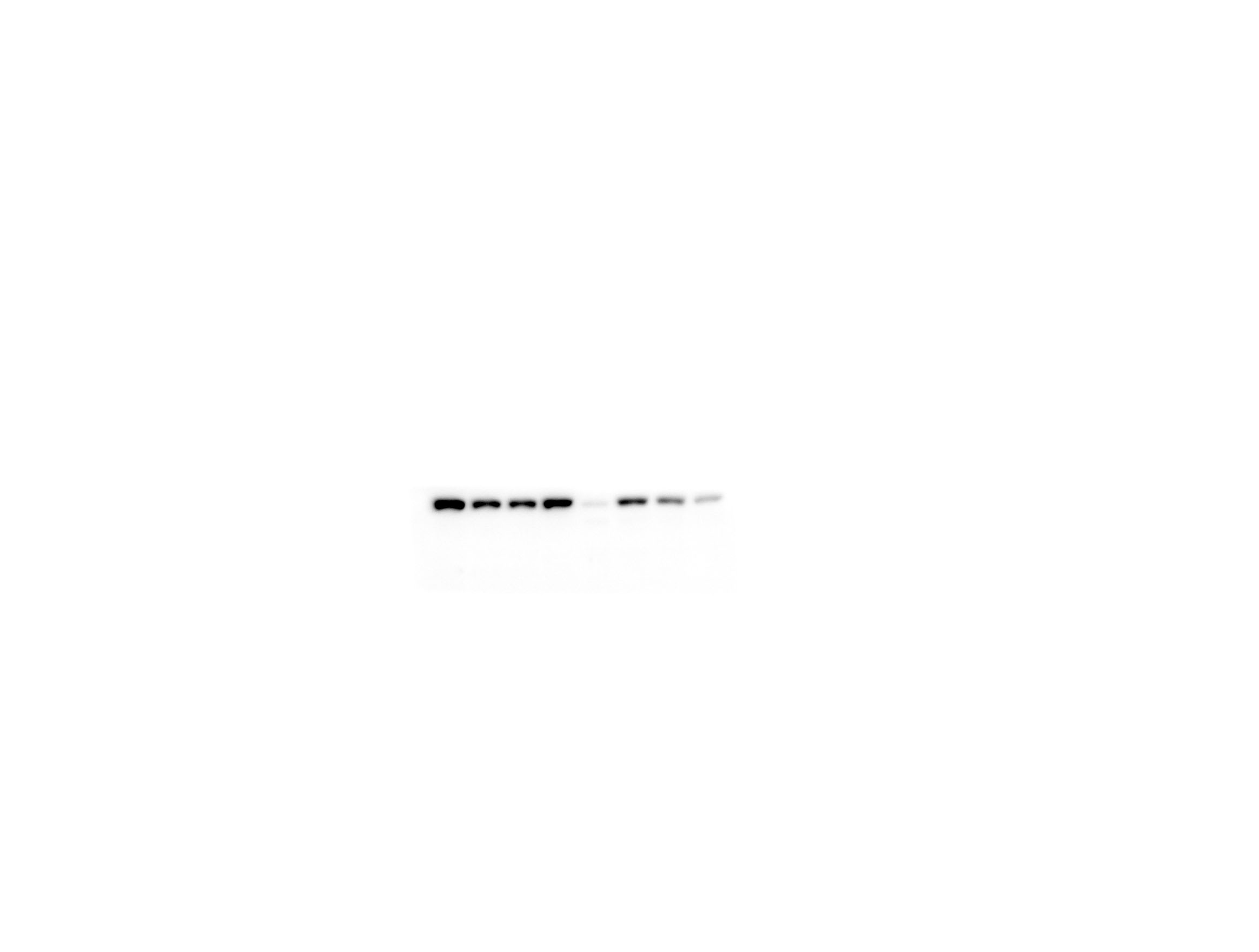

Supplement: Supplementary file 1 [file DataSheet1.ZIP › Original data/figure4-original data/P65-C-N/P65-C-N-2.jpg]

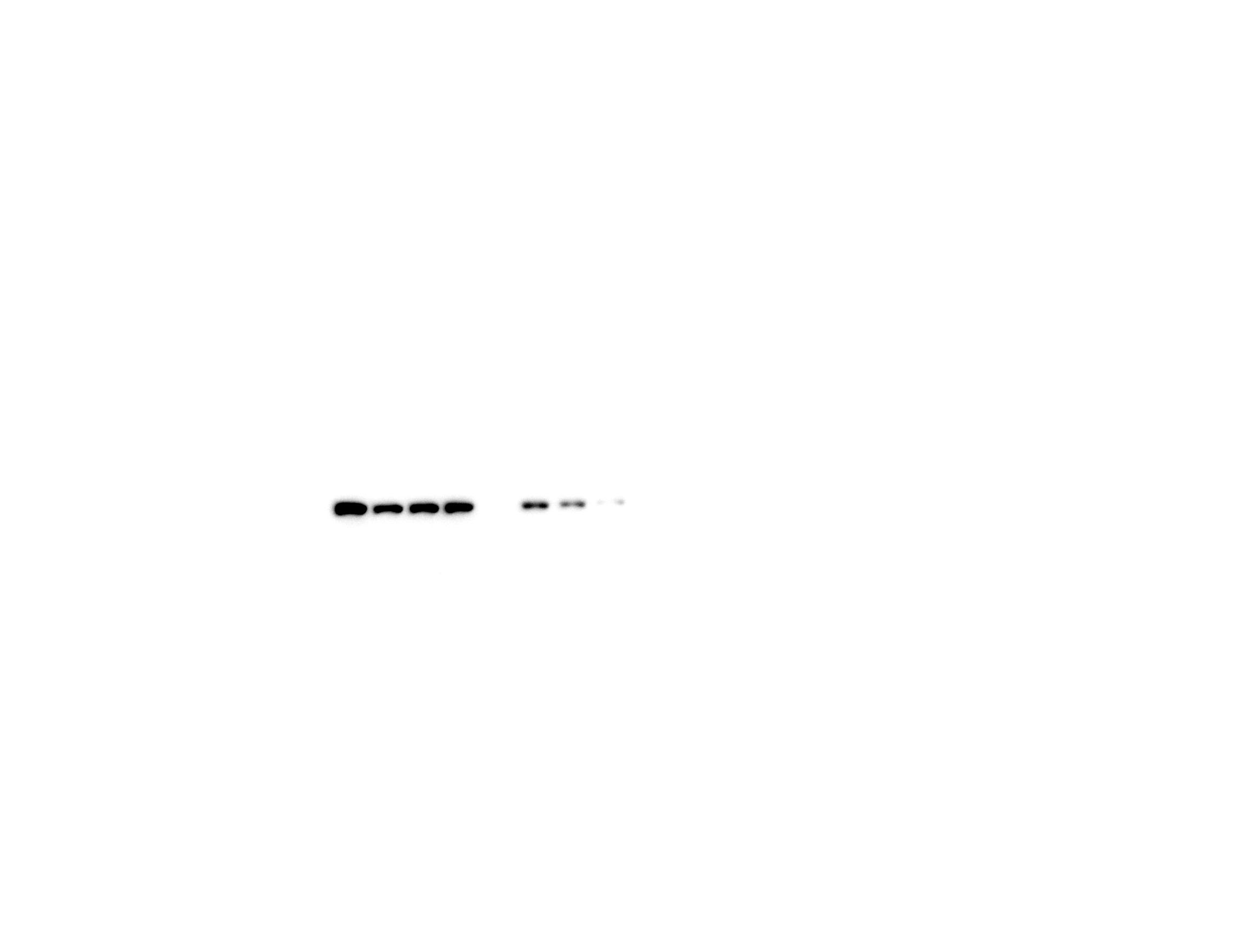

Supplement: Supplementary file 1 [file DataSheet1.ZIP › Original data/figure4-original data/P65-C-N/P65-C-N-3.jpg]

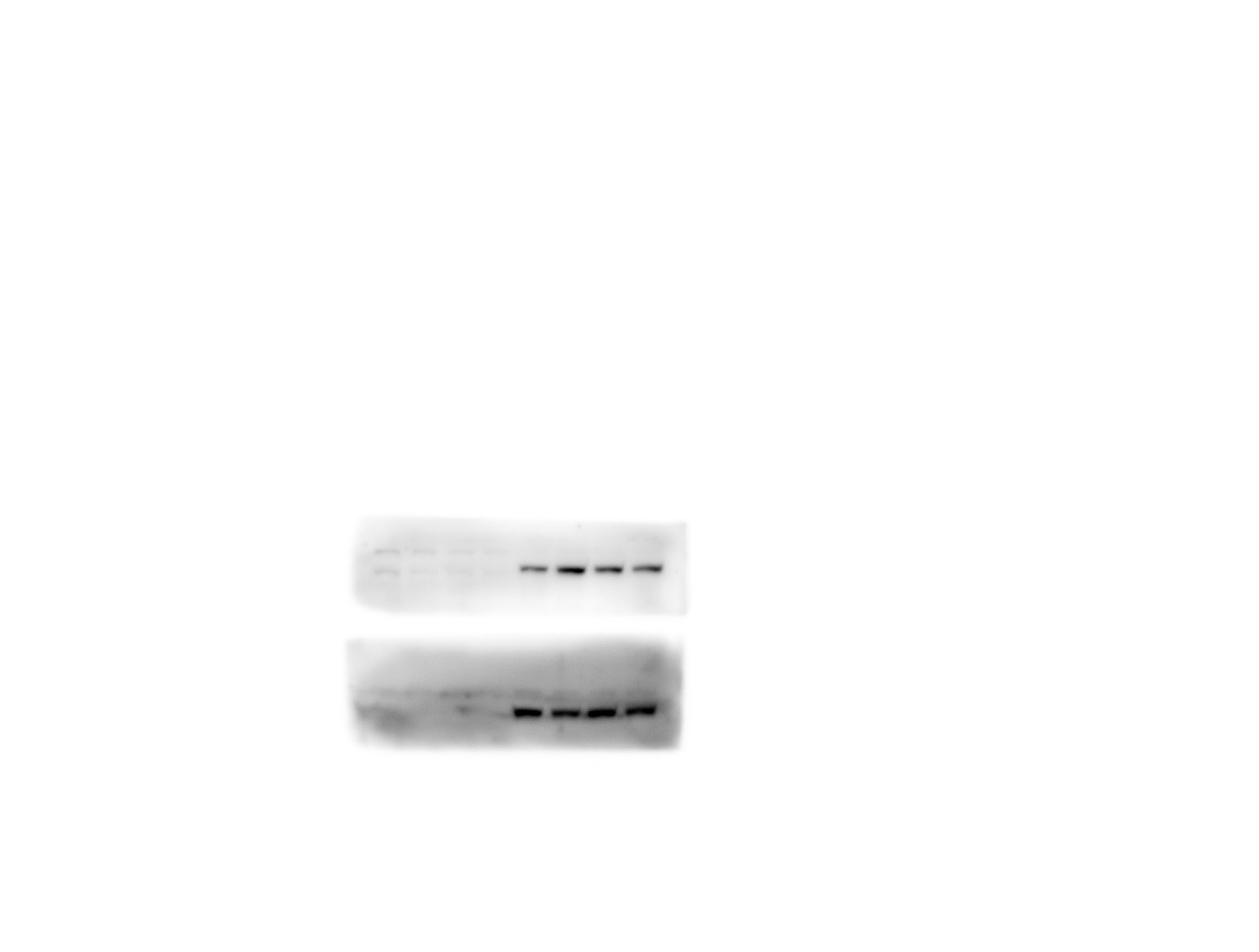

Supplement: Supplementary file 1 [file DataSheet1.ZIP › Original data/figure4-original data/P65-C-N/PARP1-C-N-1,2.jpg]

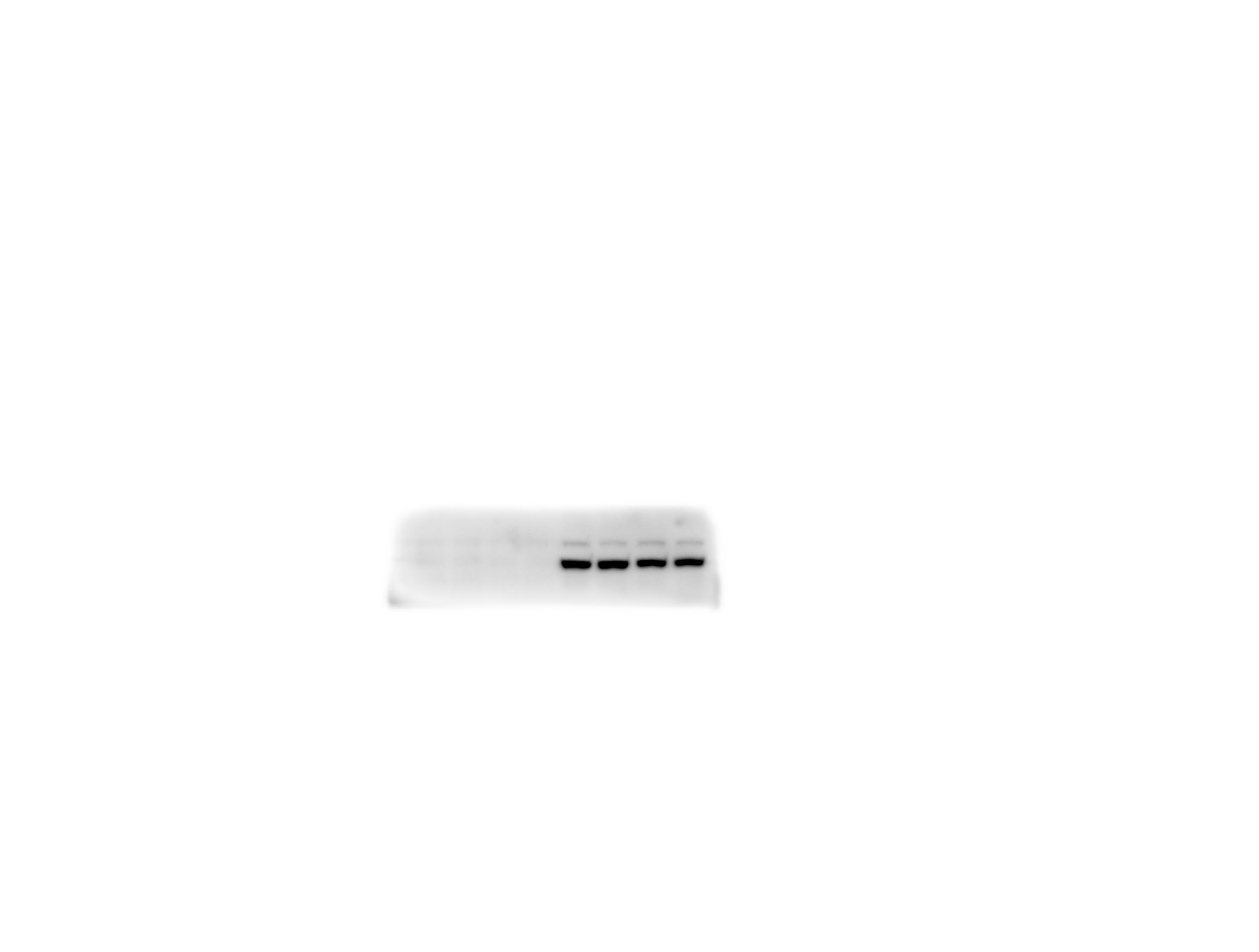

Supplement: Supplementary file 1 [file DataSheet1.ZIP › Original data/figure4-original data/P65-C-N/PARP1-C-N-3.jpg]

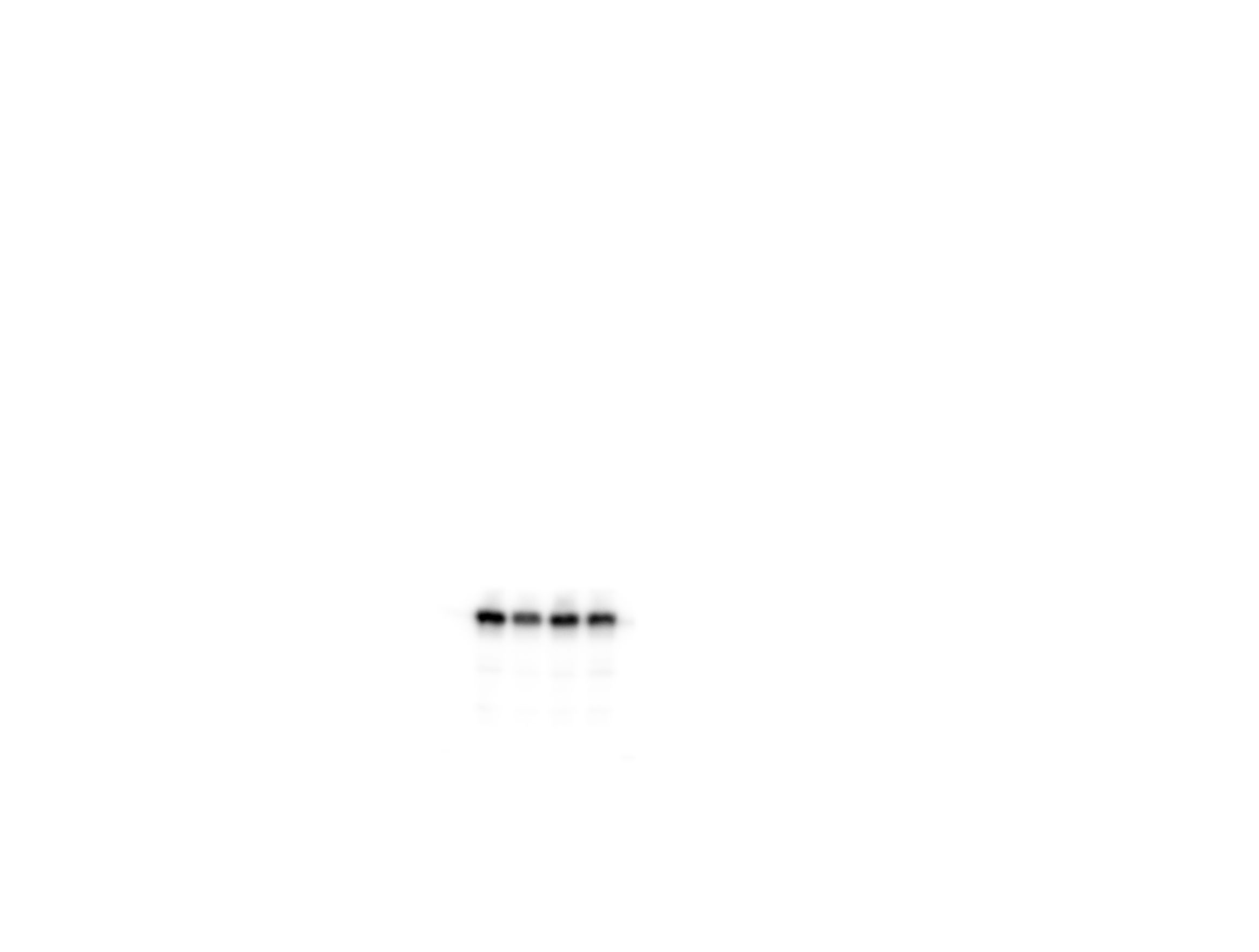

Supplement: Supplementary file 1 [file DataSheet1.ZIP › Original data/figure5-original data/DTT/IκBα-DTT-1.jpg]

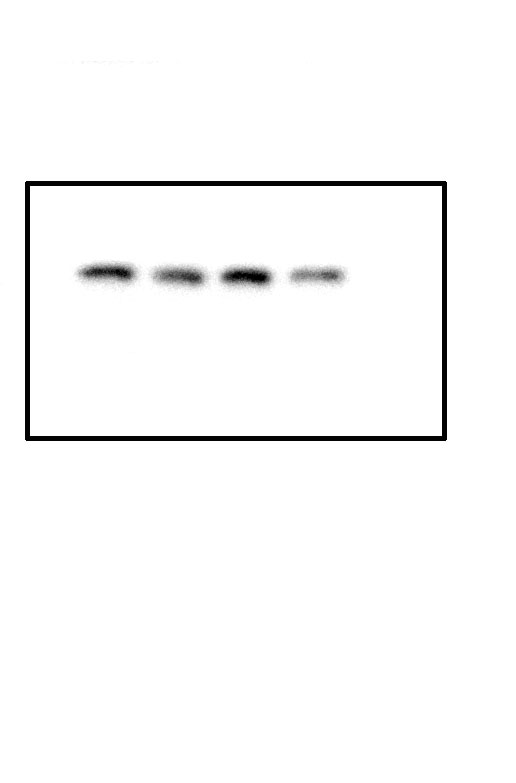

Supplement: Supplementary file 1 [file DataSheet1.ZIP › Original data/figure5-original data/DTT/IκBα-DTT-2.jpg]

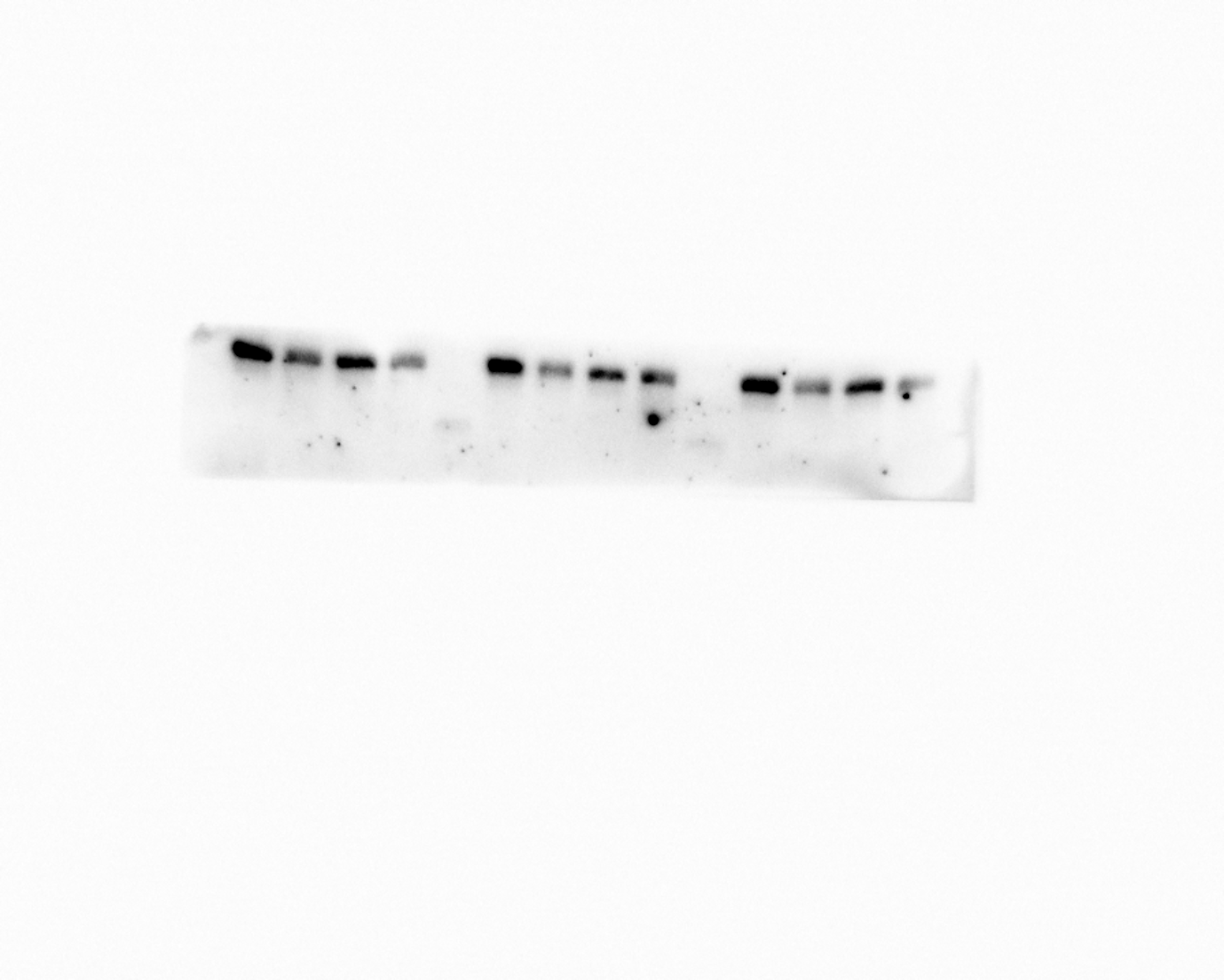

Supplement: Supplementary file 1 [file DataSheet1.ZIP › Original data/figure5-original data/DTT/IκBα-DTT-3.tif]

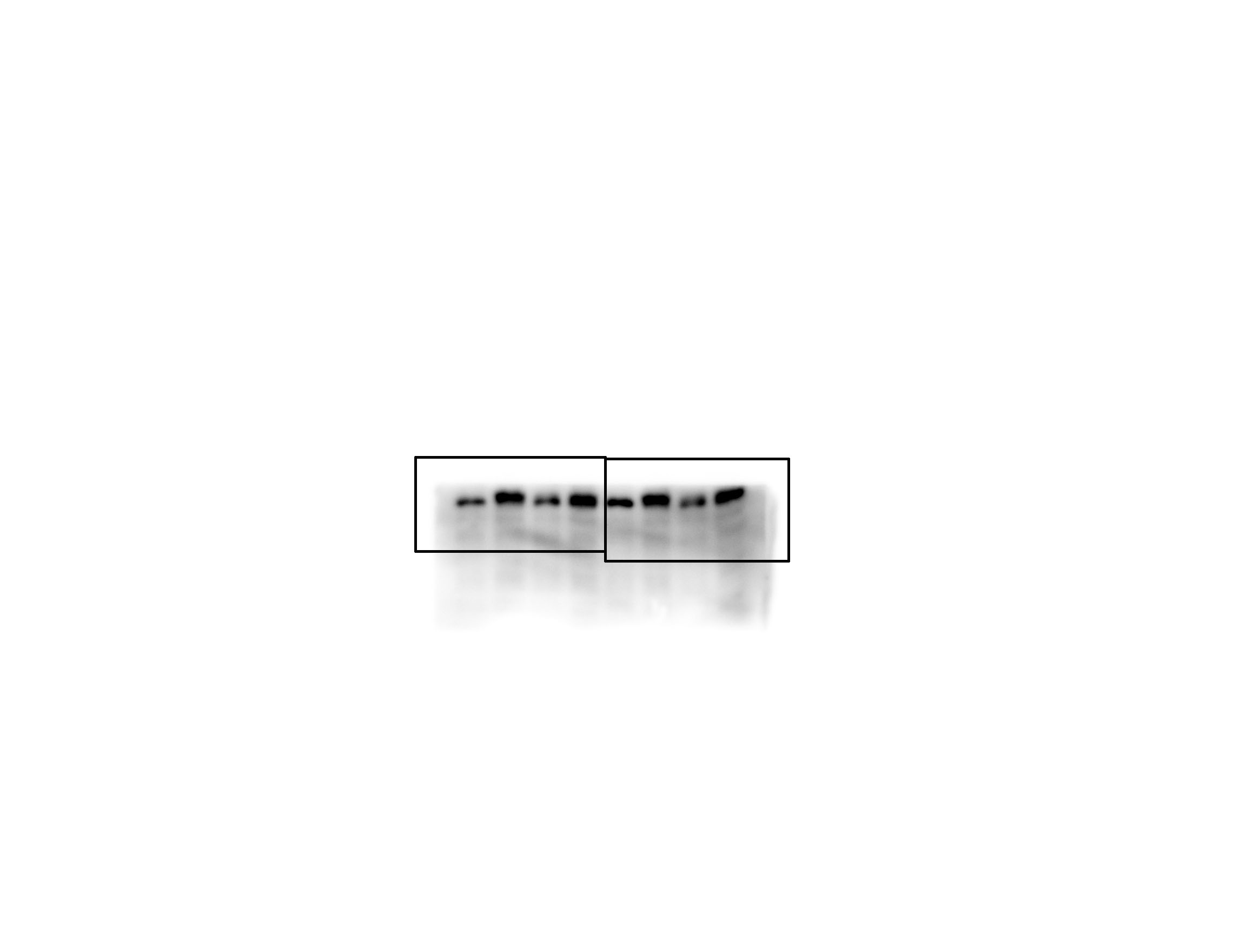

Supplement: Supplementary file 1 [file DataSheet1.ZIP › Original data/figure5-original data/DTT/P-IκBα-DTT-1,2.jpg]

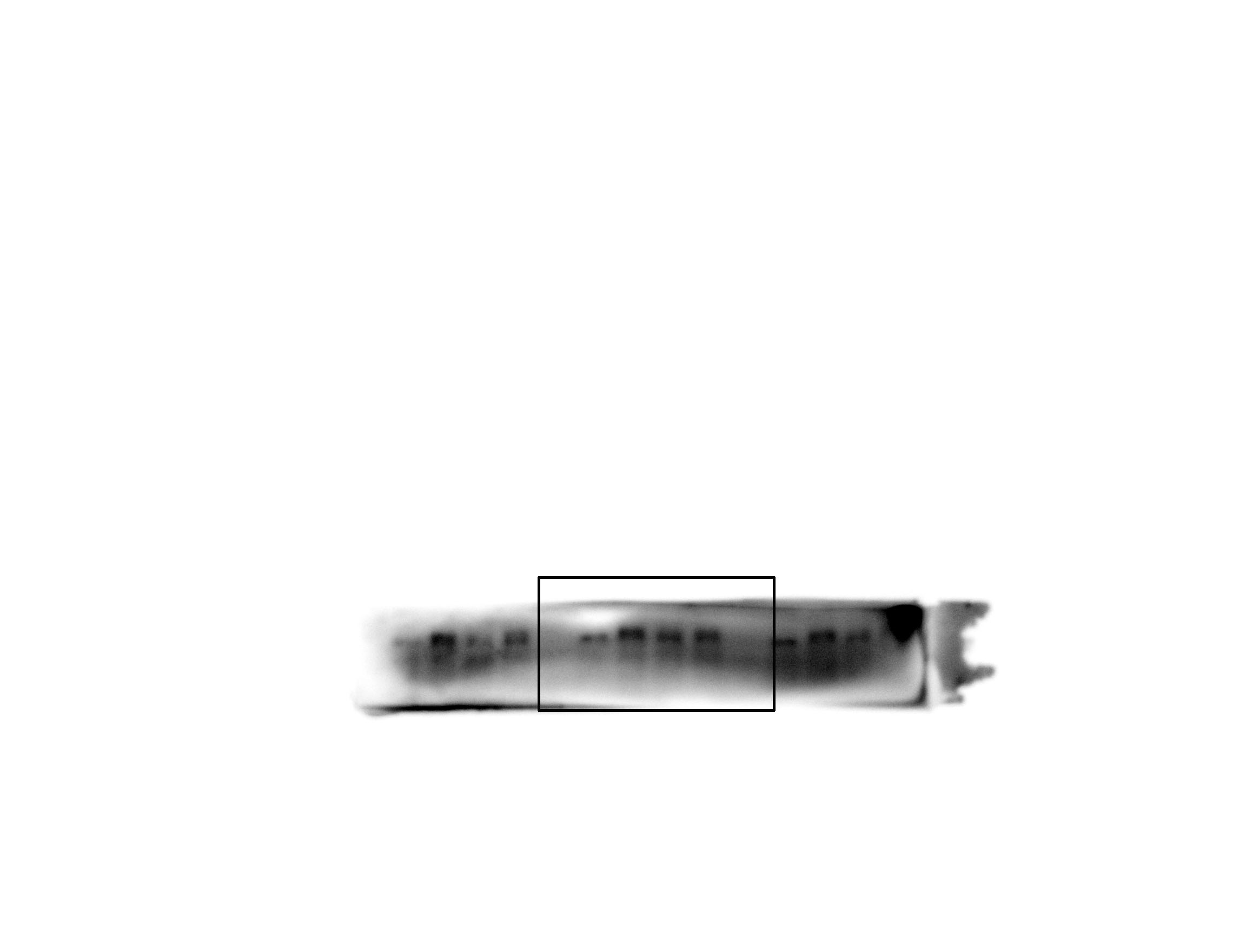

Supplement: Supplementary file 1 [file DataSheet1.ZIP › Original data/figure5-original data/DTT/P-IκBα-DTT-3.jpg]

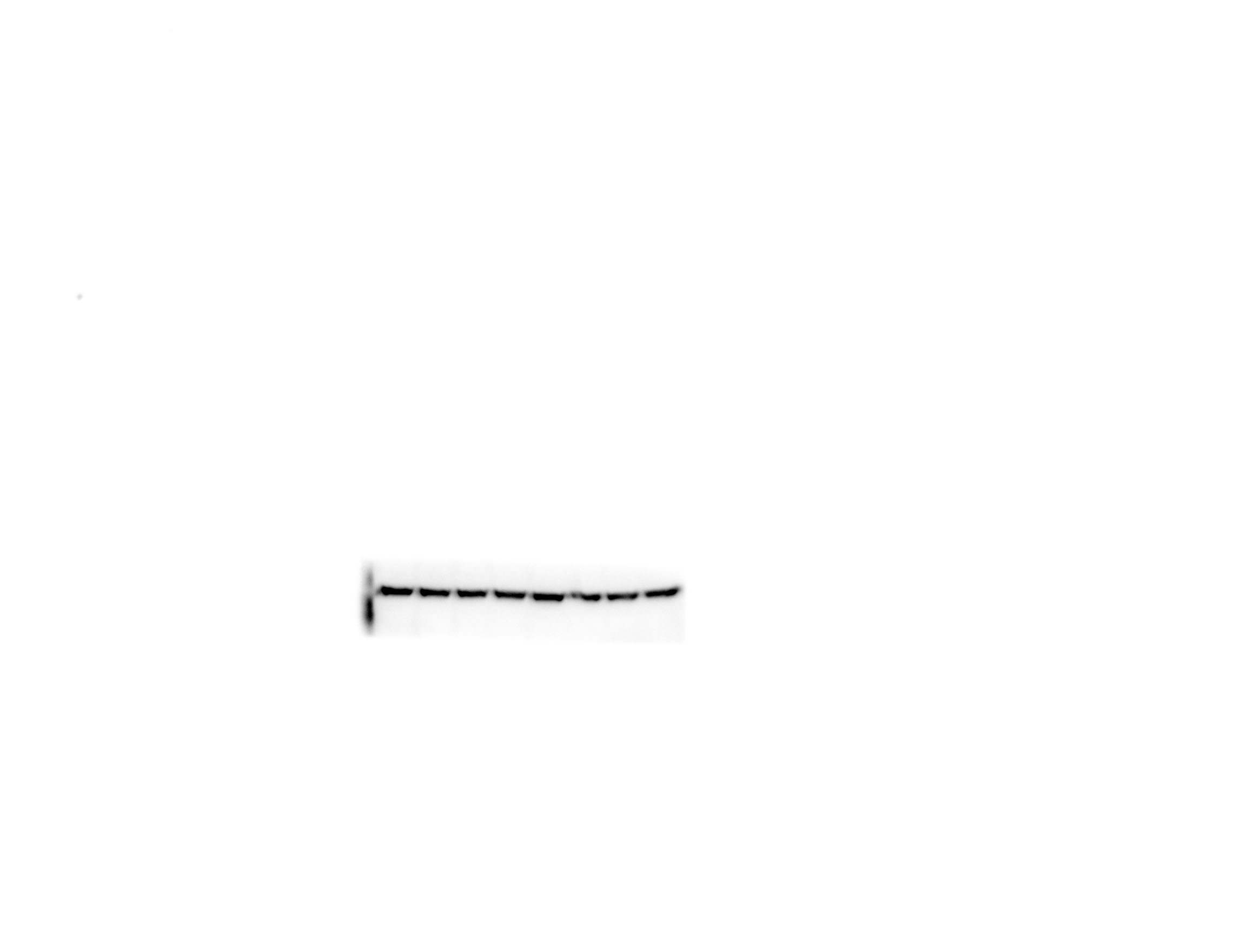

Supplement: Supplementary file 1 [file DataSheet1.ZIP › Original data/figure5-original data/DTT/Tubulin-DTT-1,2.jpg]

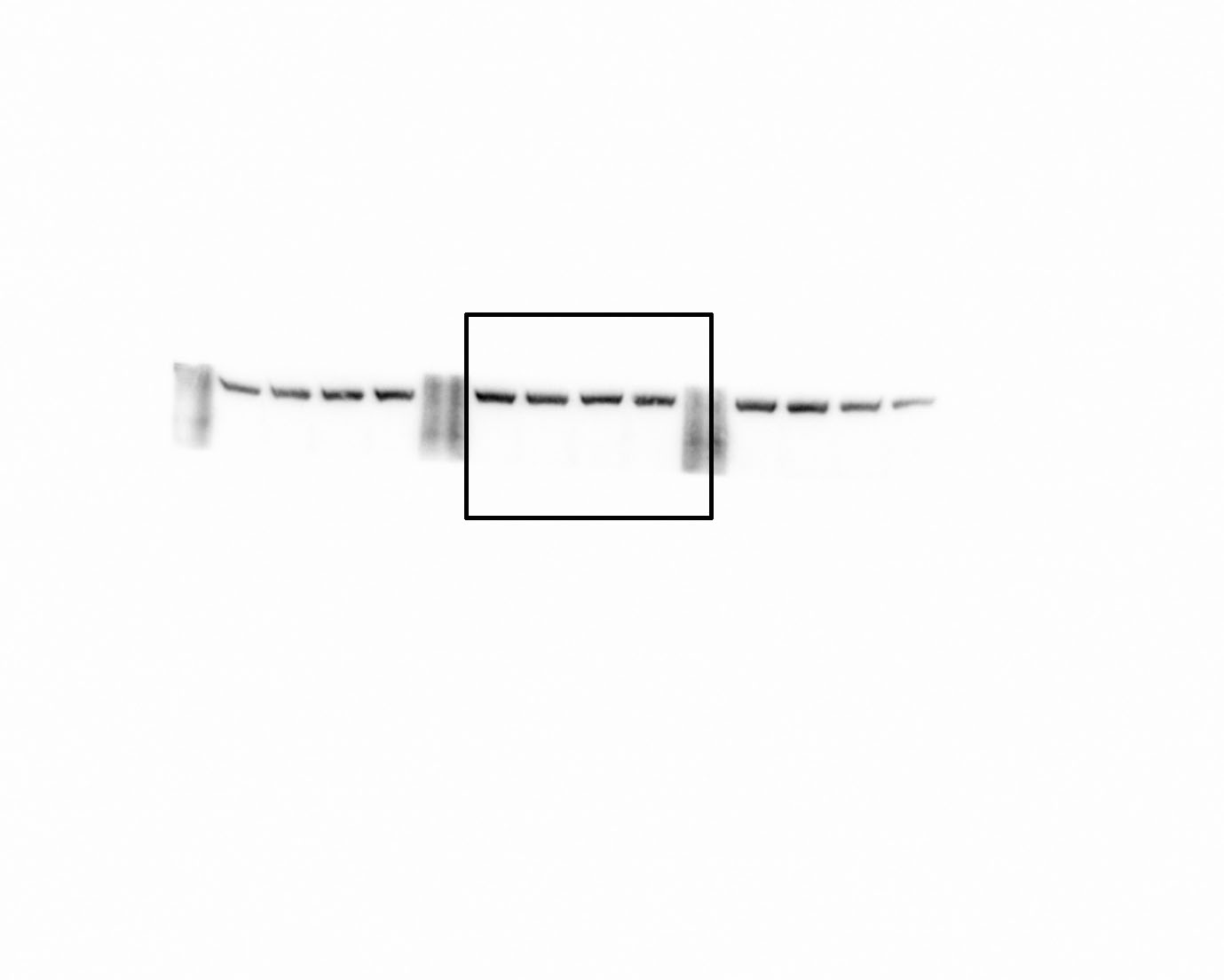

Supplement: Supplementary file 1 [file DataSheet1.ZIP › Original data/figure5-original data/DTT/Tubulin-DTT-3.jpg]

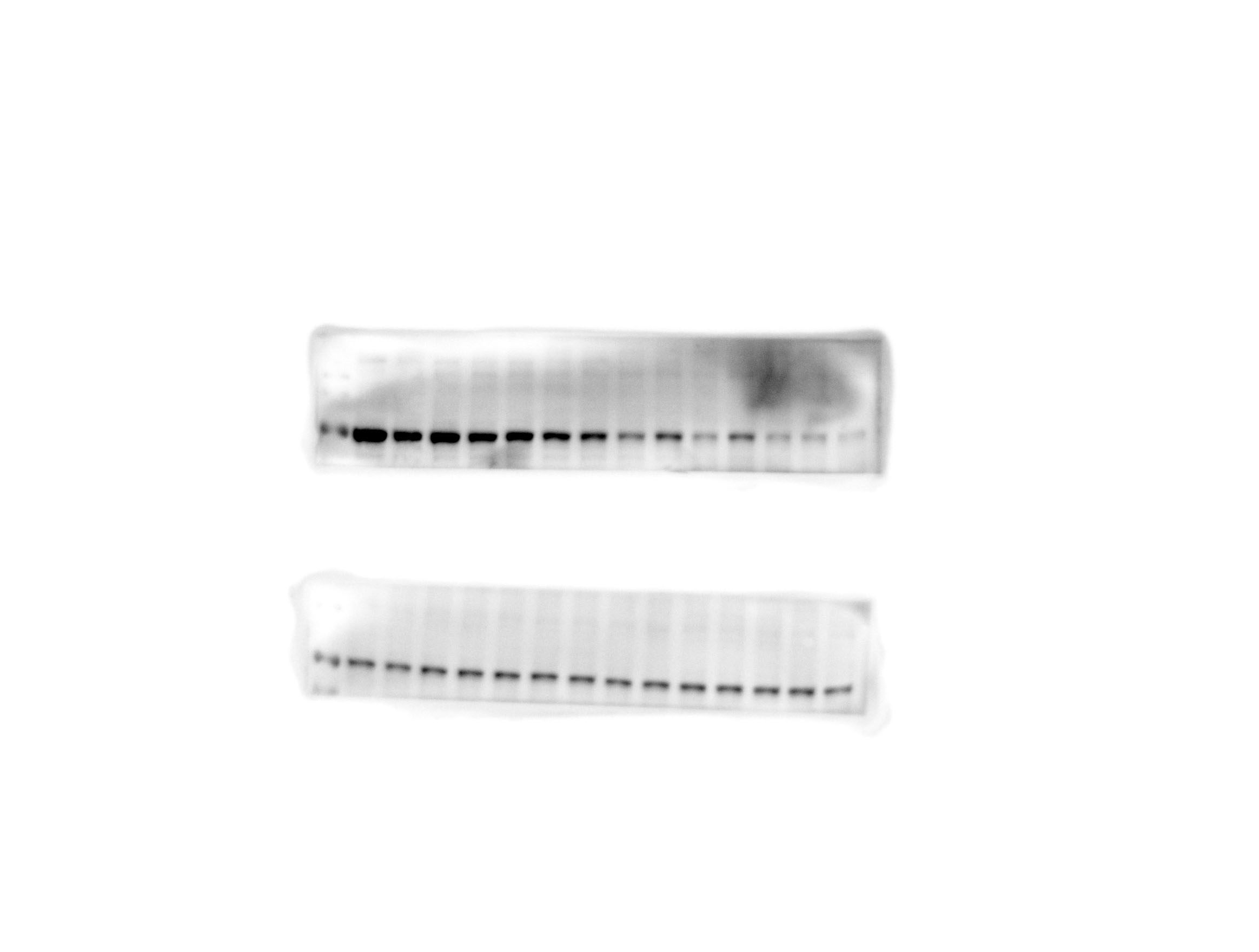

Supplement: Supplementary file 1 [file DataSheet1.ZIP › Original data/figure6-original data/CETSA/CETSA-BVA-IKK(上)+Tubulin(下)-1.jpg]

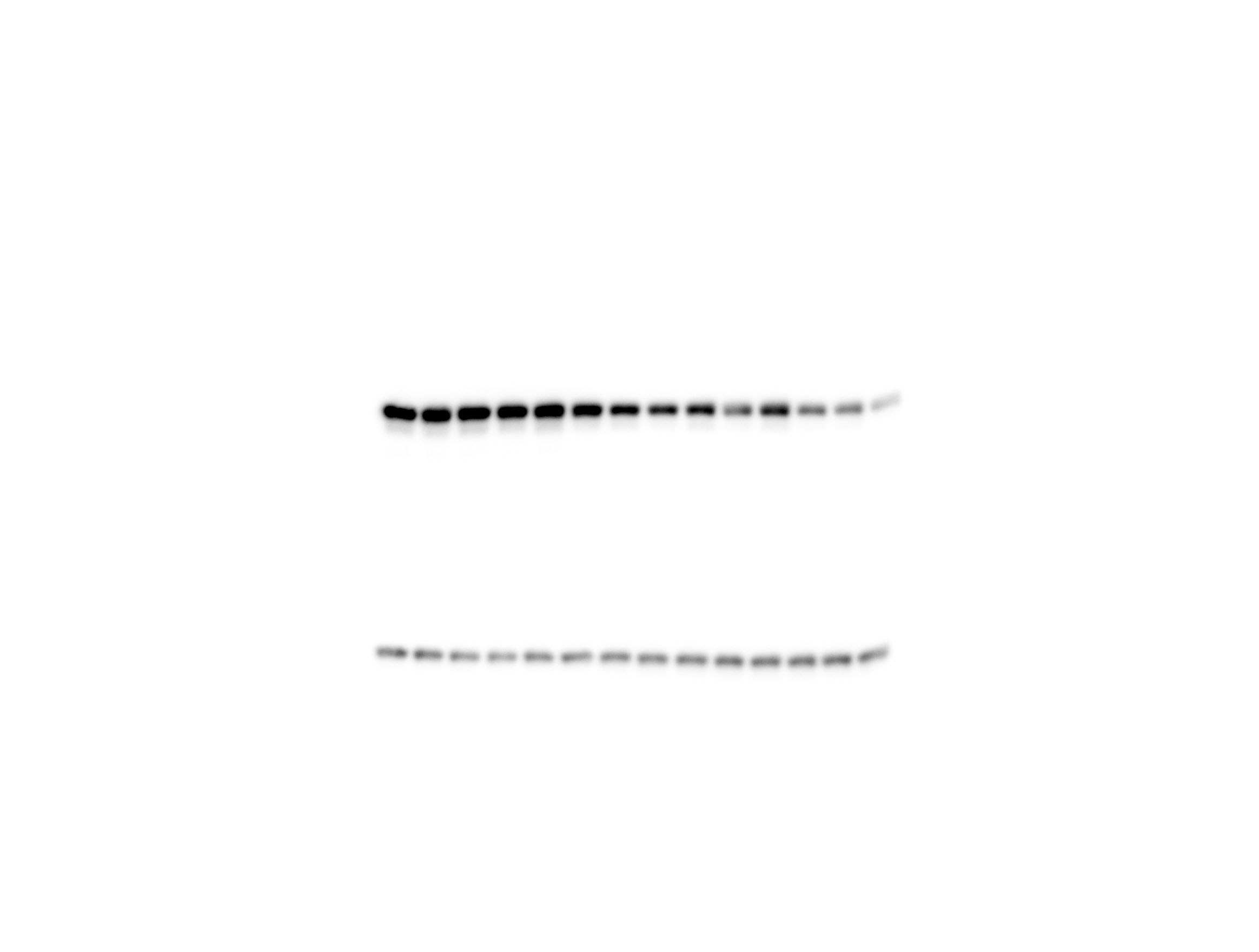

Supplement: Supplementary file 1 [file DataSheet1.ZIP › Original data/figure6-original data/CETSA/CETSA-BVA-IKK(上)+Tubulin(下)-3.jpg]

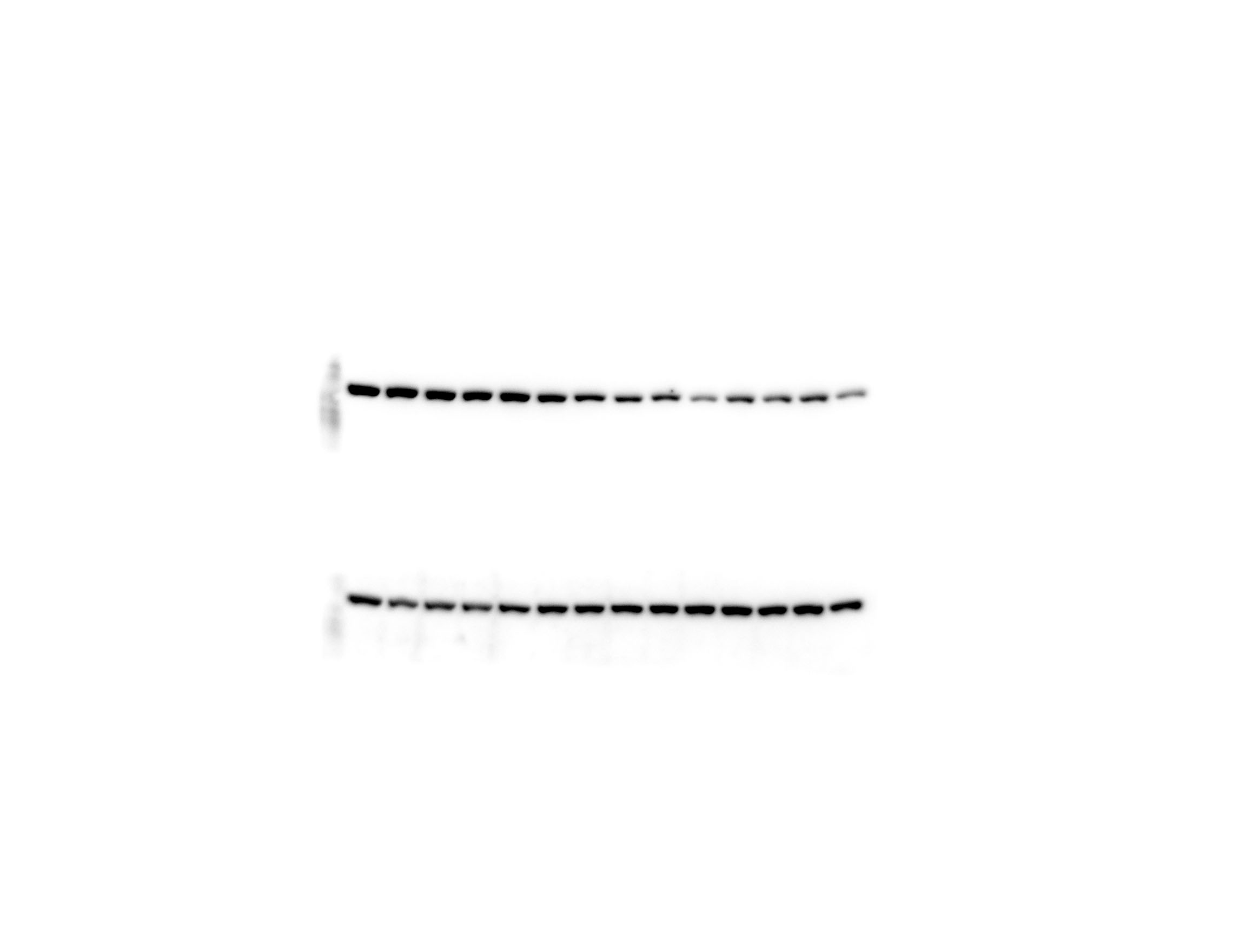

Supplement: Supplementary file 1 [file DataSheet1.ZIP › Original data/figure6-original data/CETSA/CETSA-BVA-IKK(上)-Tubulin(下)-BVA-2.jpg]

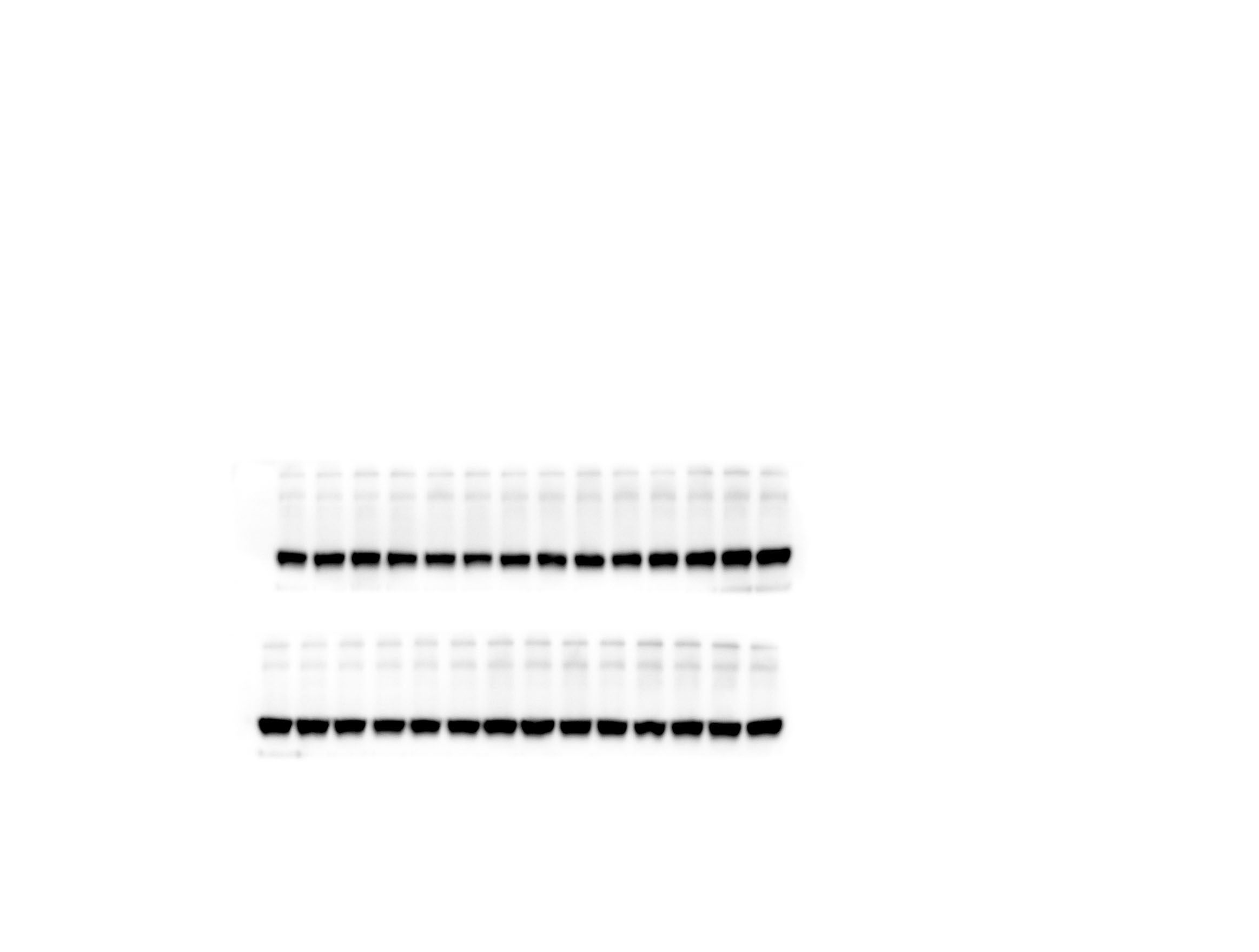

Supplement: Supplementary file 1 [file DataSheet1.ZIP › Original data/figure6-original data/CETSA/IKK-BVA-CETSA-Unheated-1,2.jpg]

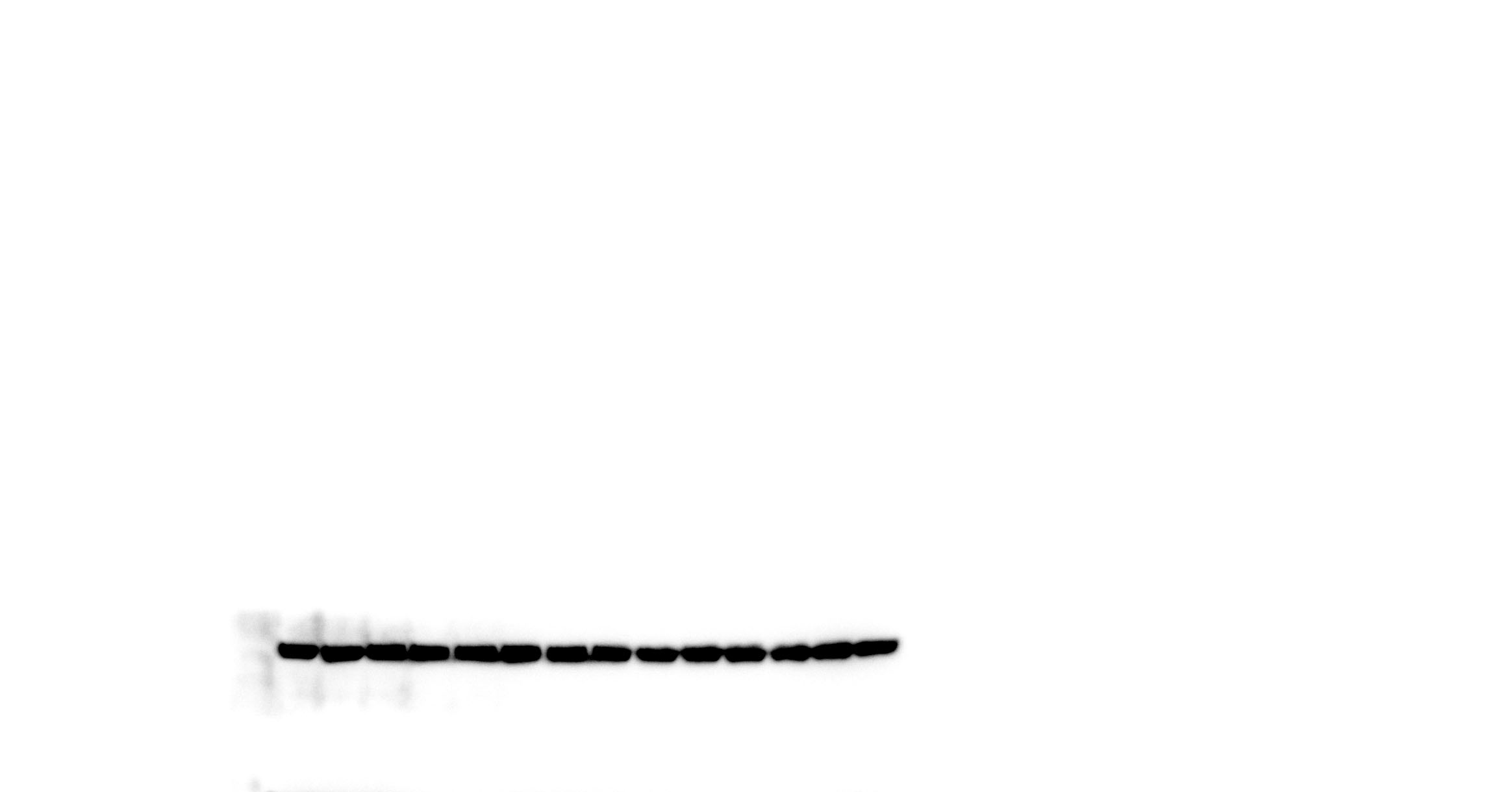

Supplement: Supplementary file 1 [file DataSheet1.ZIP › Original data/figure6-original data/CETSA/Tubulin-BVA-CETSA-Unheated-3.jpg]

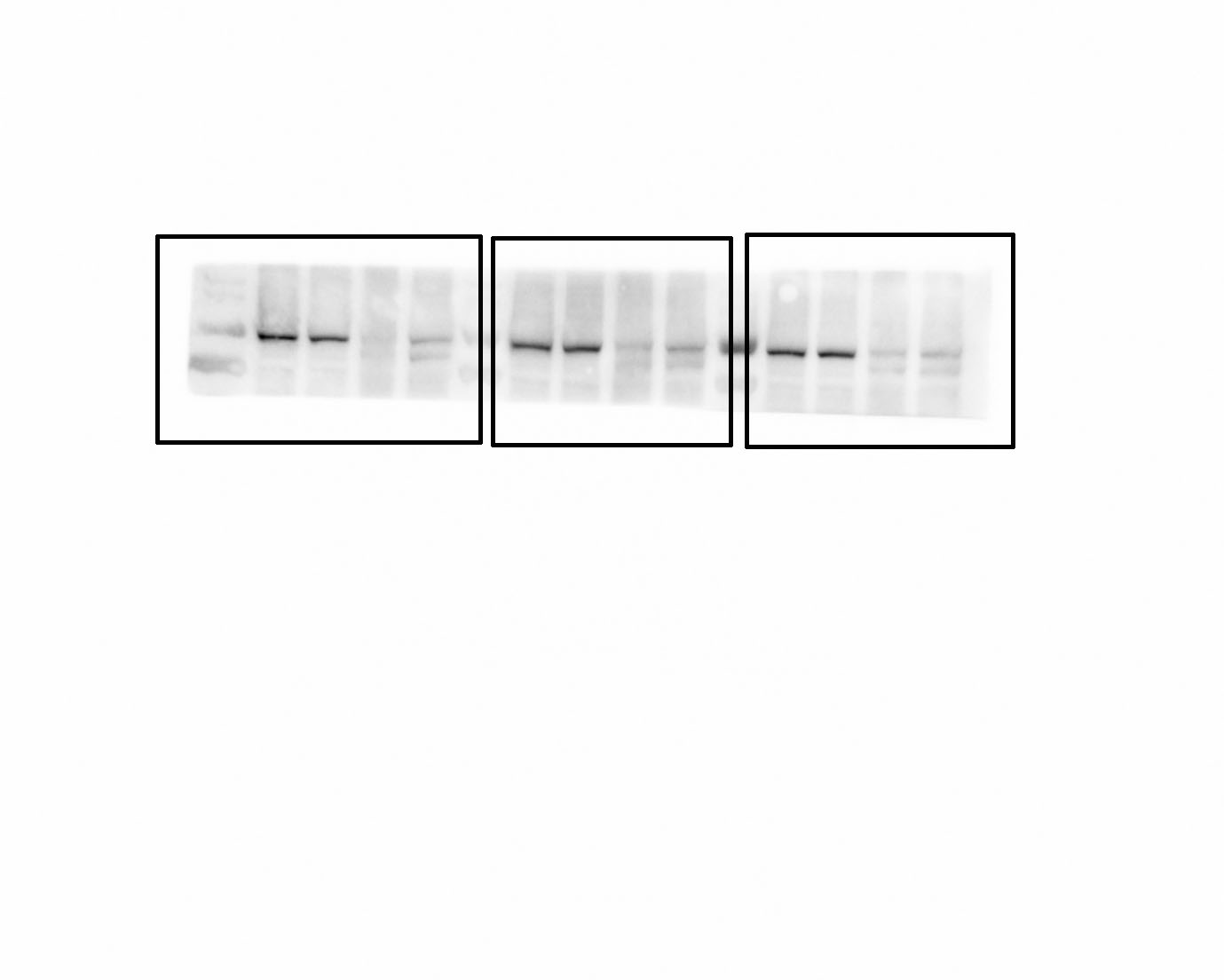

Supplement: Supplementary file 1 [file DataSheet1.ZIP › Original data/figure6-original data/DARTS-IKK/IKK-DARTS-1,2,3.jpg]

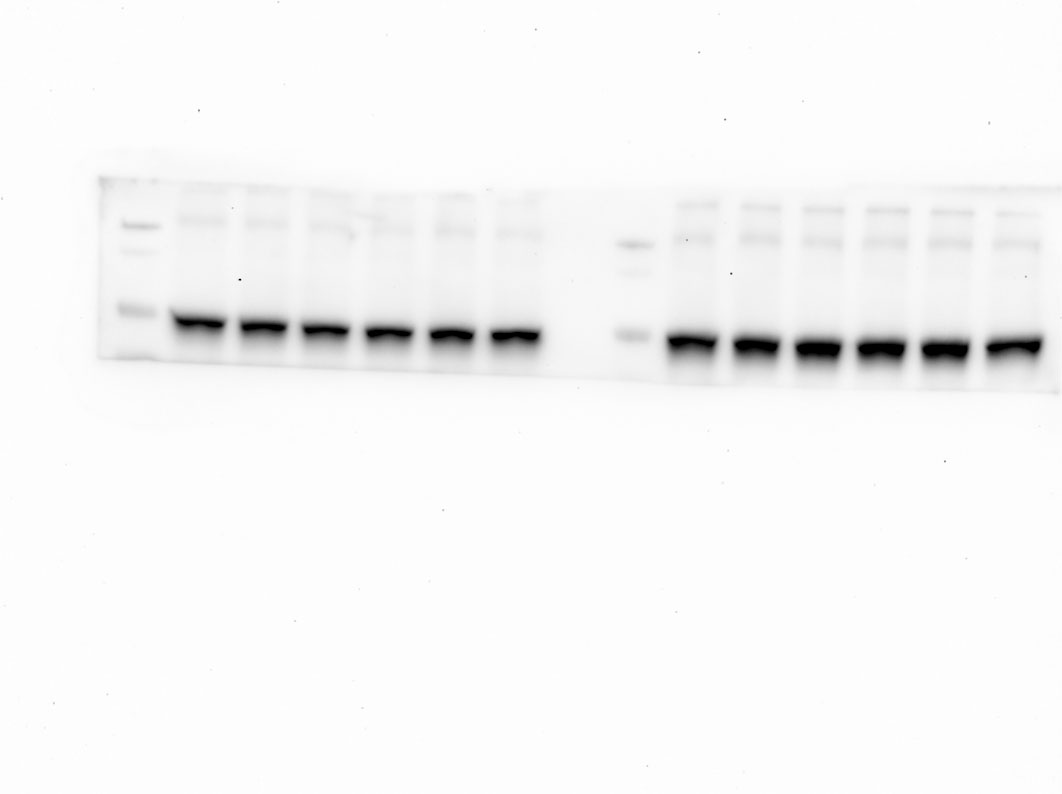

Supplement: Supplementary file 1 [file DataSheet1.ZIP › Original data/figure6-original data/IKK-LPSIFNγ/IKK-LPS+IFNγ-1,2.jpg]

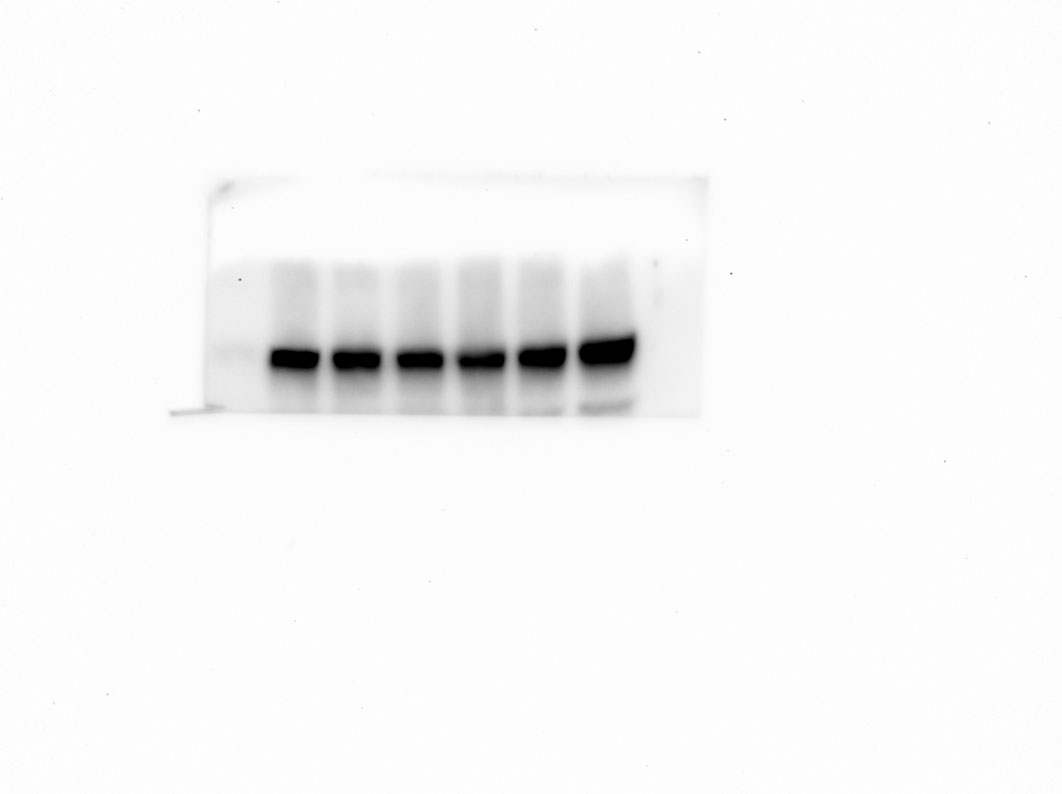

Supplement: Supplementary file 1 [file DataSheet1.ZIP › Original data/figure6-original data/IKK-LPSIFNγ/IKK-LPS+IFNγ-3.jpg]

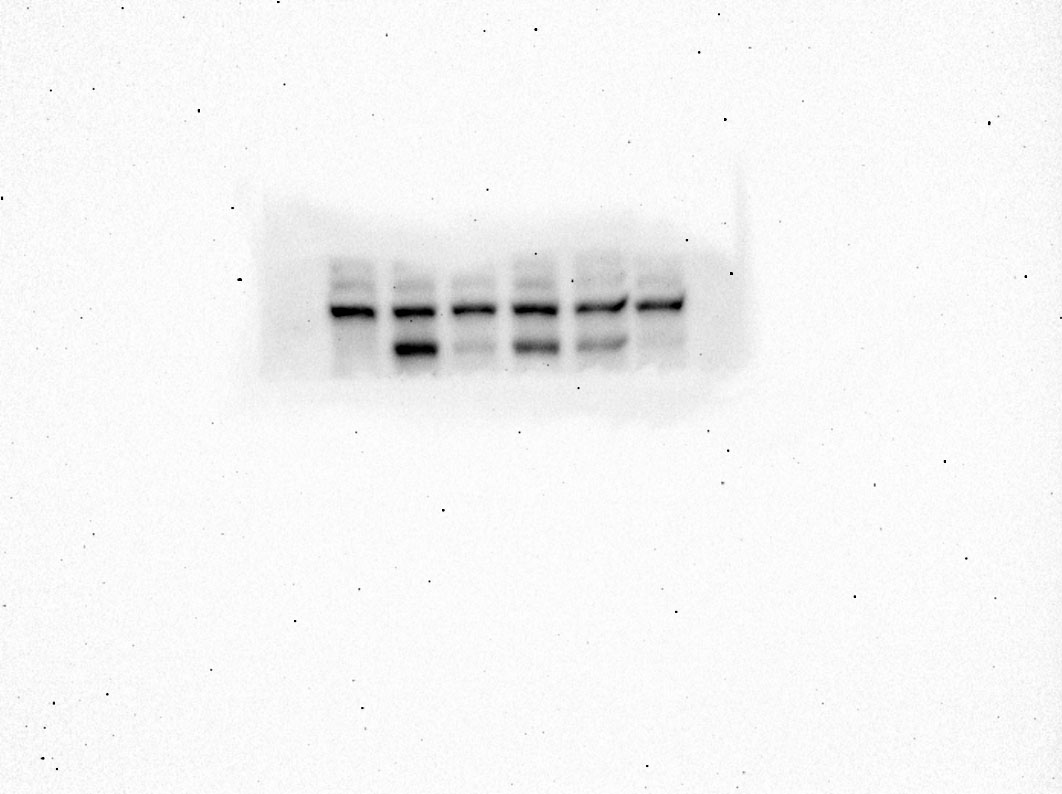

Supplement: Supplementary file 1 [file DataSheet1.ZIP › Original data/figure6-original data/IKK-LPSIFNγ/P-IKK-LPS+IFNγ-1.jpg]

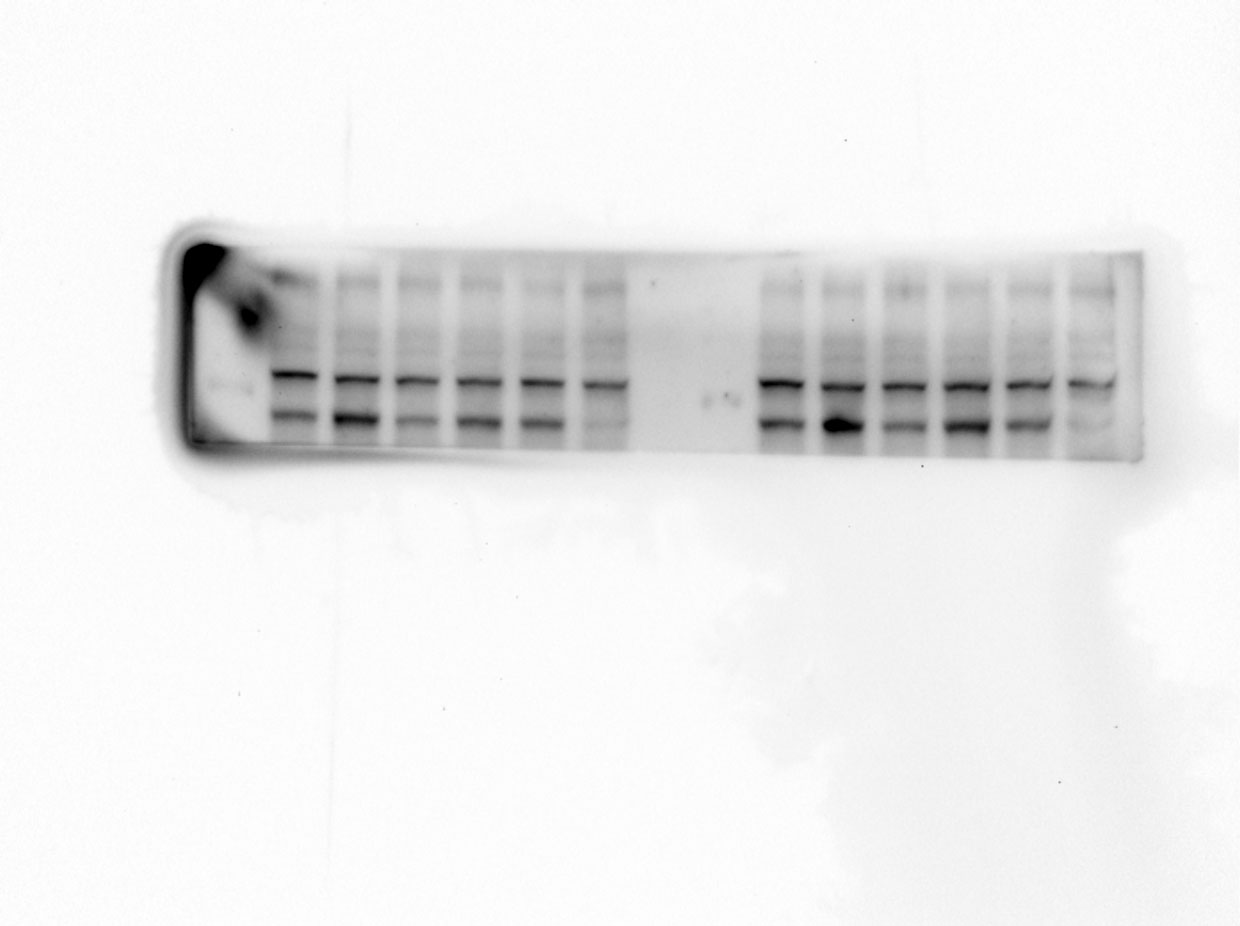

Supplement: Supplementary file 1 [file DataSheet1.ZIP › Original data/figure6-original data/IKK-LPSIFNγ/P-IKK-LPS+IFNγ-2,3.jpg]

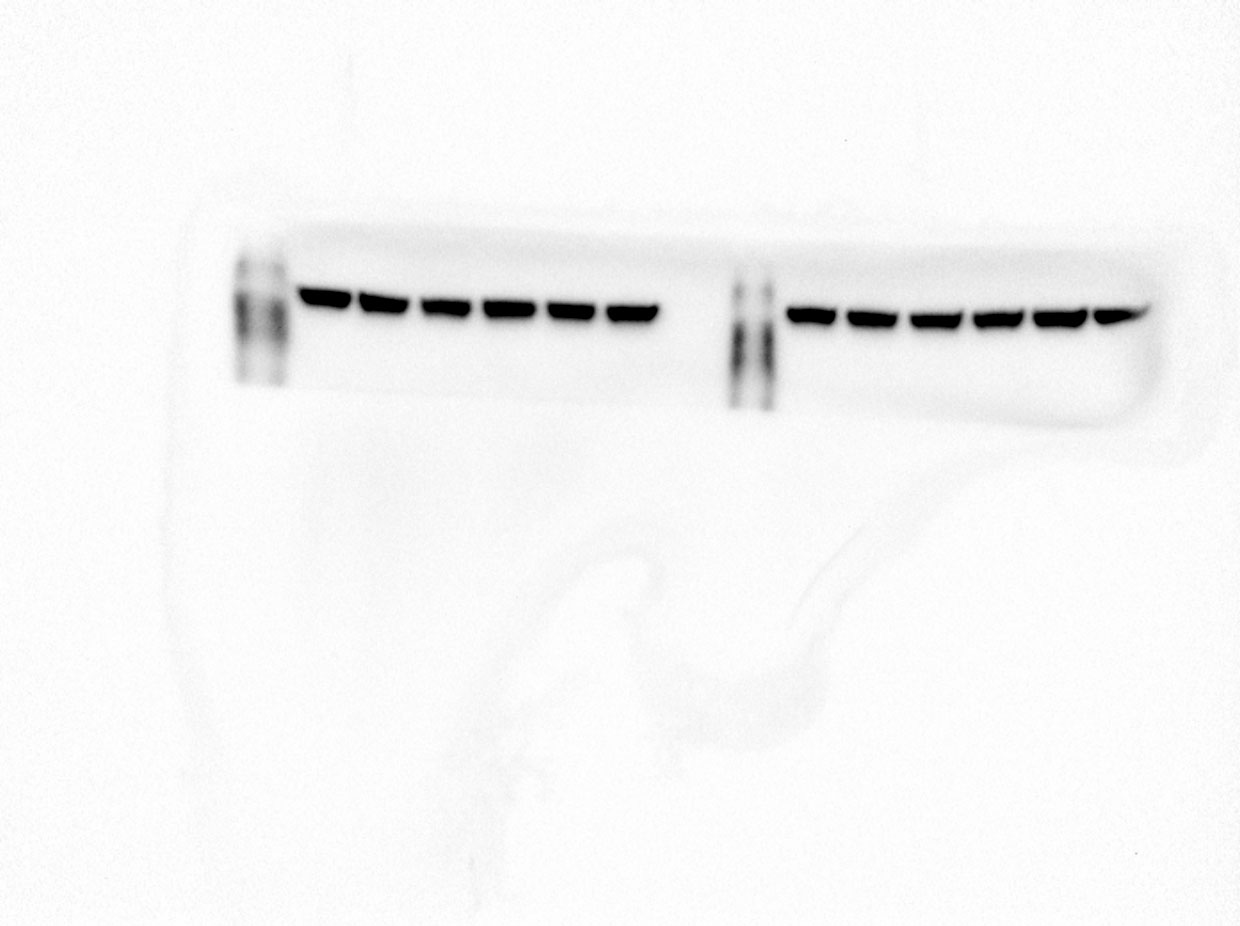

Supplement: Supplementary file 1 [file DataSheet1.ZIP › Original data/figure6-original data/IKK-LPSIFNγ/Tubulin-IKK+P-IKK+LPS+IFNγ-1,2.jpg]

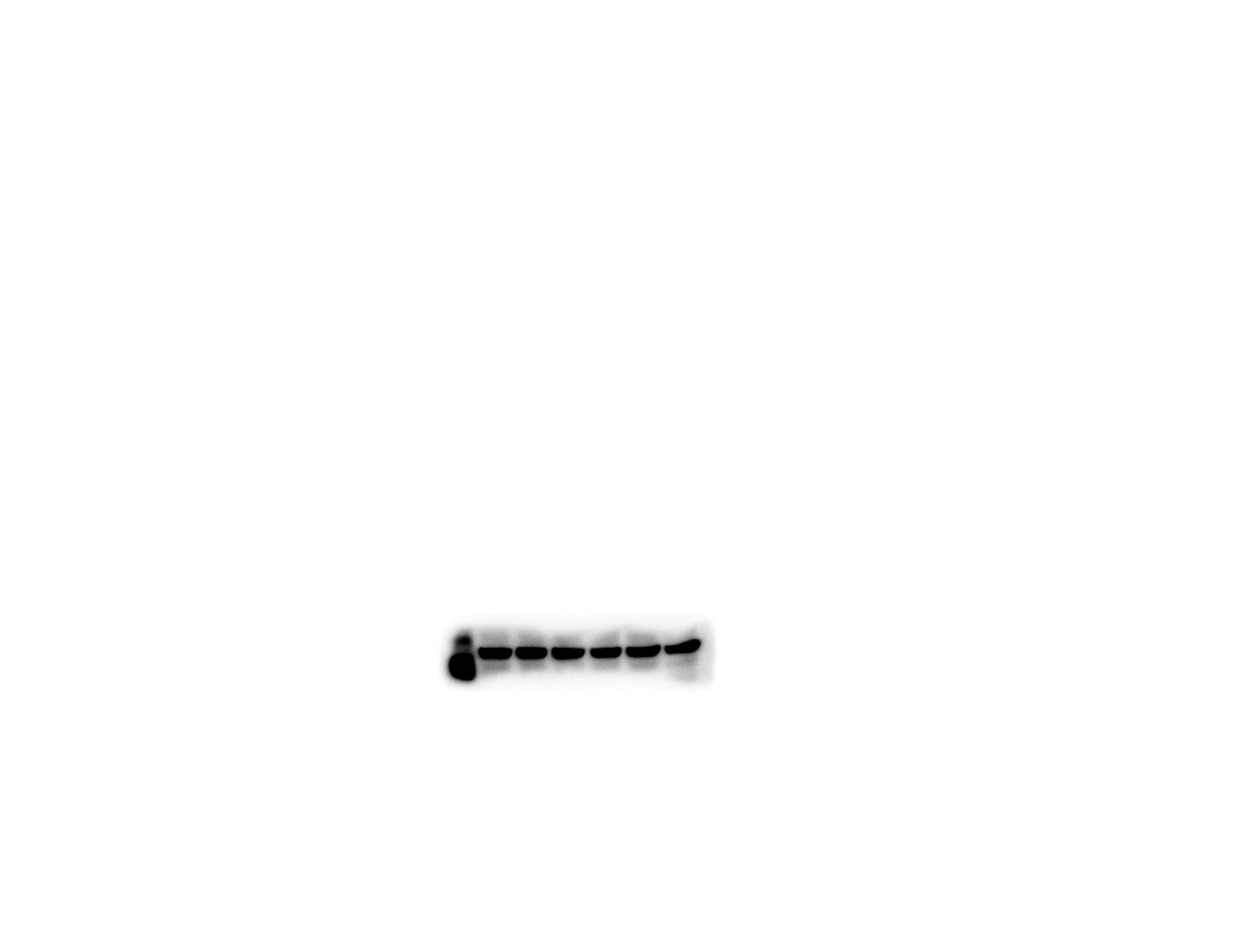

Supplement: Supplementary file 1 [file DataSheet1.ZIP › Original data/figure6-original data/IKK-LPSIFNγ/Tubulin-IKK+P-IKK+LPS+IFNγ-3.jpg]

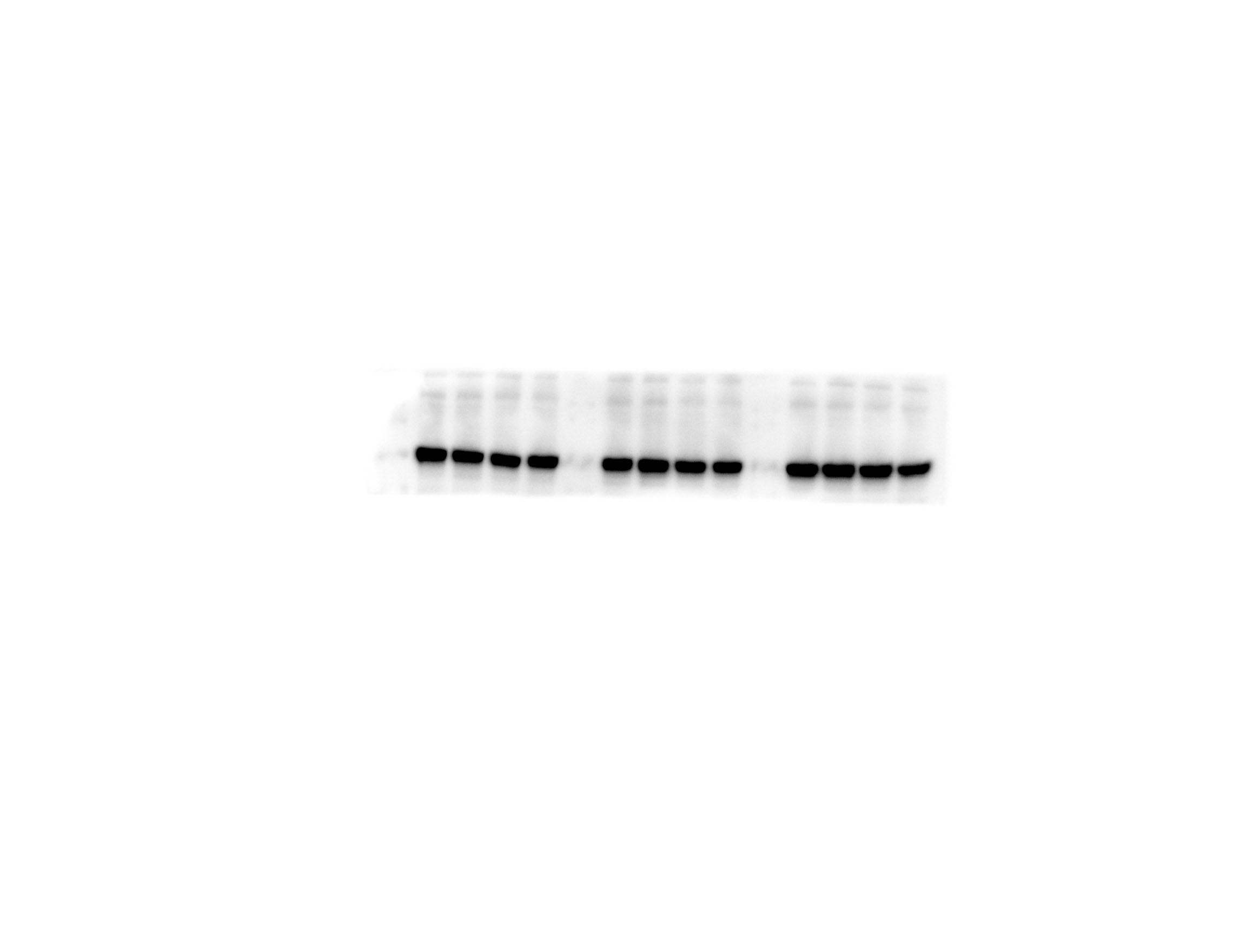

Supplement: Supplementary file 1 [file DataSheet1.ZIP › Original data/figure6-original data/IKK-TNF+IFNγ/IKK-TNFα+IFNγ-1,2,3.jpg]

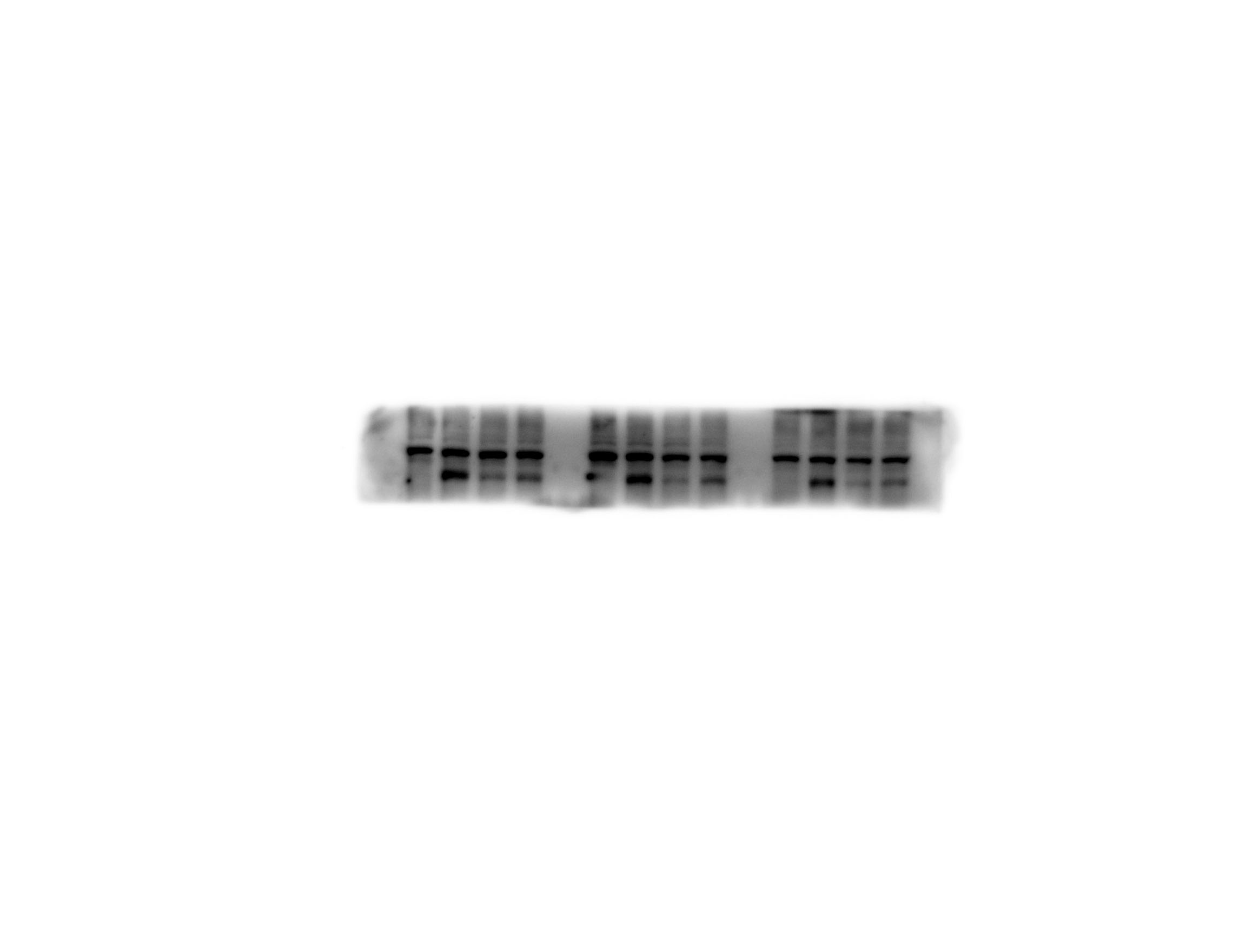

Supplement: Supplementary file 1 [file DataSheet1.ZIP › Original data/figure6-original data/IKK-TNF+IFNγ/P-IKK-TNFα+IFNγ-1,2,3.jpg]

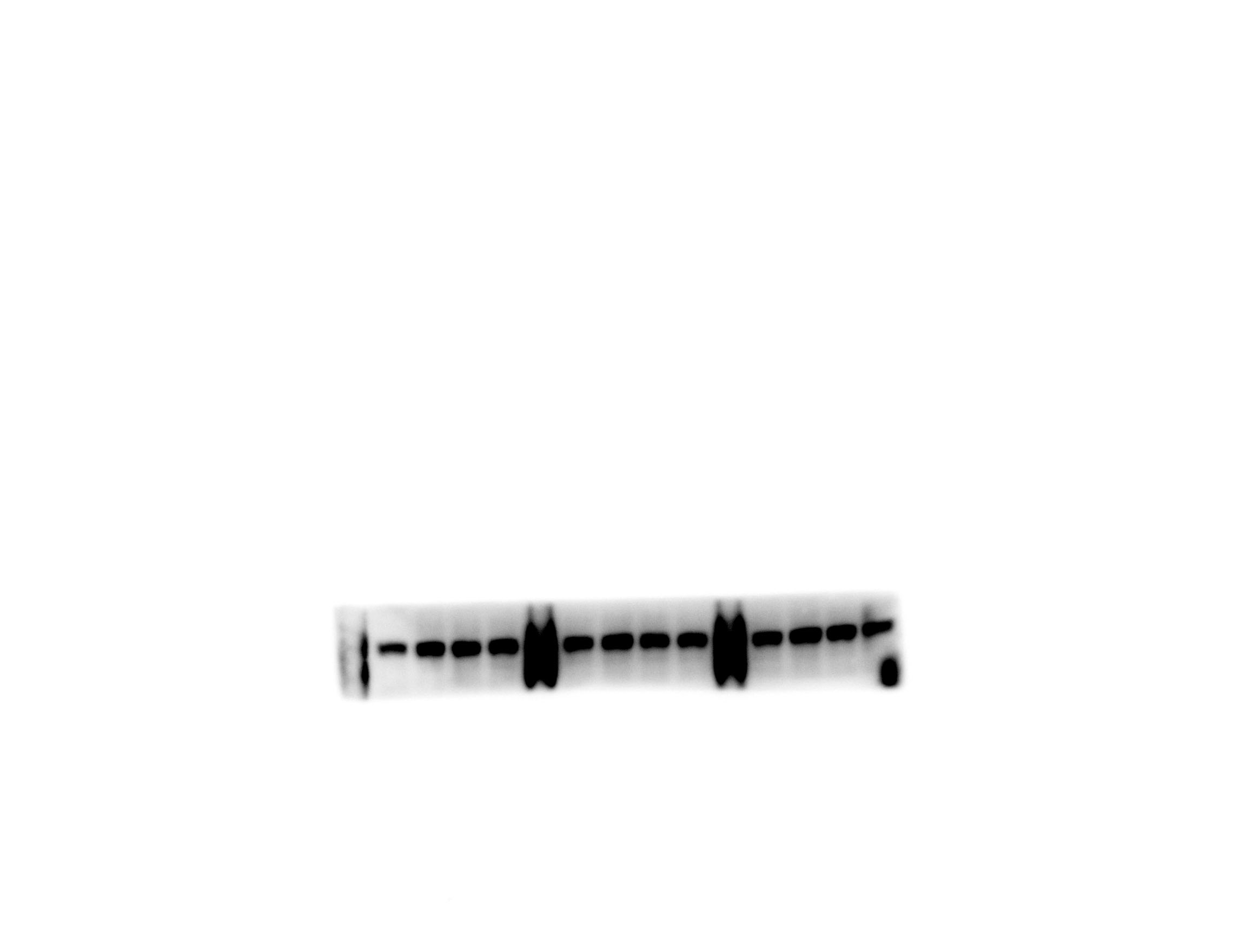

Supplement: Supplementary file 1 [file DataSheet1.ZIP › Original data/figure6-original data/IKK-TNF+IFNγ/Tubulin-1,2,3.jpg]

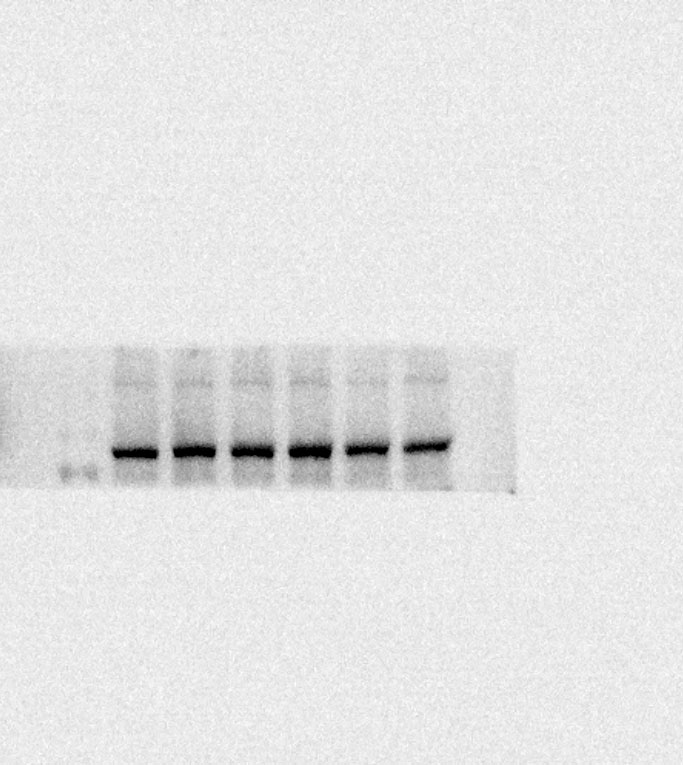

Supplement: Supplementary file 1 [file DataSheet1.ZIP › Original data/figure6-original data/Kinase assay-IKK/Flag-IKK-1.jpg]

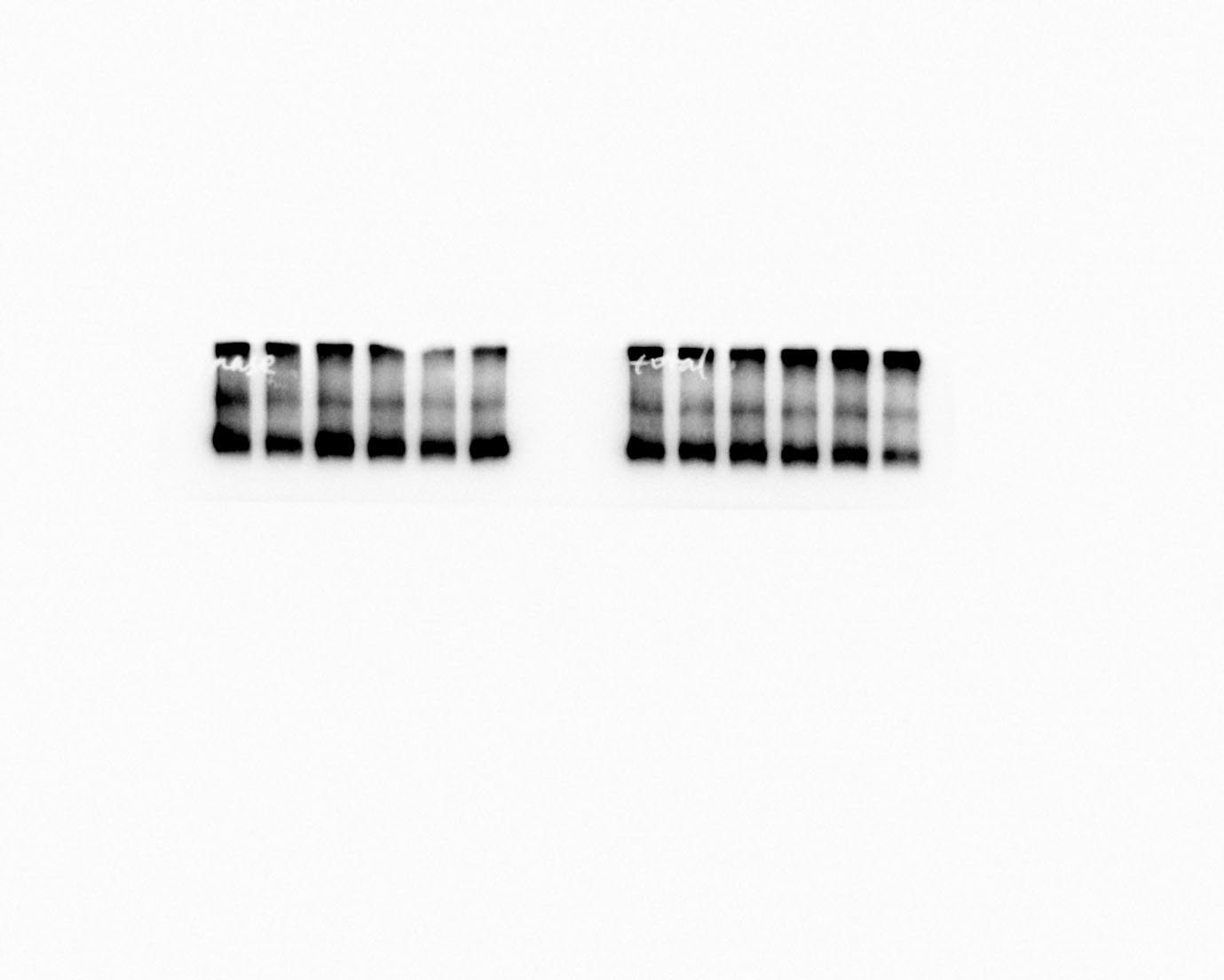

Supplement: Supplementary file 1 [file DataSheet1.ZIP › Original data/figure6-original data/Kinase assay-IKK/Flag-IKK-2,3.jpg]

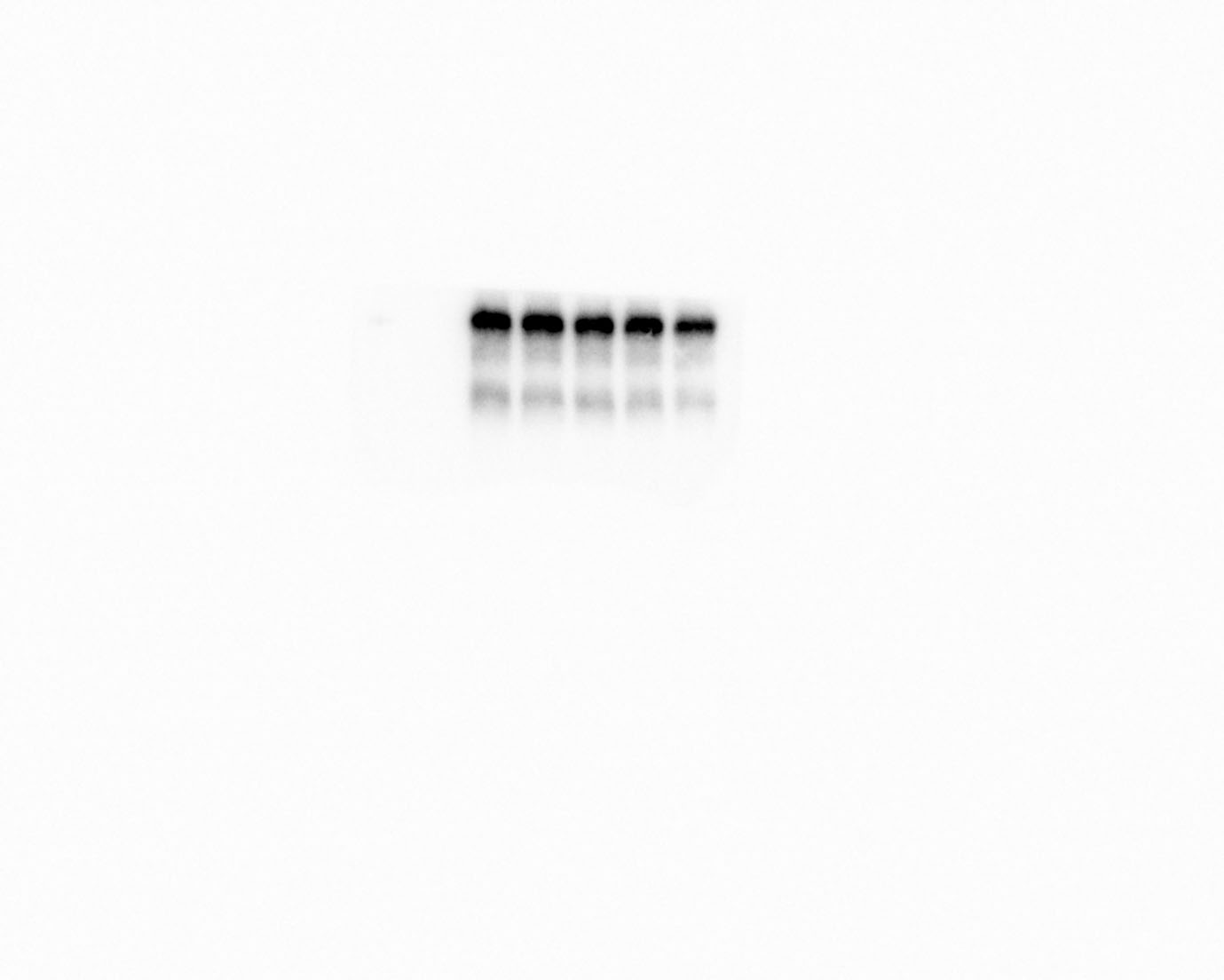

Supplement: Supplementary file 1 [file DataSheet1.ZIP › Original data/figure6-original data/Kinase assay-IKK/IκBα-1.jpg]

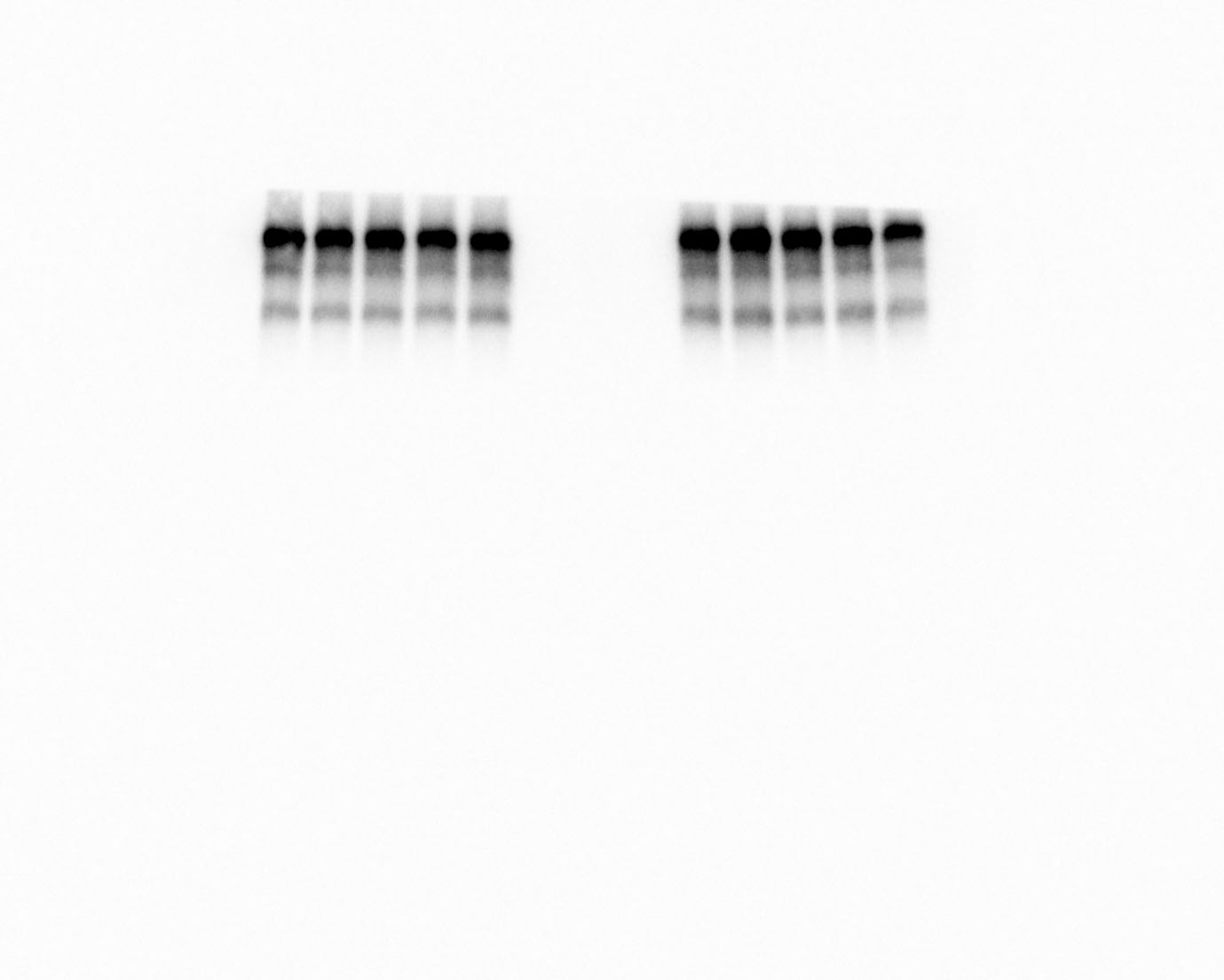

Supplement: Supplementary file 1 [file DataSheet1.ZIP › Original data/figure6-original data/Kinase assay-IKK/IκBα-2,3.jpg]

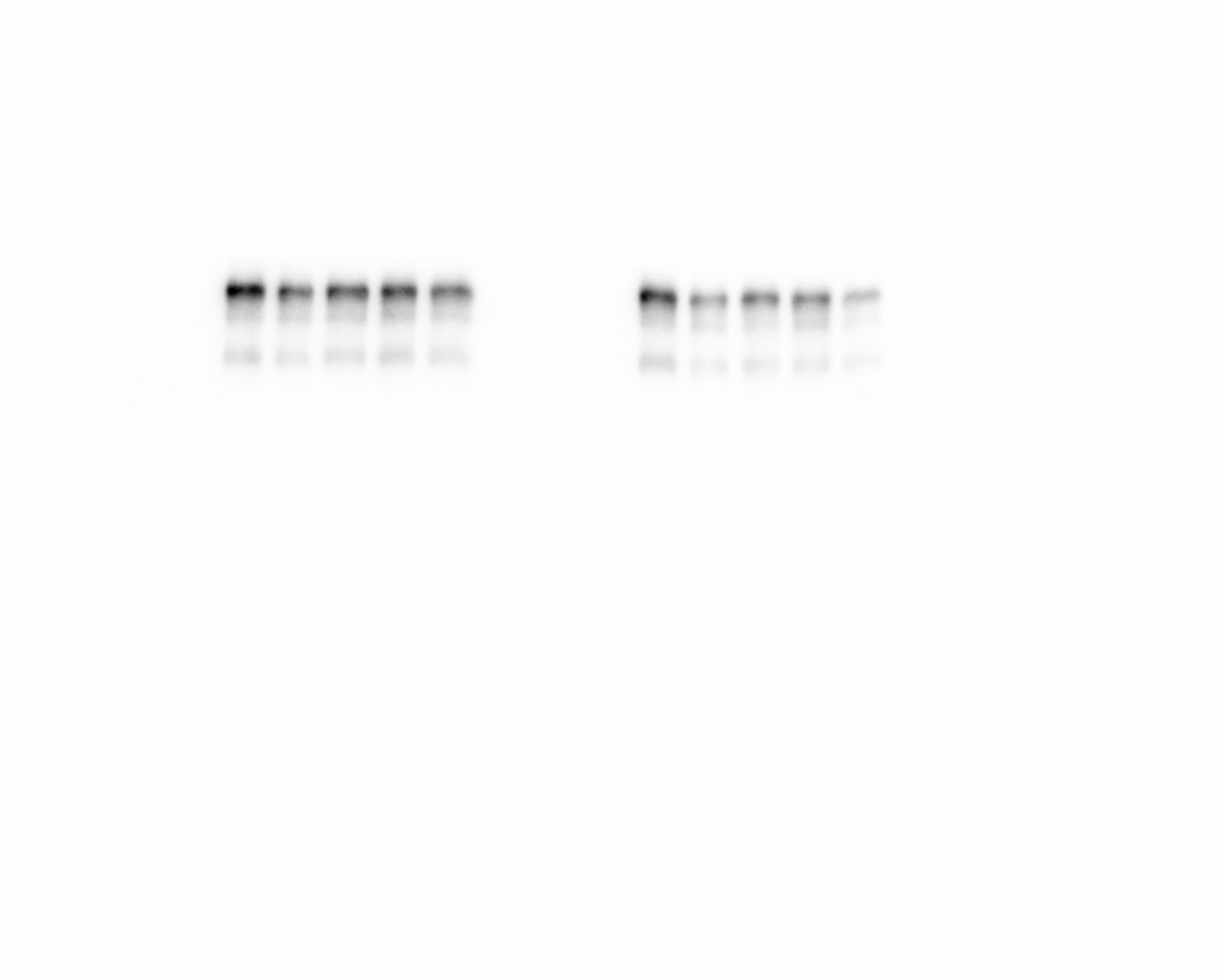

Supplement: Supplementary file 1 [file DataSheet1.ZIP › Original data/figure6-original data/Kinase assay-IKK/P-IκBα-1,2.jpg]

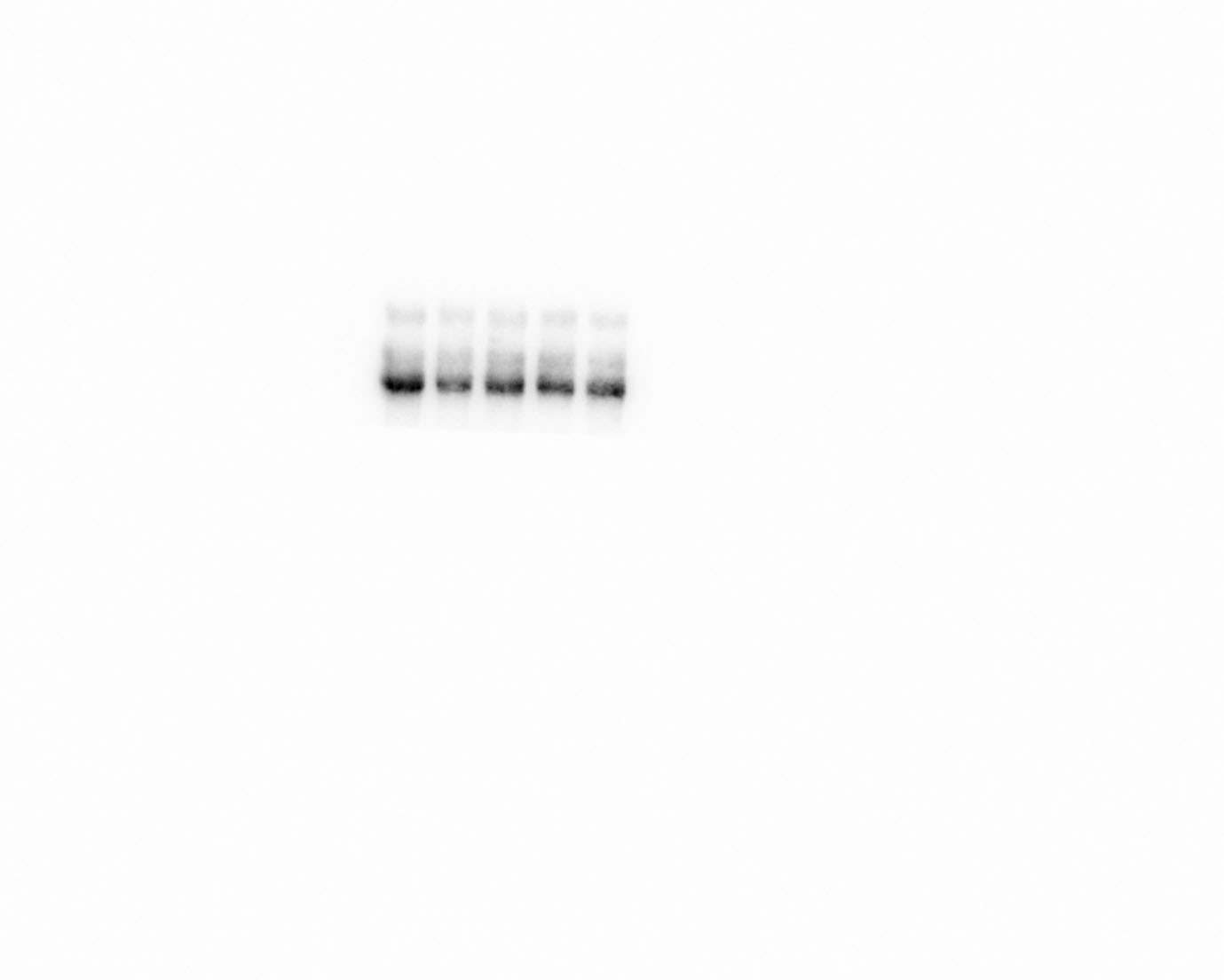

Supplement: Supplementary file 1 [file DataSheet1.ZIP › Original data/figure6-original data/Kinase assay-IKK/P-IκBα-3.jpg]

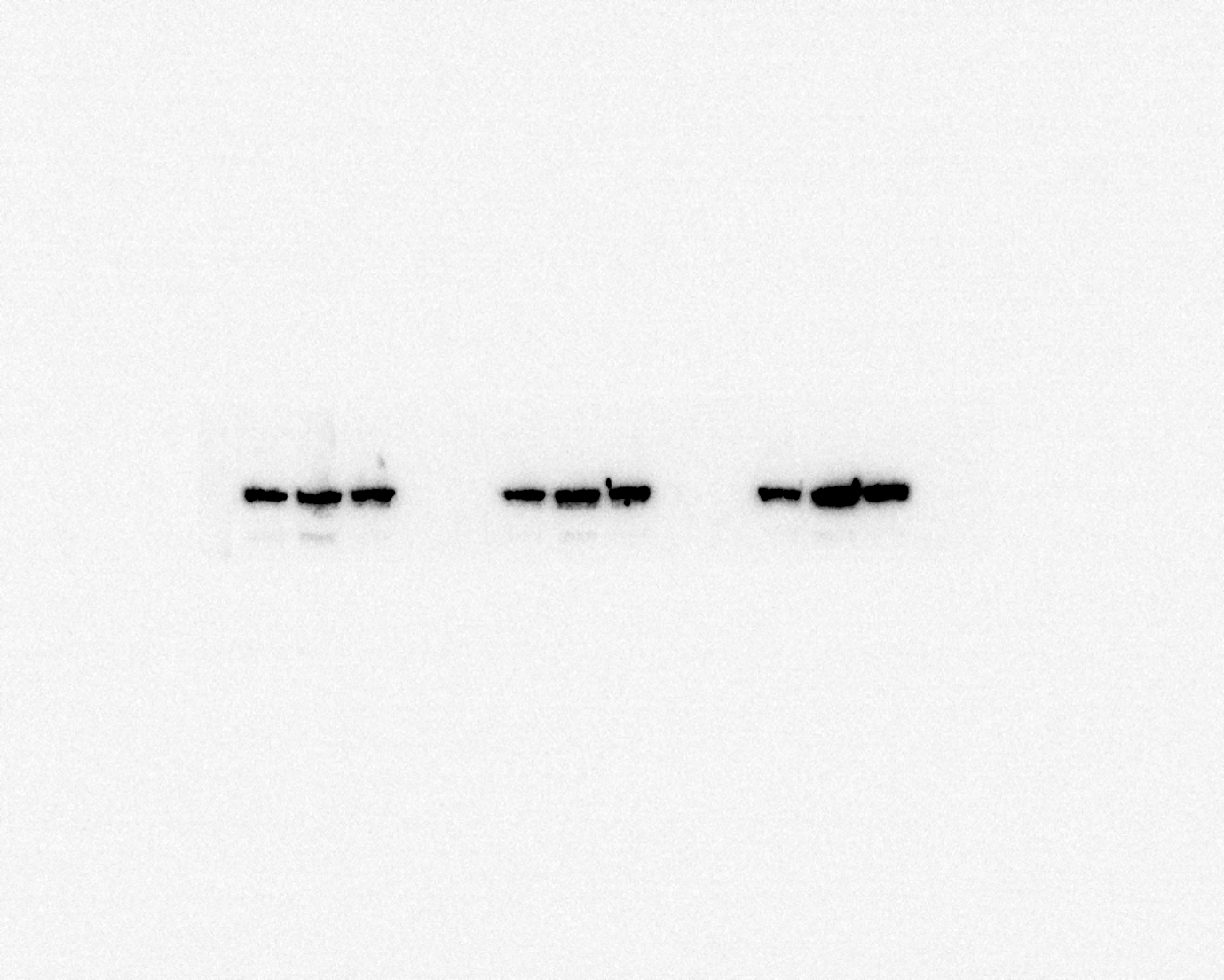

Supplement: Supplementary file 1 [file DataSheet1.ZIP › Original data/Supplementary figure 4-original data/IκBα-MG132-1,2,3.tif]

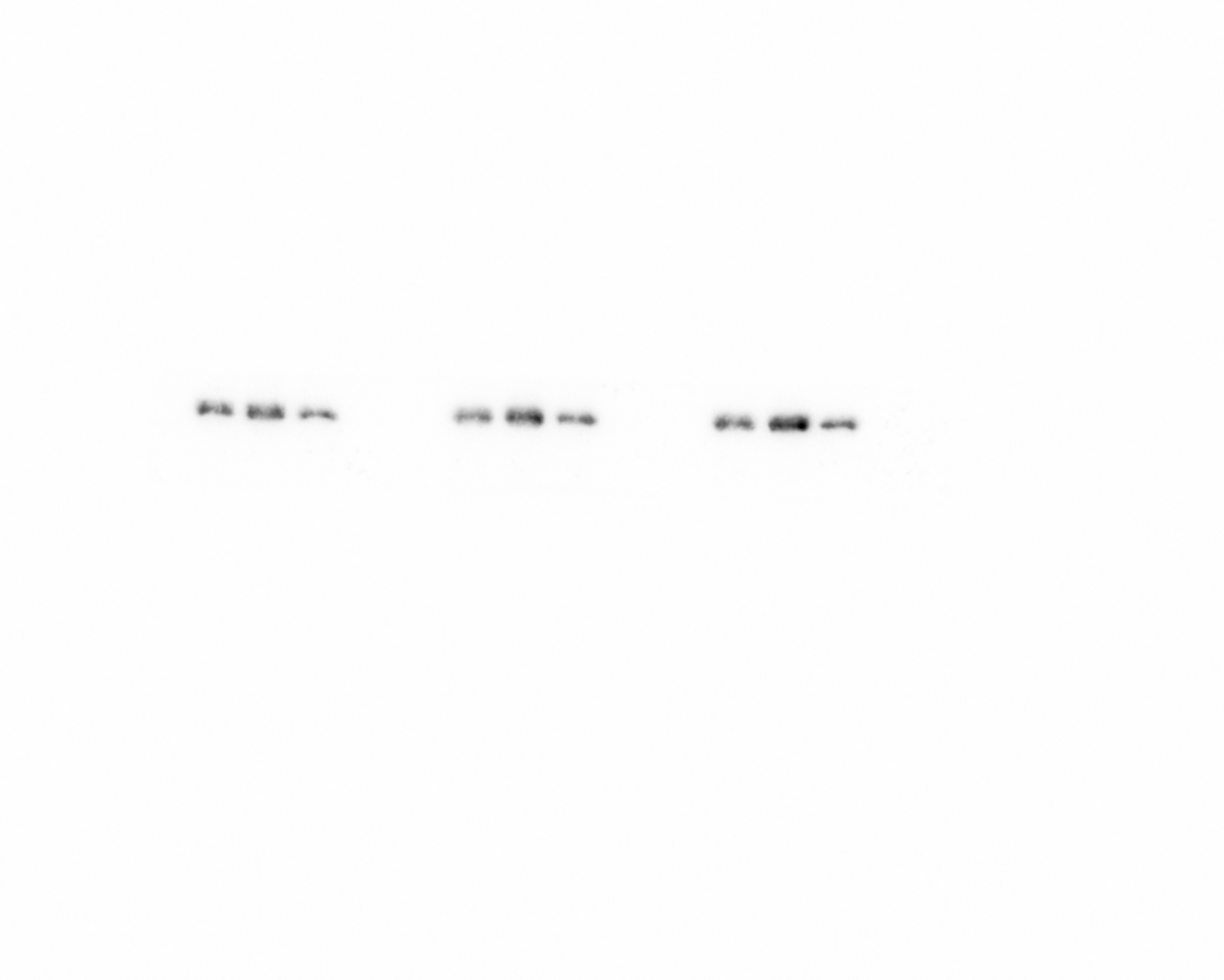

Supplement: Supplementary file 1 [file DataSheet1.ZIP › Original data/Supplementary figure 4-original data/P-IκBα-MG132-1,2,3.tif]

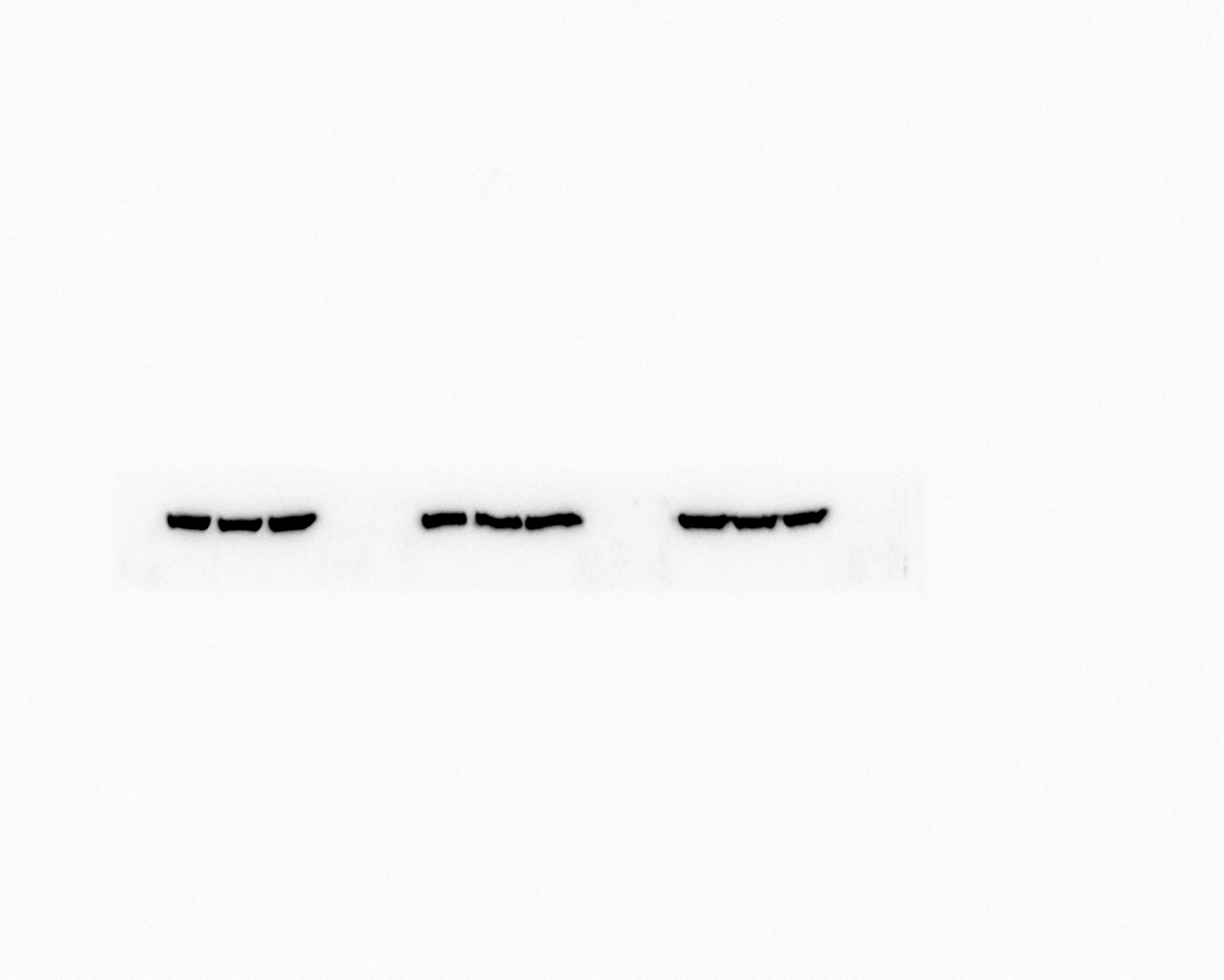

Supplement: Supplementary file 1 [file DataSheet1.ZIP › Original data/Supplementary figure 4-original data/Tubulin-MG132-1,2,3.tif]

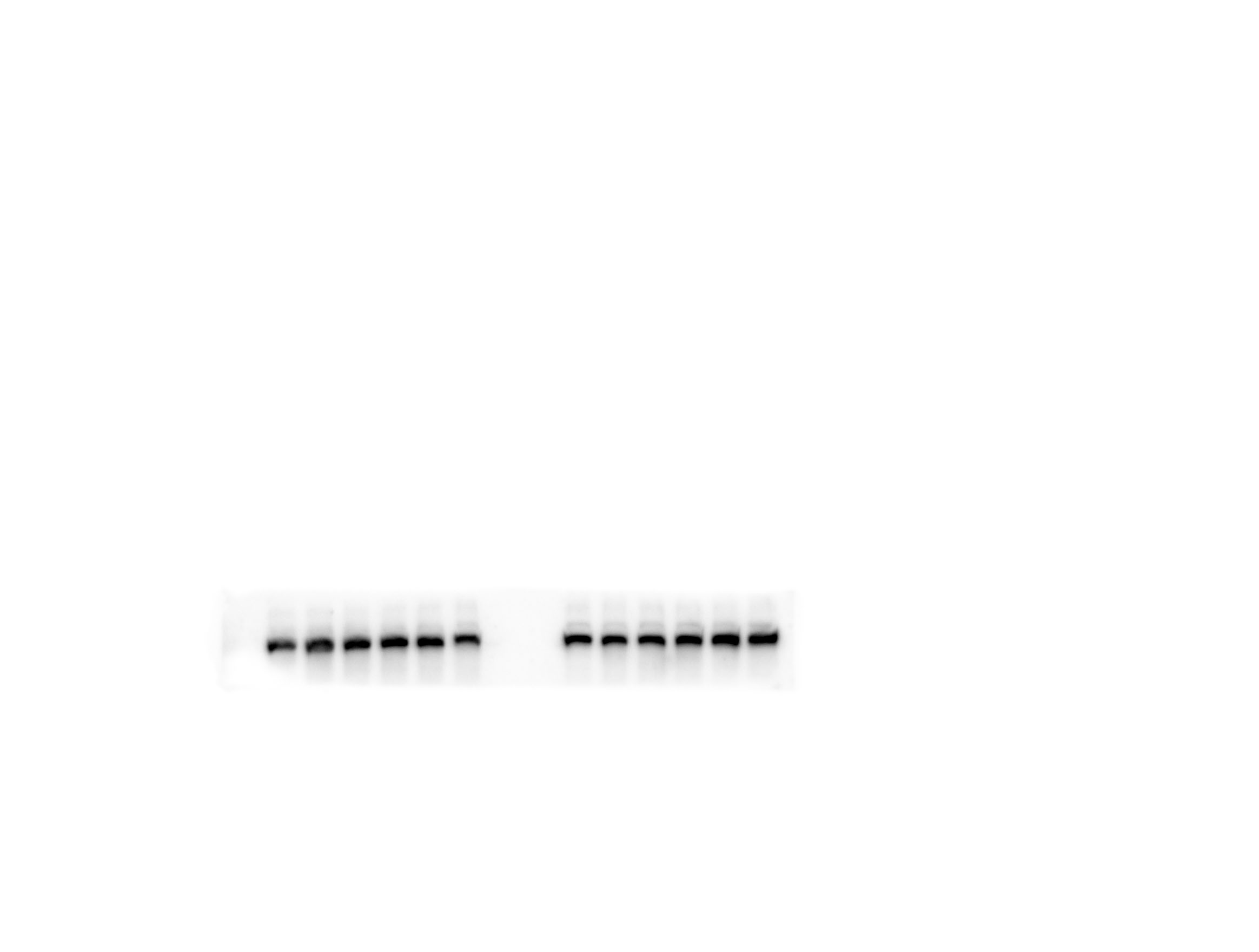

Supplement: Supplementary file 1 [file DataSheet1.ZIP › Original data/Supplementary figure 5-original data/AKT+P-AKT/AKT-1,2.jpg]

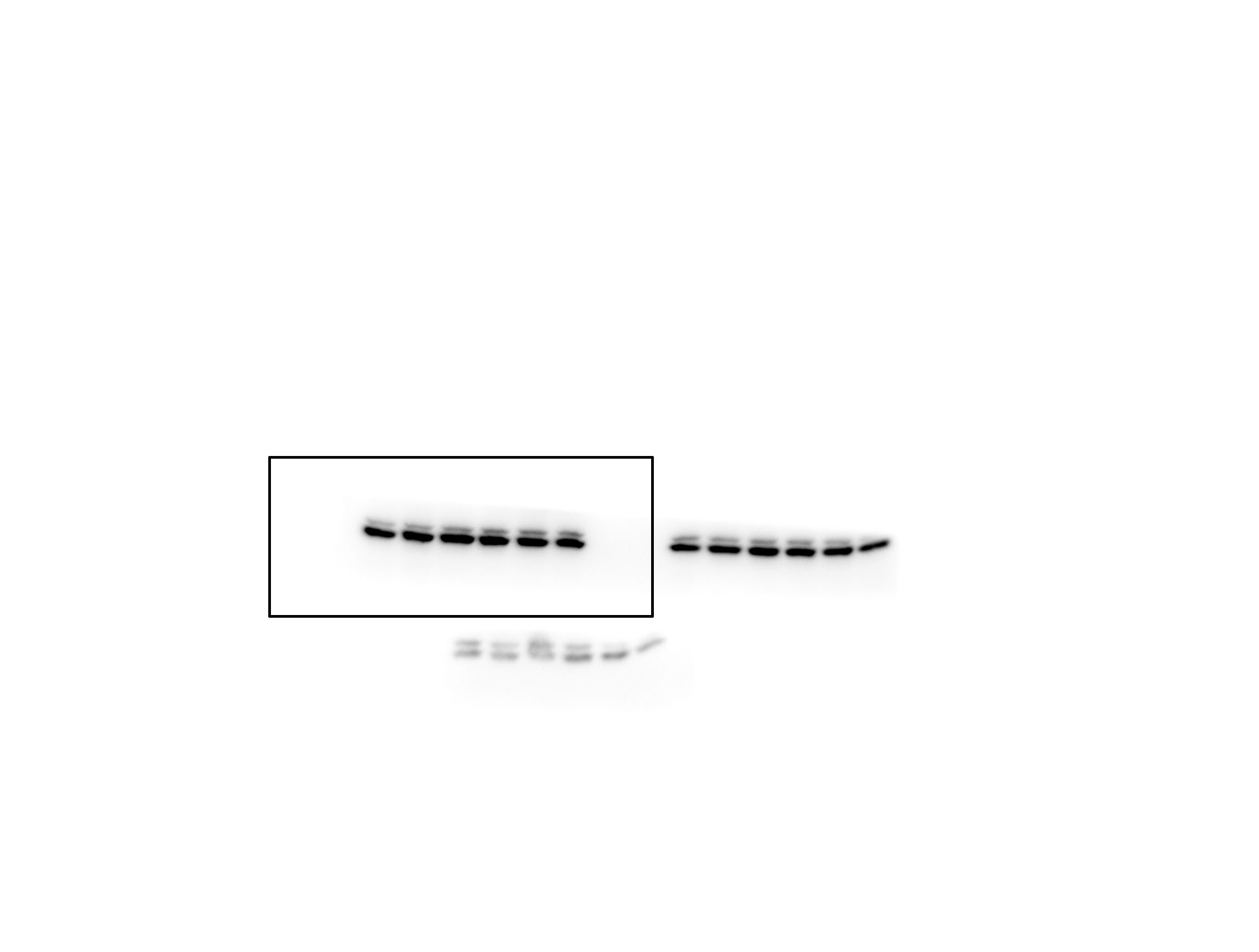

Supplement: Supplementary file 1 [file DataSheet1.ZIP › Original data/Supplementary figure 5-original data/AKT+P-AKT/AKT-3.jpg]

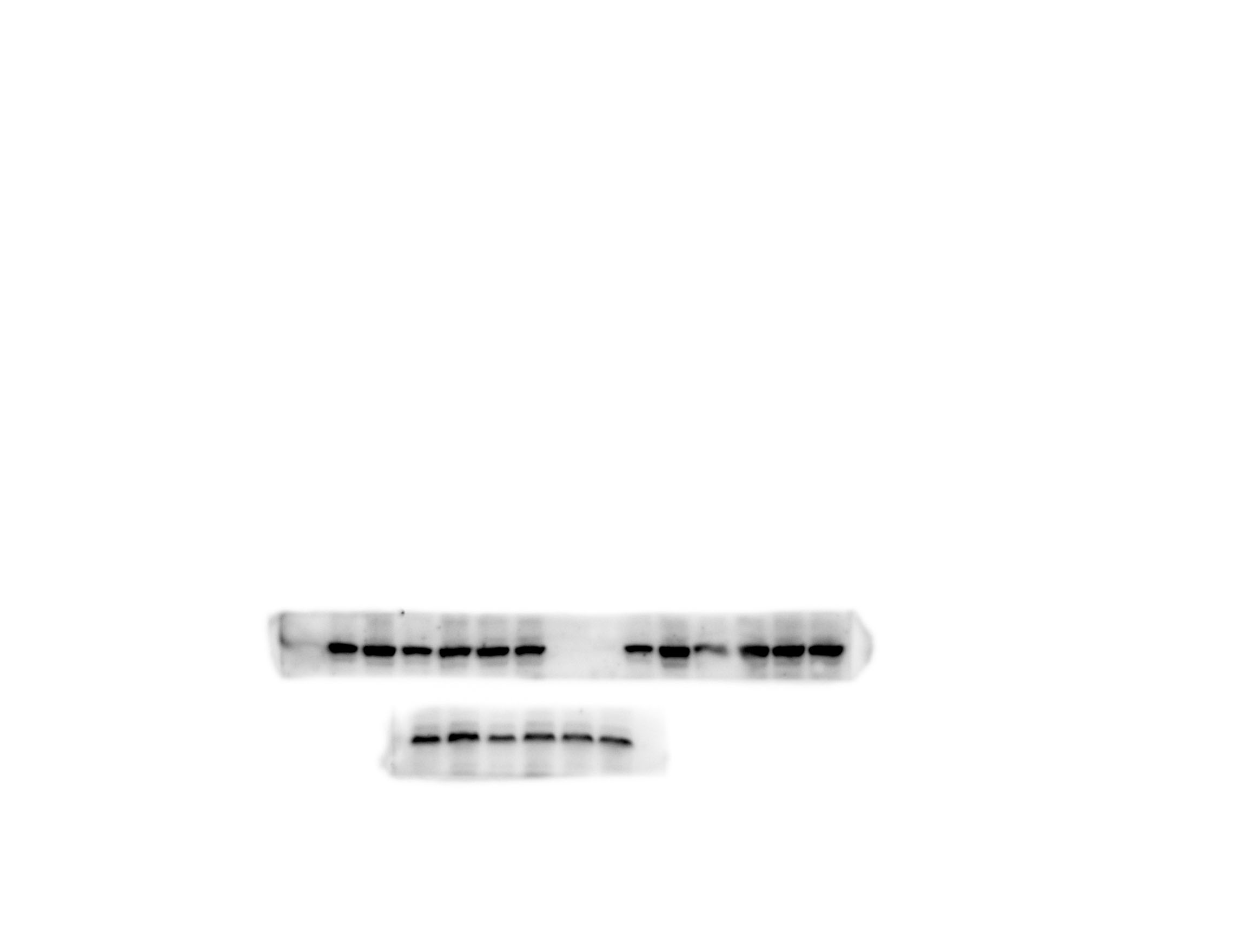

Supplement: Supplementary file 1 [file DataSheet1.ZIP › Original data/Supplementary figure 5-original data/AKT+P-AKT/P-AKT-1,2,3.jpg]

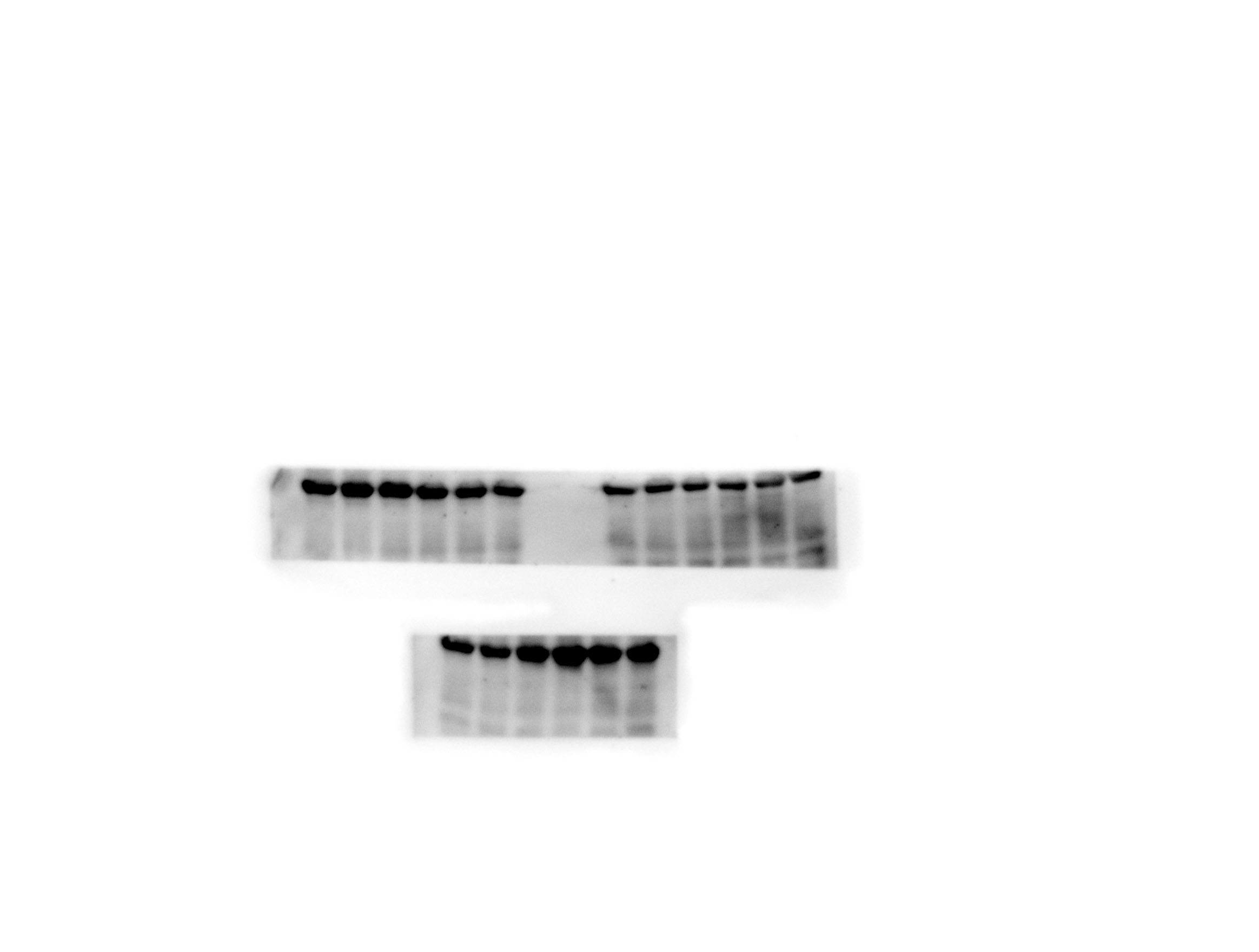

Supplement: Supplementary file 1 [file DataSheet1.ZIP › Original data/Supplementary figure 5-original data/AKT+P-AKT/β-Actin-AKT+P-AKT-1,2,3.jpg]

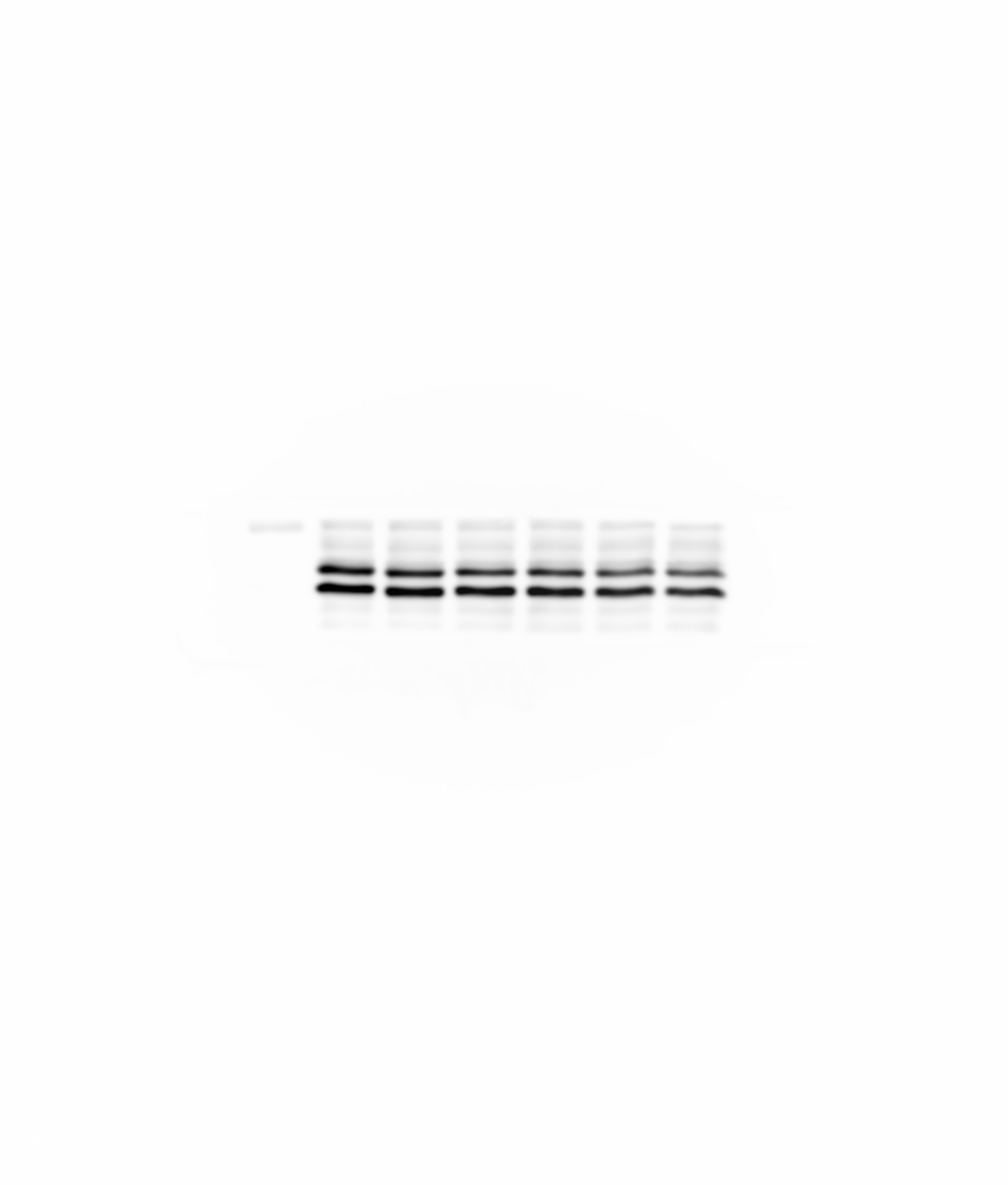

Supplement: Supplementary file 1 [file DataSheet1.ZIP › Original data/Supplementary figure 5-original data/ERK+P-ERK/ERK-1.jpg]

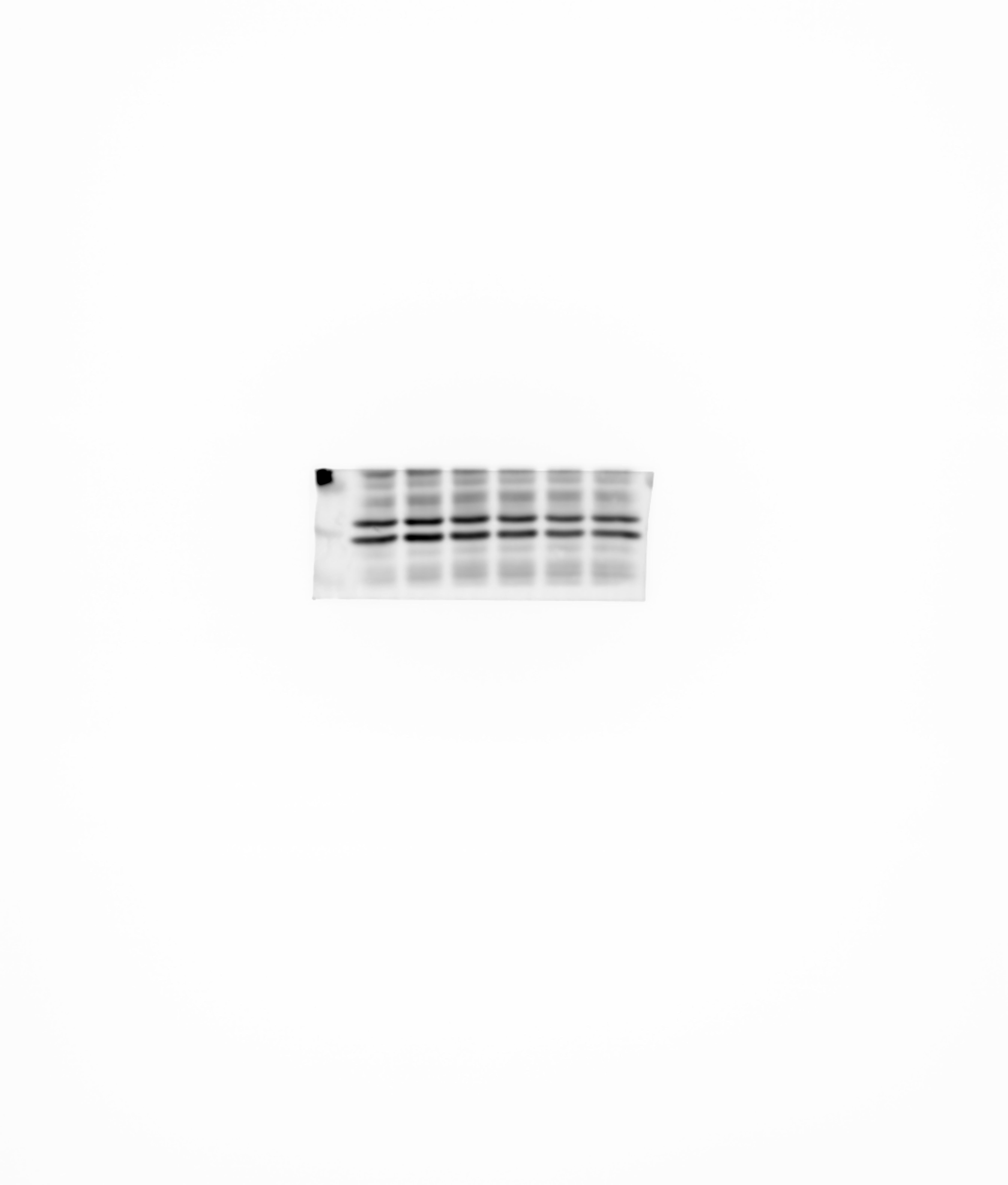

Supplement: Supplementary file 1 [file DataSheet1.ZIP › Original data/Supplementary figure 5-original data/ERK+P-ERK/ERK-2.jpg]

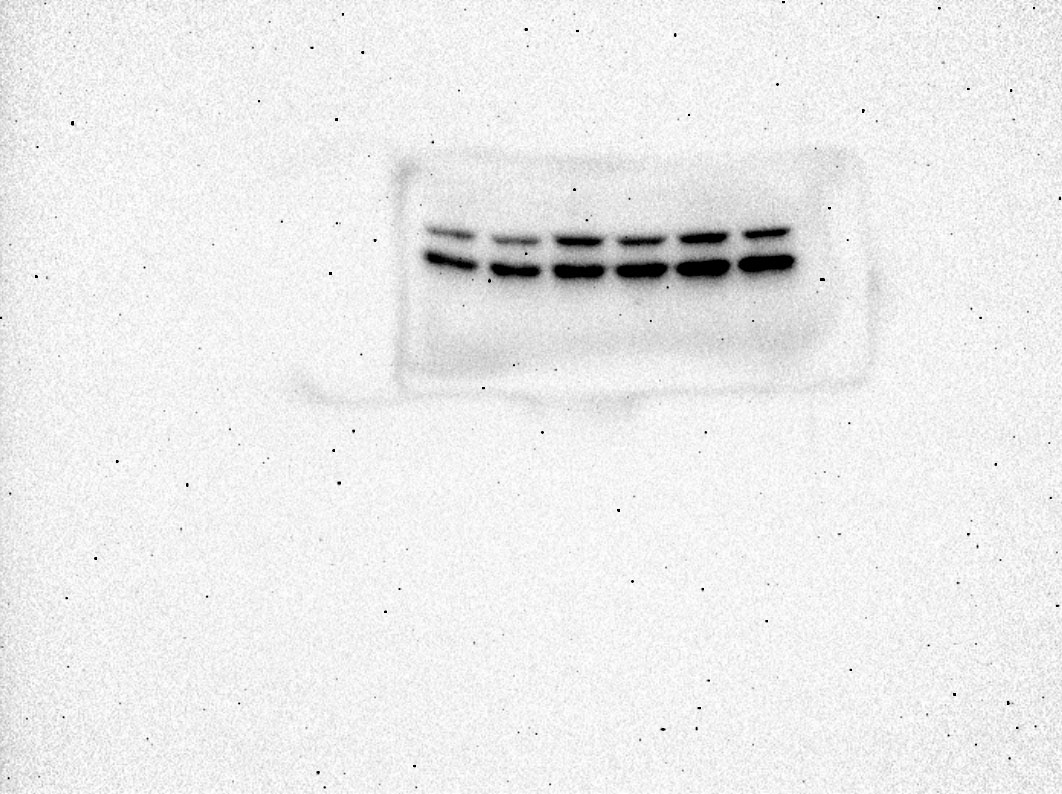

Supplement: Supplementary file 1 [file DataSheet1.ZIP › Original data/Supplementary figure 5-original data/ERK+P-ERK/ERK-3.jpg]

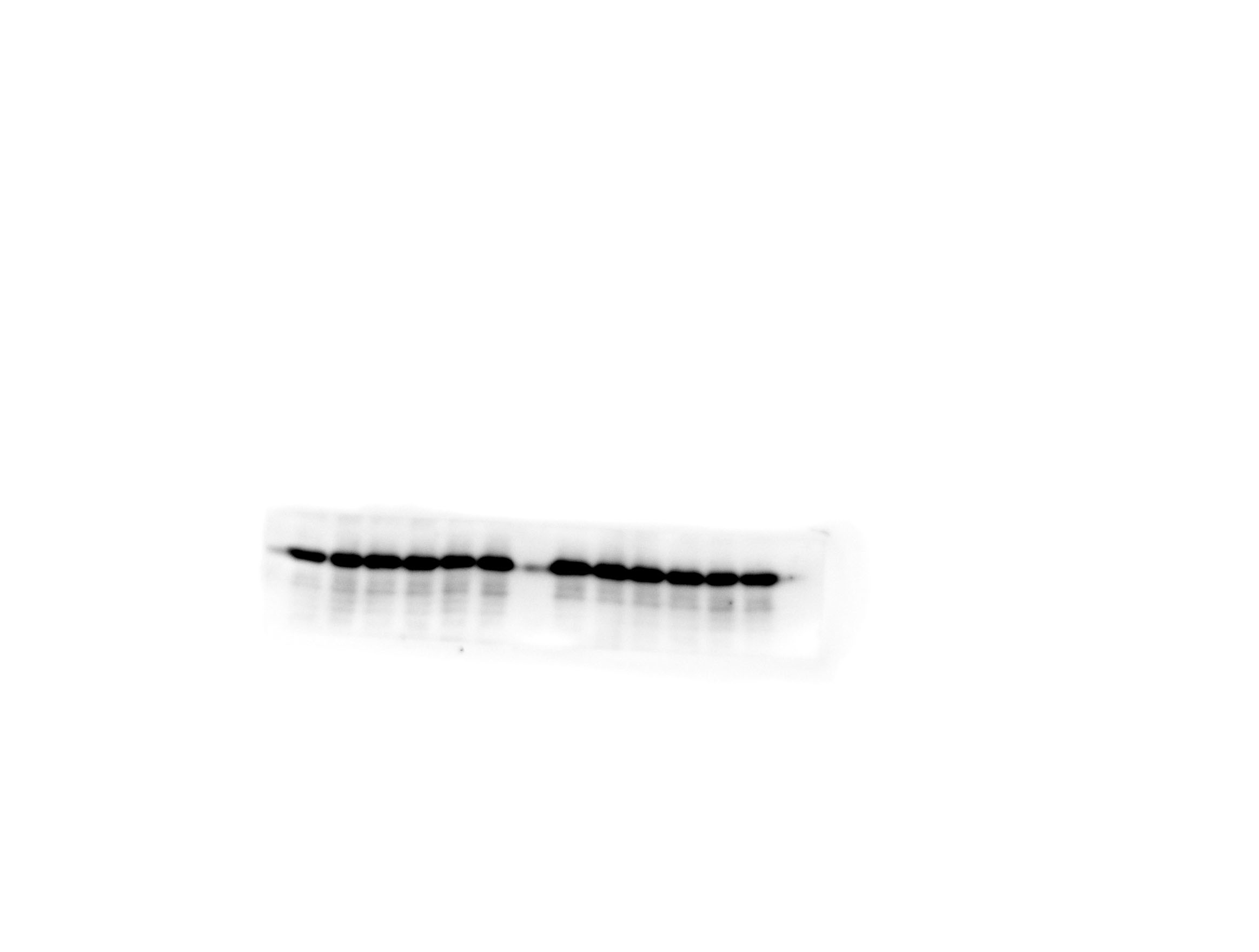

Supplement: Supplementary file 1 [file DataSheet1.ZIP › Original data/Supplementary figure 5-original data/ERK+P-ERK/GAPDH-ERK-1,2.jpg]

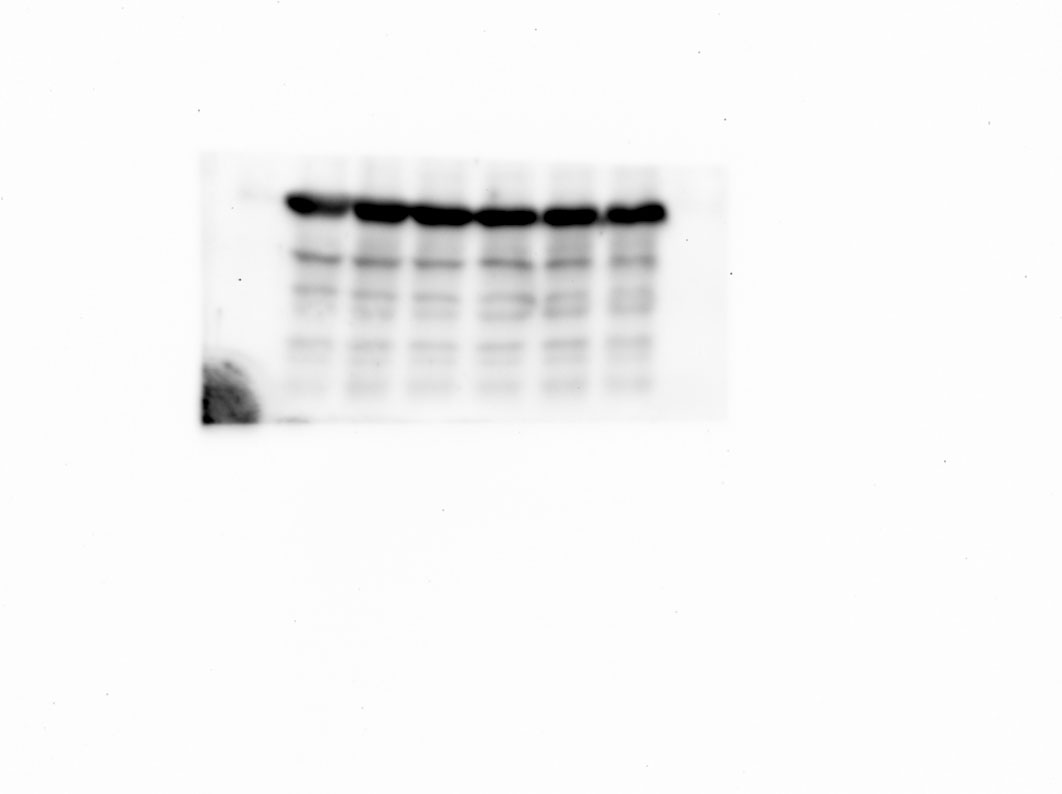

Supplement: Supplementary file 1 [file DataSheet1.ZIP › Original data/Supplementary figure 5-original data/ERK+P-ERK/GAPDH-ERK-3.jpg]

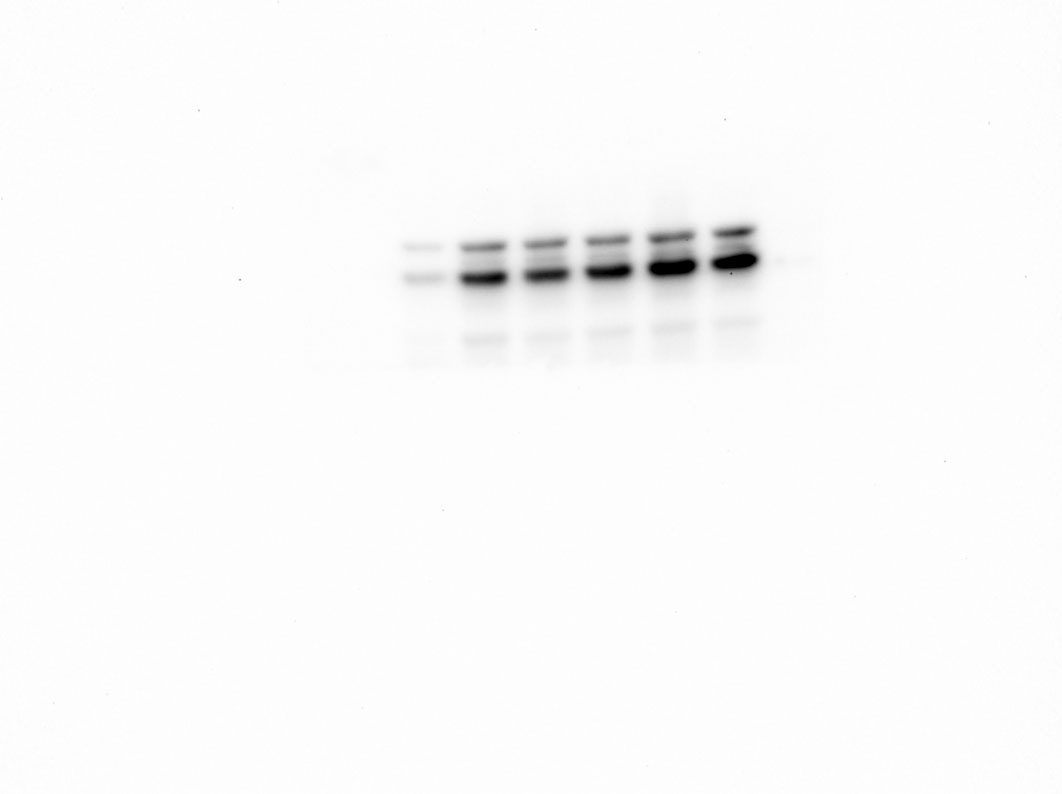

Supplement: Supplementary file 1 [file DataSheet1.ZIP › Original data/Supplementary figure 5-original data/ERK+P-ERK/P-ERK-1.jpg]

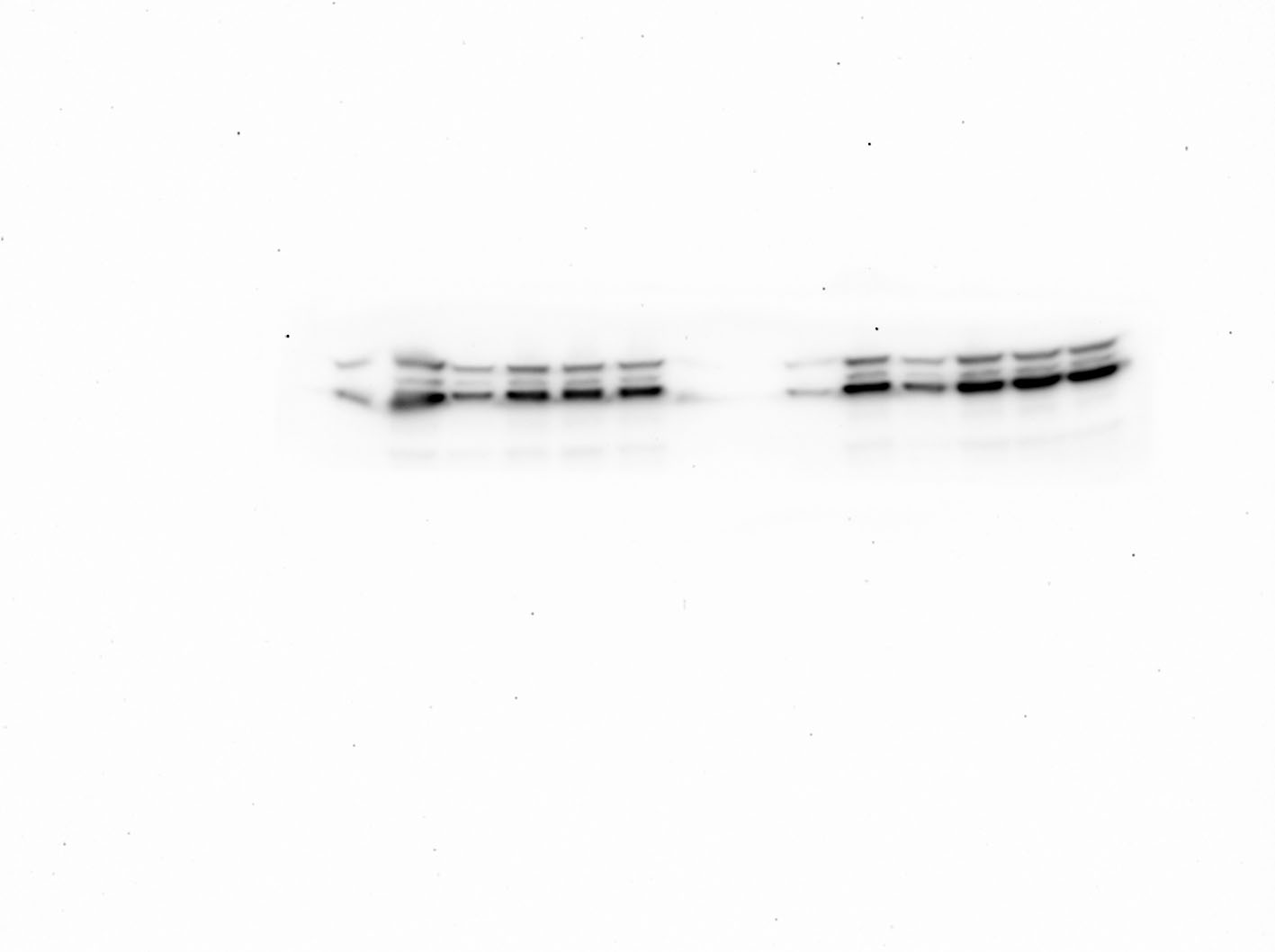

Supplement: Supplementary file 1 [file DataSheet1.ZIP › Original data/Supplementary figure 5-original data/ERK+P-ERK/P-ERK-2,3.jpg]

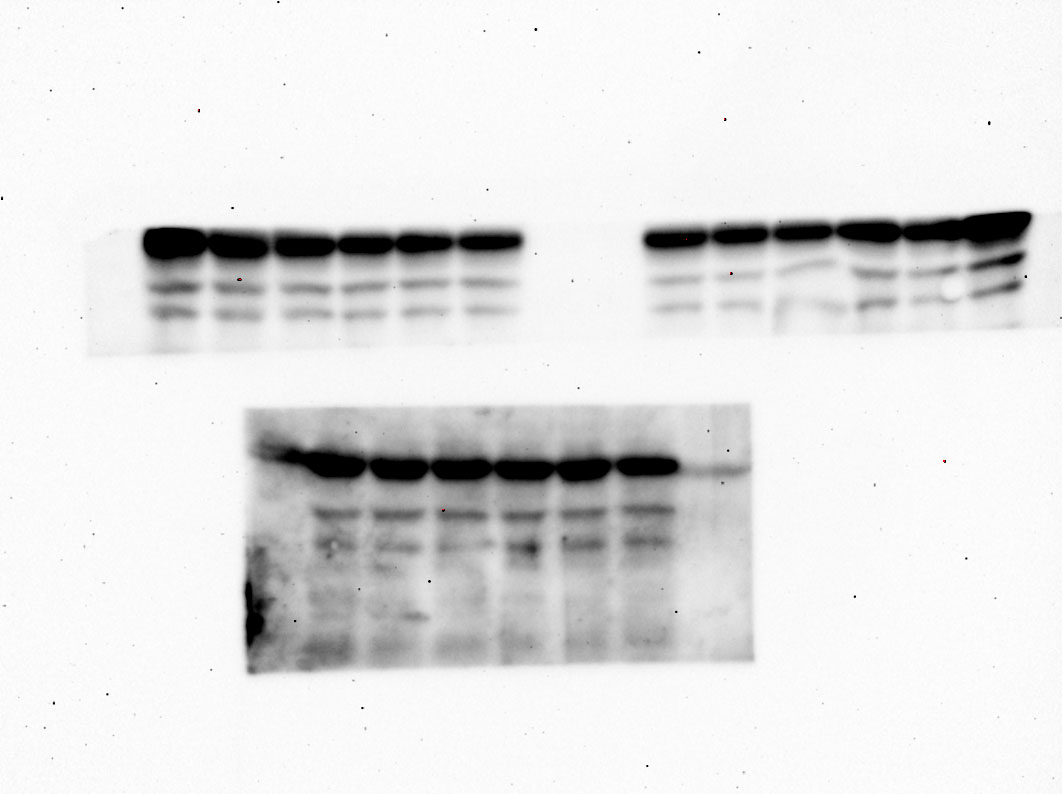

Supplement: Supplementary file 1 [file DataSheet1.ZIP › Original data/Supplementary figure 5-original data/JNK+P-JNK/GAPDH-1,2,3.jpg]

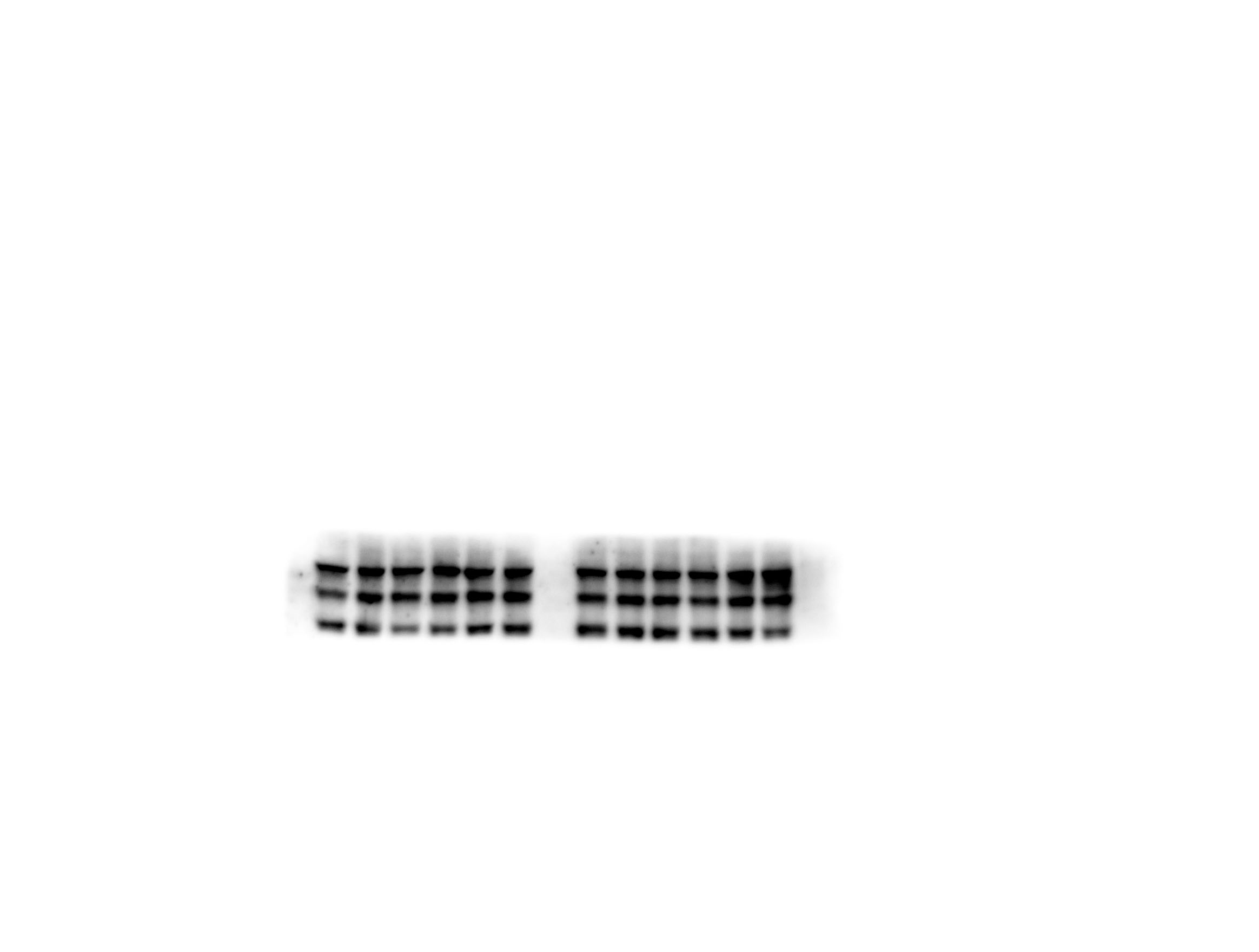

Supplement: Supplementary file 1 [file DataSheet1.ZIP › Original data/Supplementary figure 5-original data/JNK+P-JNK/JNK-1,2.jpg]

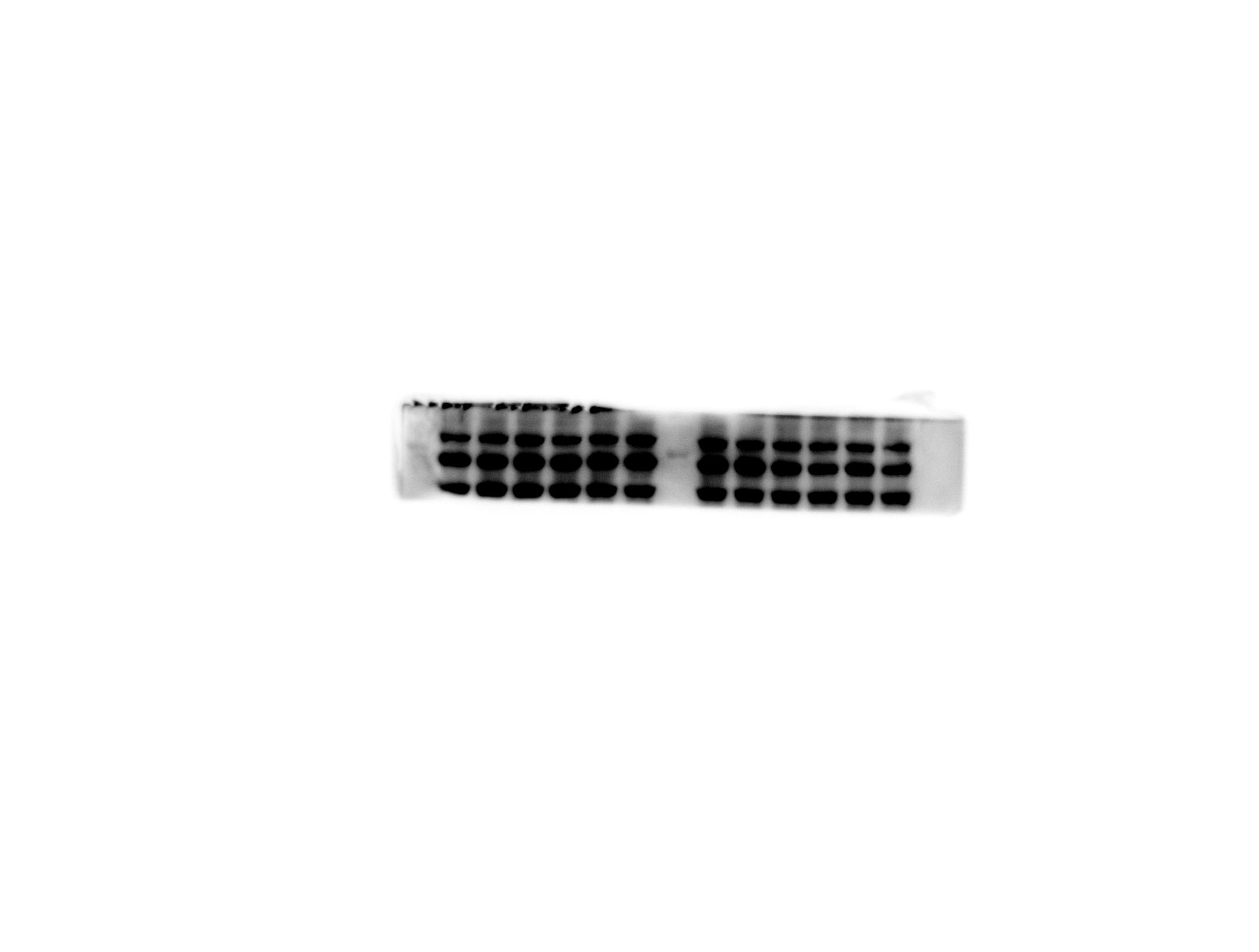

Supplement: Supplementary file 1 [file DataSheet1.ZIP › Original data/Supplementary figure 5-original data/JNK+P-JNK/JNK-3.jpg]

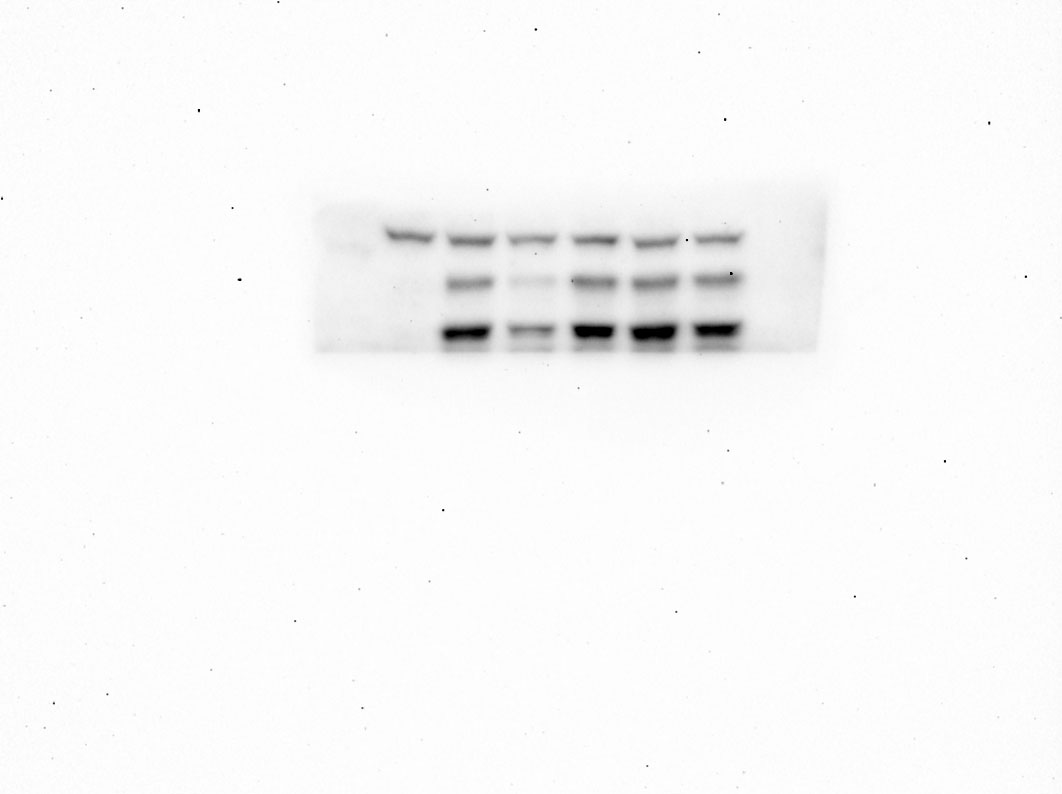

Supplement: Supplementary file 1 [file DataSheet1.ZIP › Original data/Supplementary figure 5-original data/JNK+P-JNK/P-JNK-1.jpg]

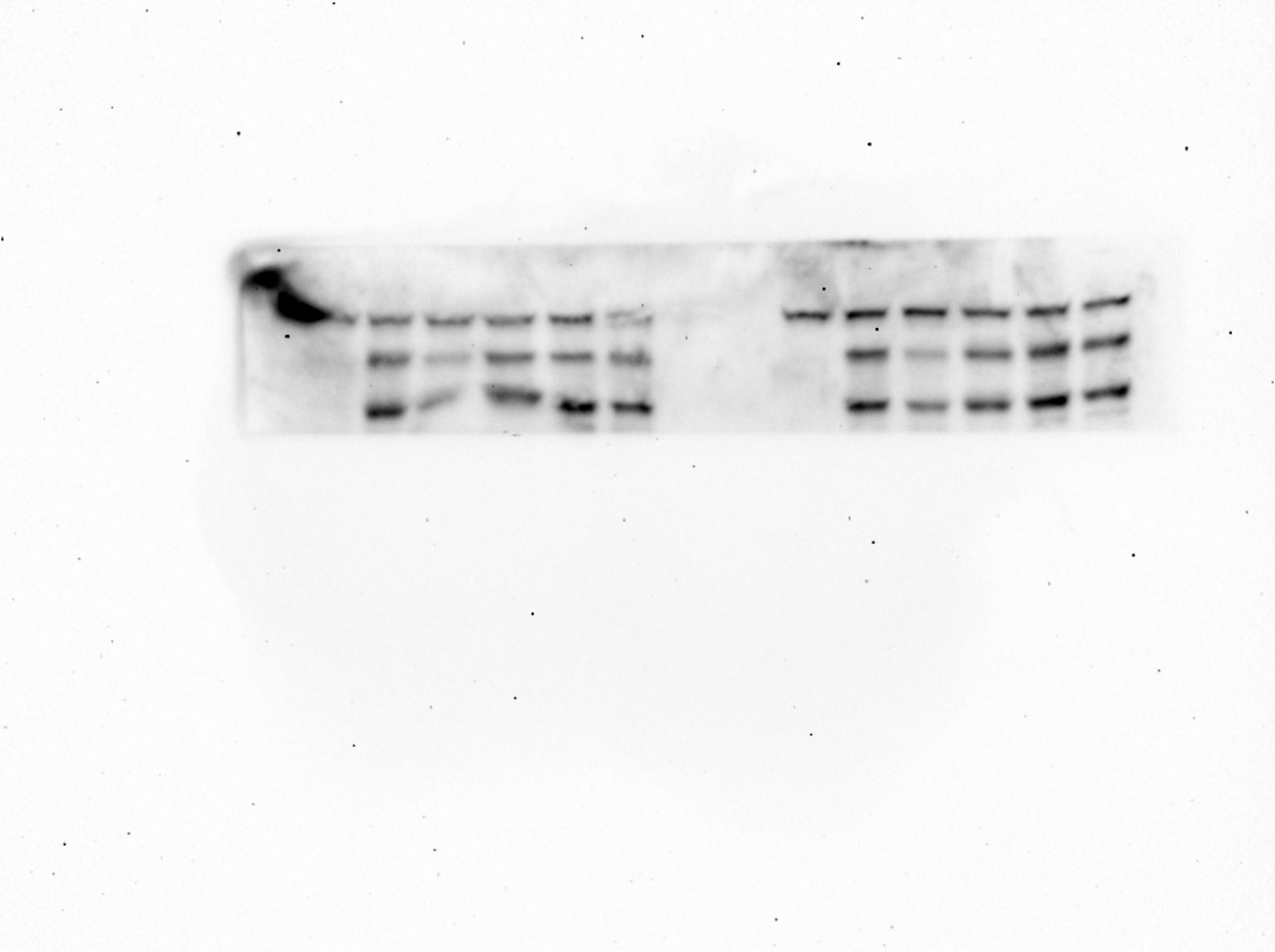

Supplement: Supplementary file 1 [file DataSheet1.ZIP › Original data/Supplementary figure 5-original data/JNK+P-JNK/P-JNK-2,3.jpg]

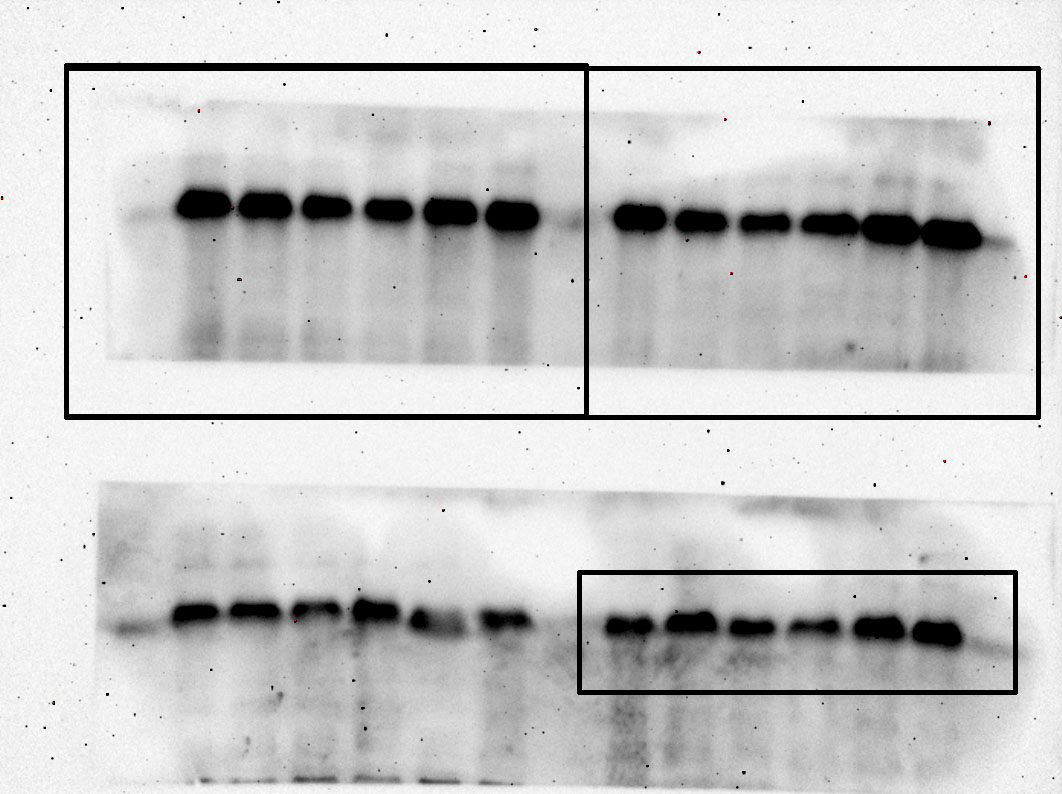

Supplement: Supplementary file 1 [file DataSheet1.ZIP › Original data/Supplementary figure 5-original data/P38+P-P38/GAPDH-P38-1,2,3.jpg]

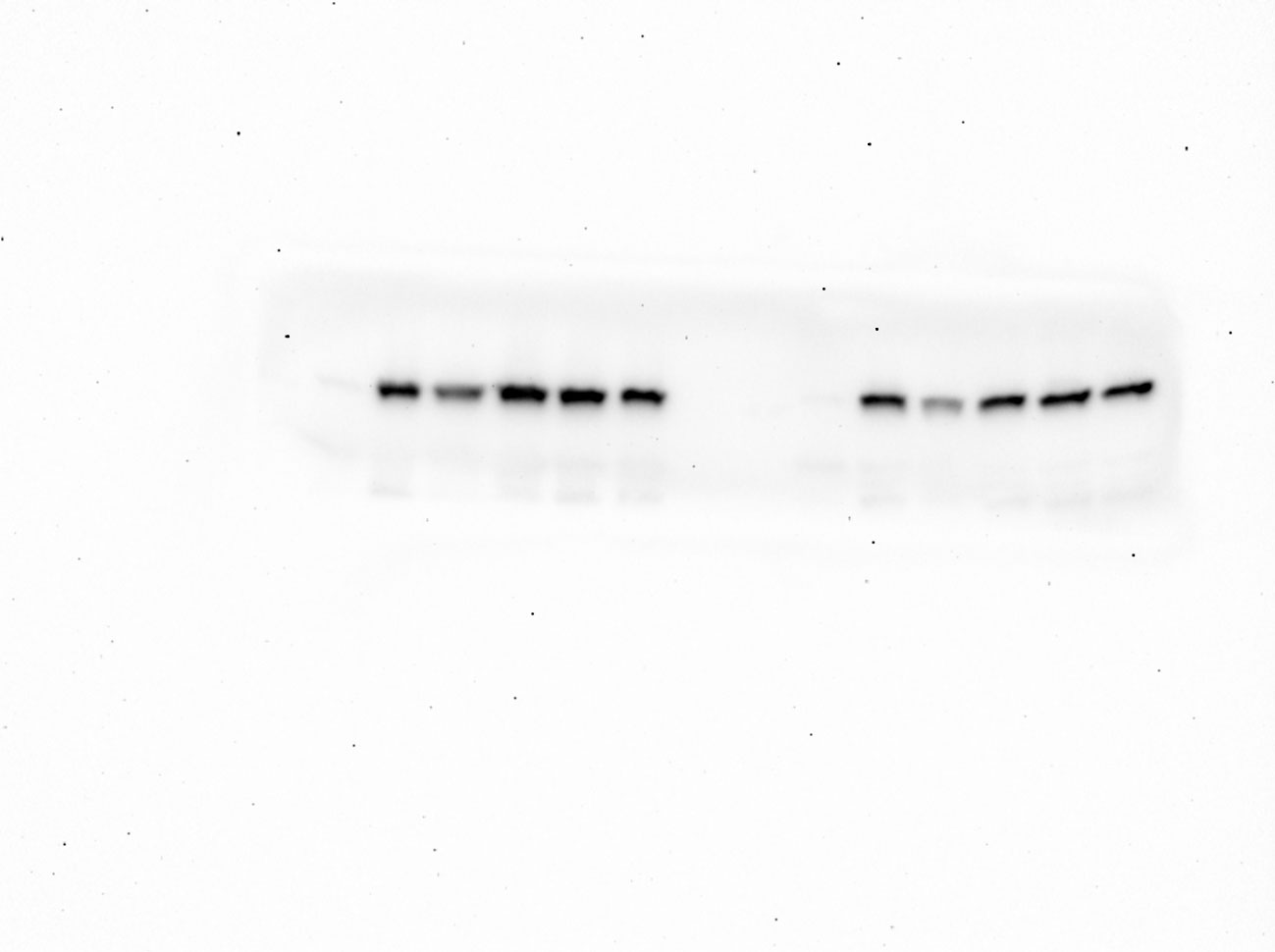

Supplement: Supplementary file 1 [file DataSheet1.ZIP › Original data/Supplementary figure 5-original data/P38+P-P38/P-P38-1,2.jpg]

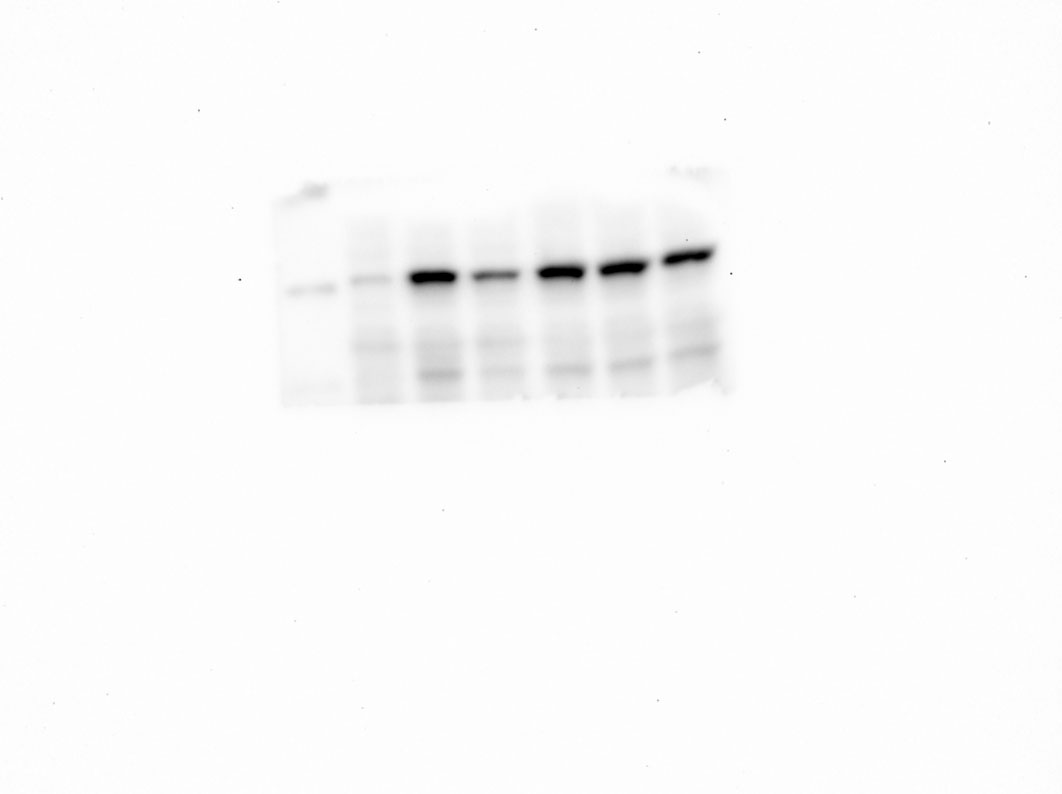

Supplement: Supplementary file 1 [file DataSheet1.ZIP › Original data/Supplementary figure 5-original data/P38+P-P38/P-P38-3.jpg]

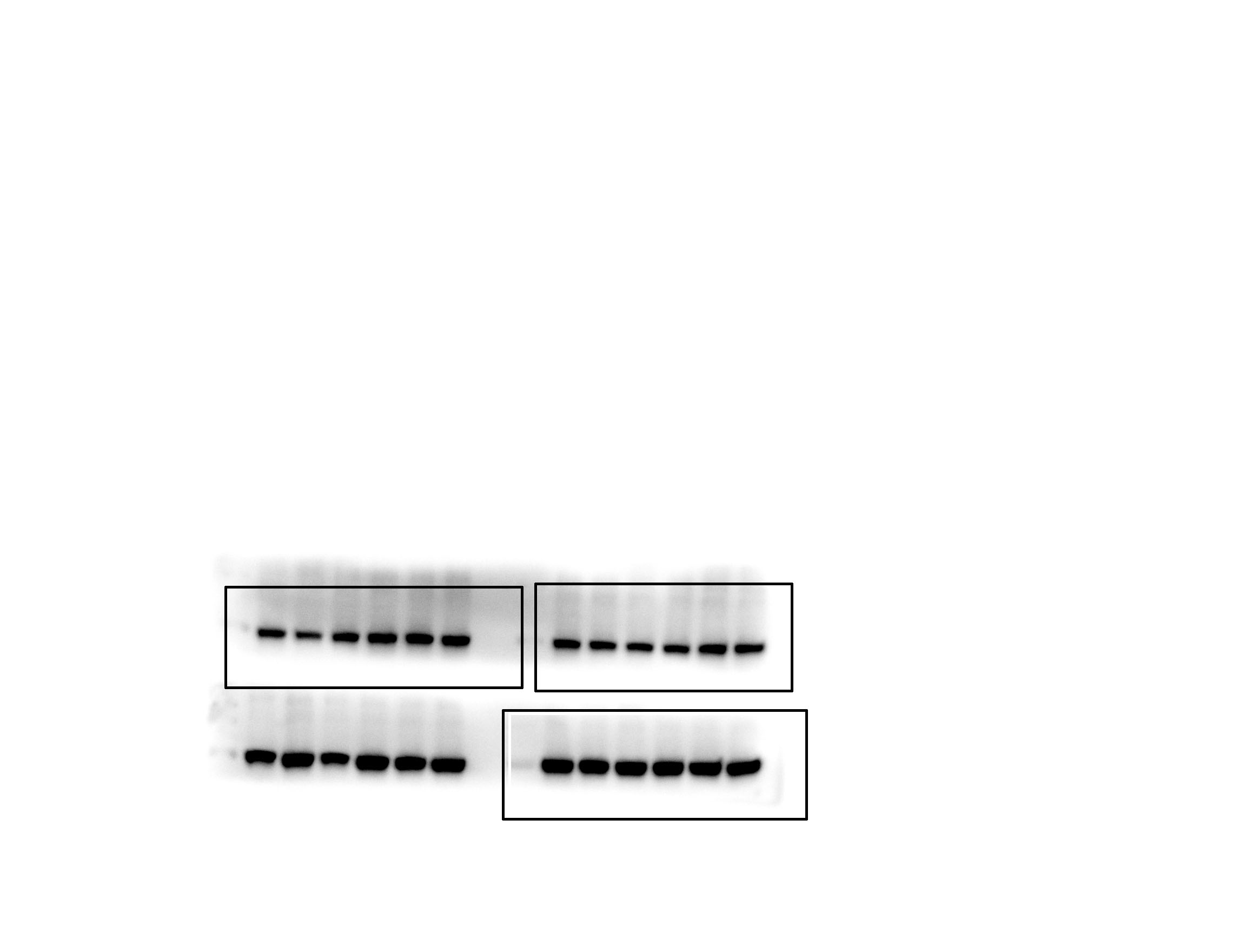

Supplement: Supplementary file 1 [file DataSheet1.ZIP › Original data/Supplementary figure 5-original data/P38+P-P38/P38-1,2,3.jpg]

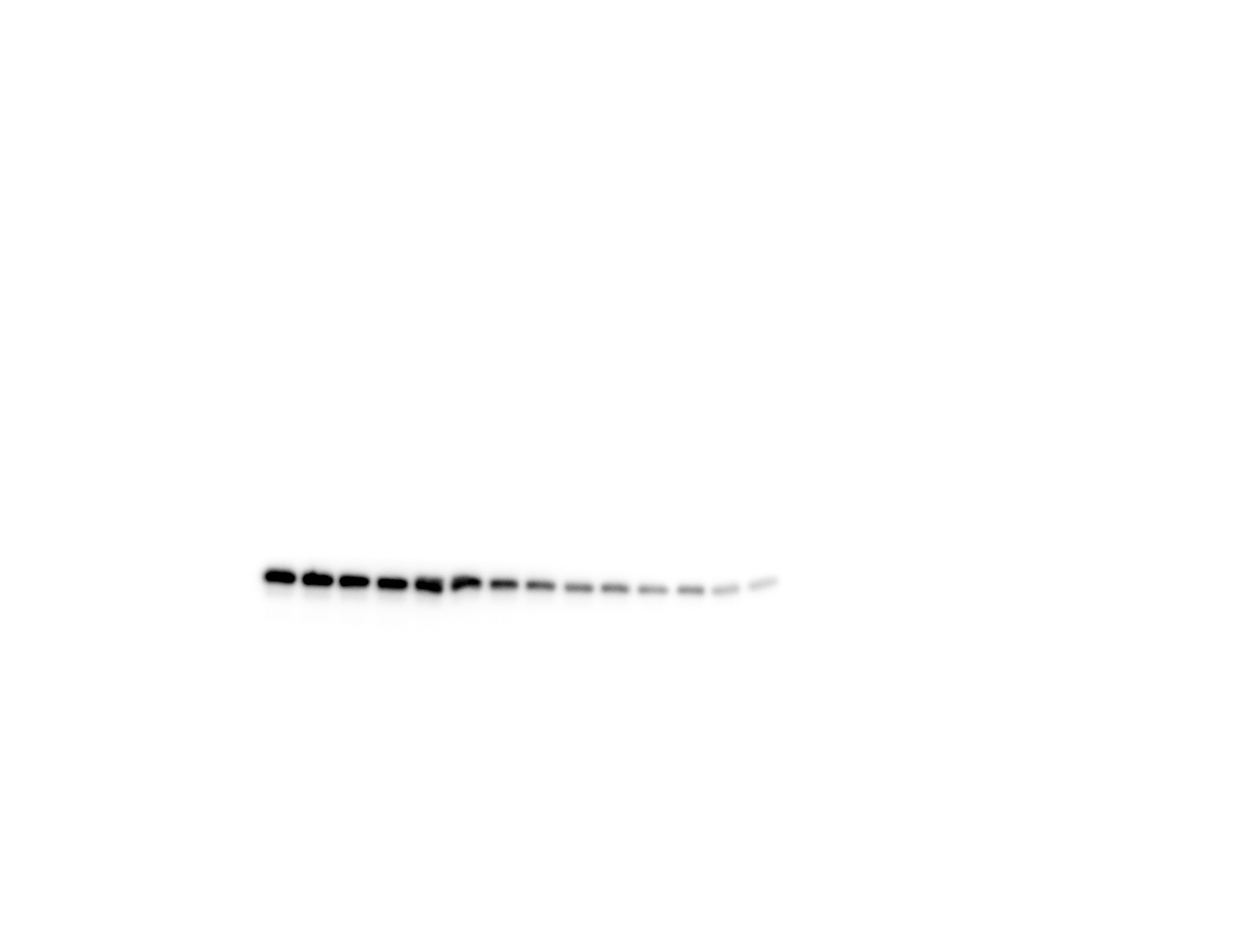

Supplement: Supplementary file 1 [file DataSheet1.ZIP › Original data/Supplementary figure 6-original data/IKBα-BVA-CETSA-1.tif]

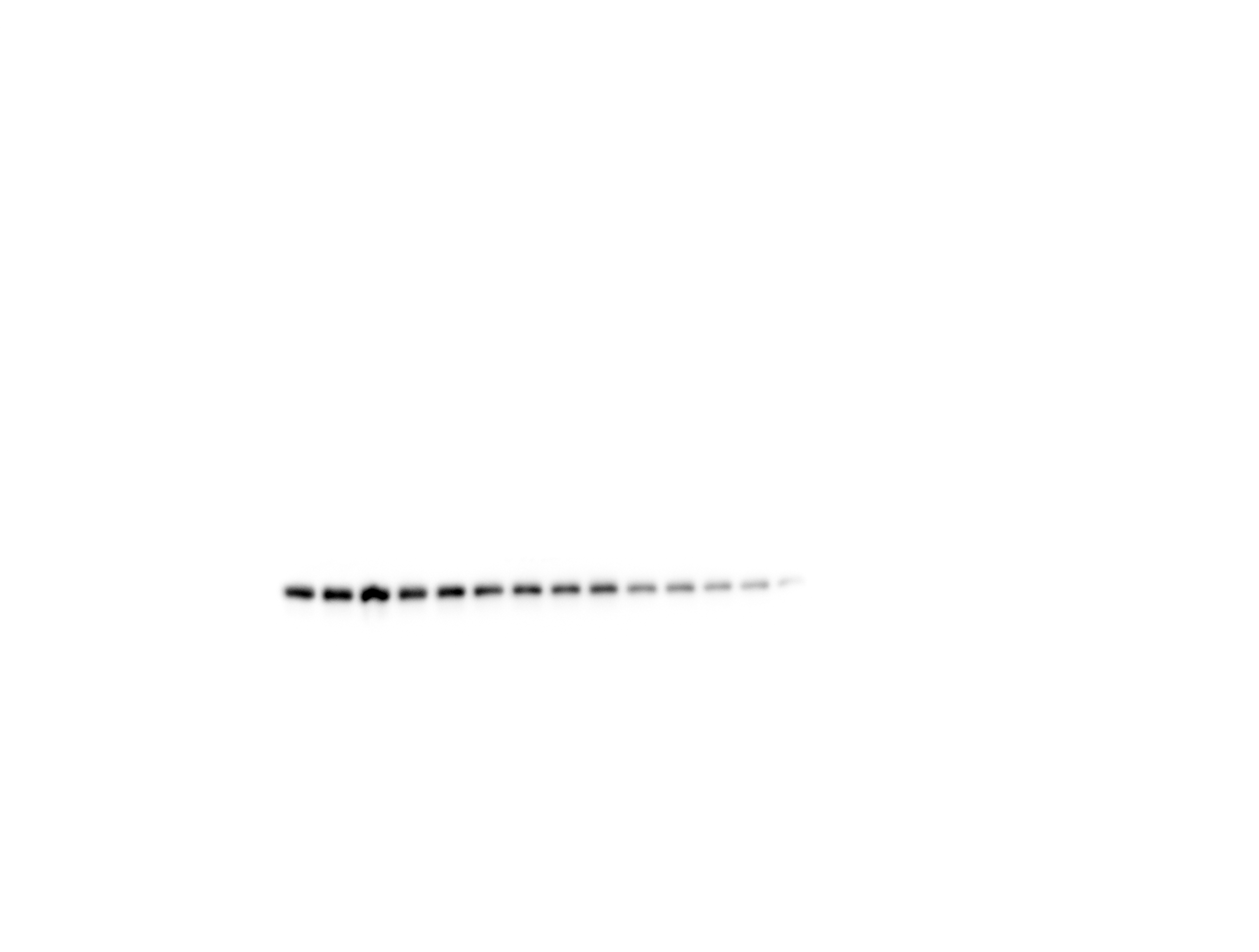

Supplement: Supplementary file 1 [file DataSheet1.ZIP › Original data/Supplementary figure 6-original data/IKBα-BVA-CETSA-2.tif]

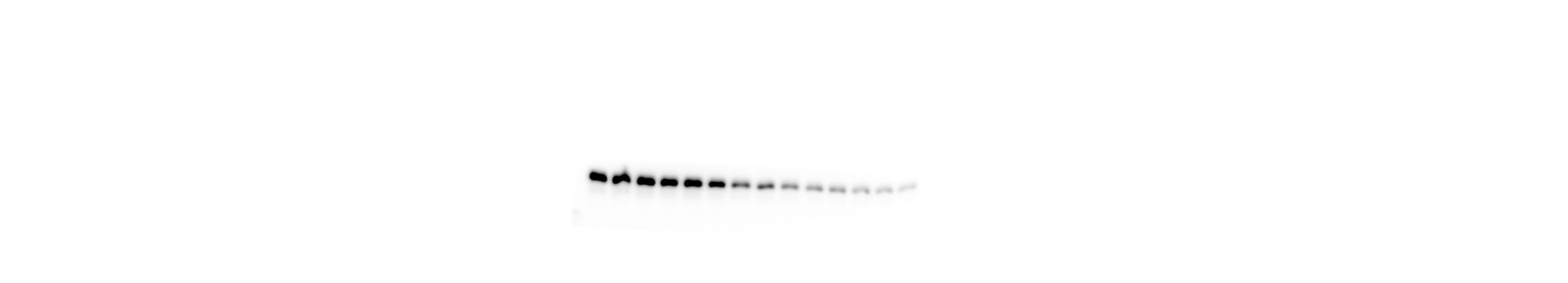

Supplement: Supplementary file 1 [file DataSheet1.ZIP › Original data/Supplementary figure 6-original data/IκBα-CETSA-BVA-3.jpg]

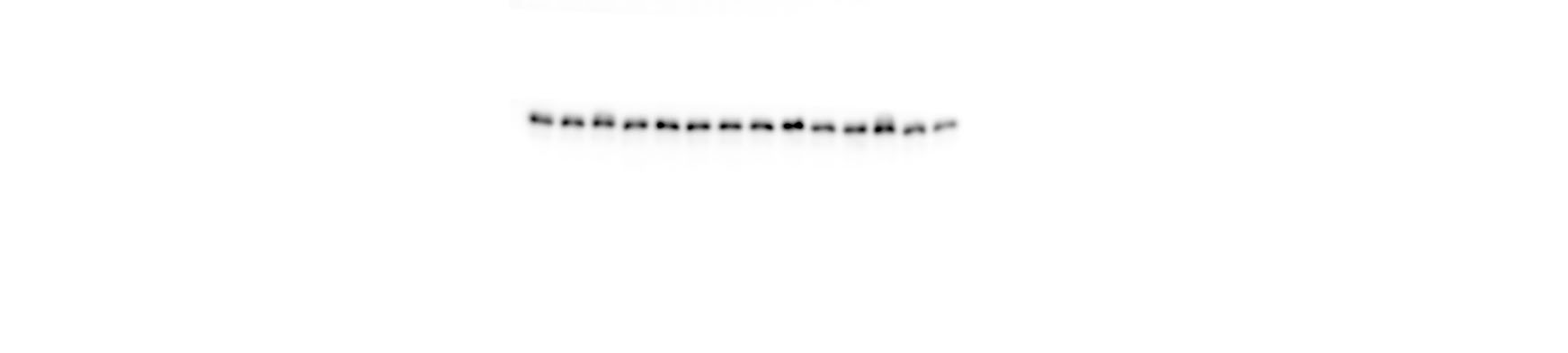

Supplement: Supplementary file 1 [file DataSheet1.ZIP › Original data/Supplementary figure 6-original data/IκBα-CETSA-BVA-unheated.jpg]

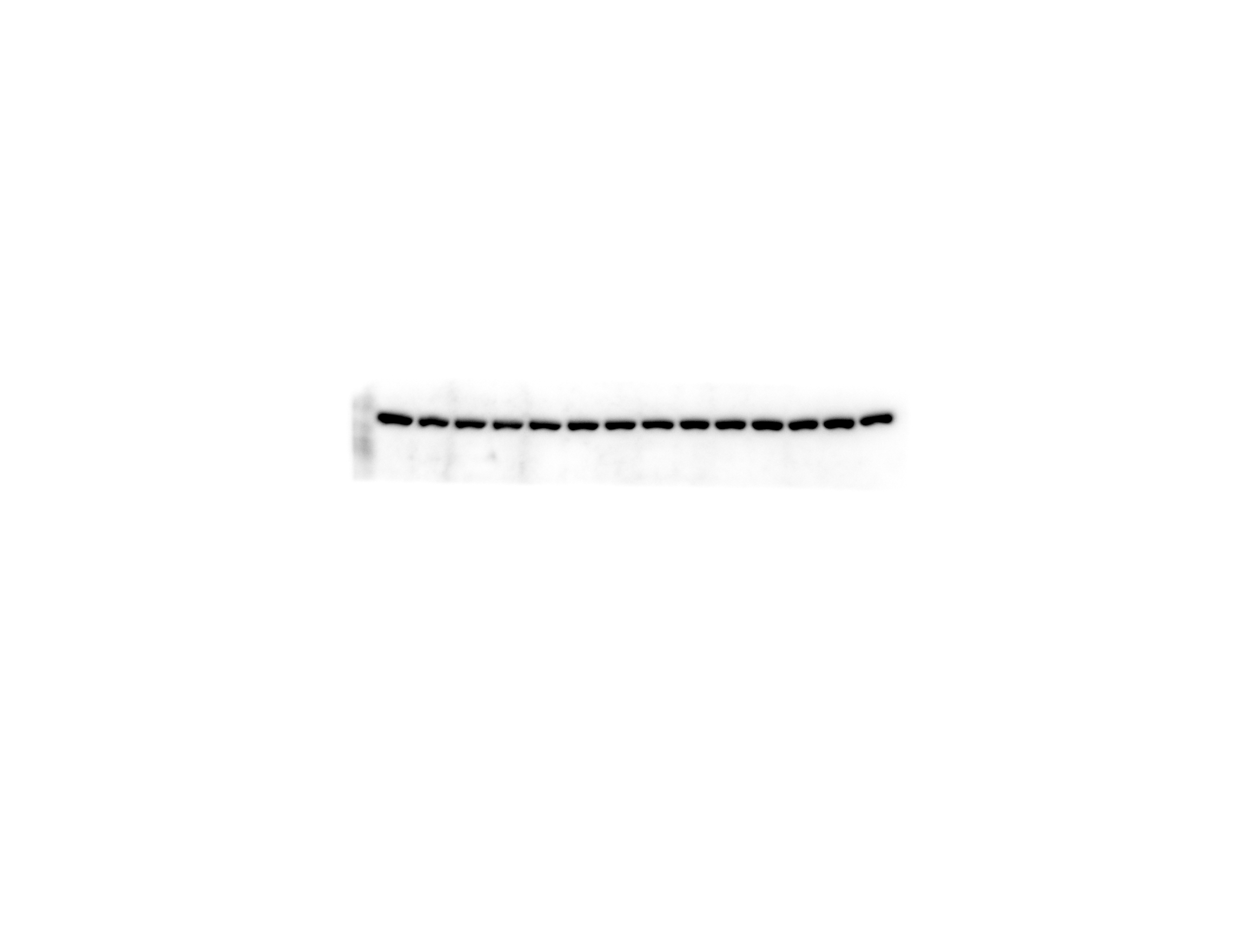

Supplement: Supplementary file 1 [file DataSheet1.ZIP › Original data/Supplementary figure 6-original data/Tubulin(unheated)-CETSA-BVA.tif]

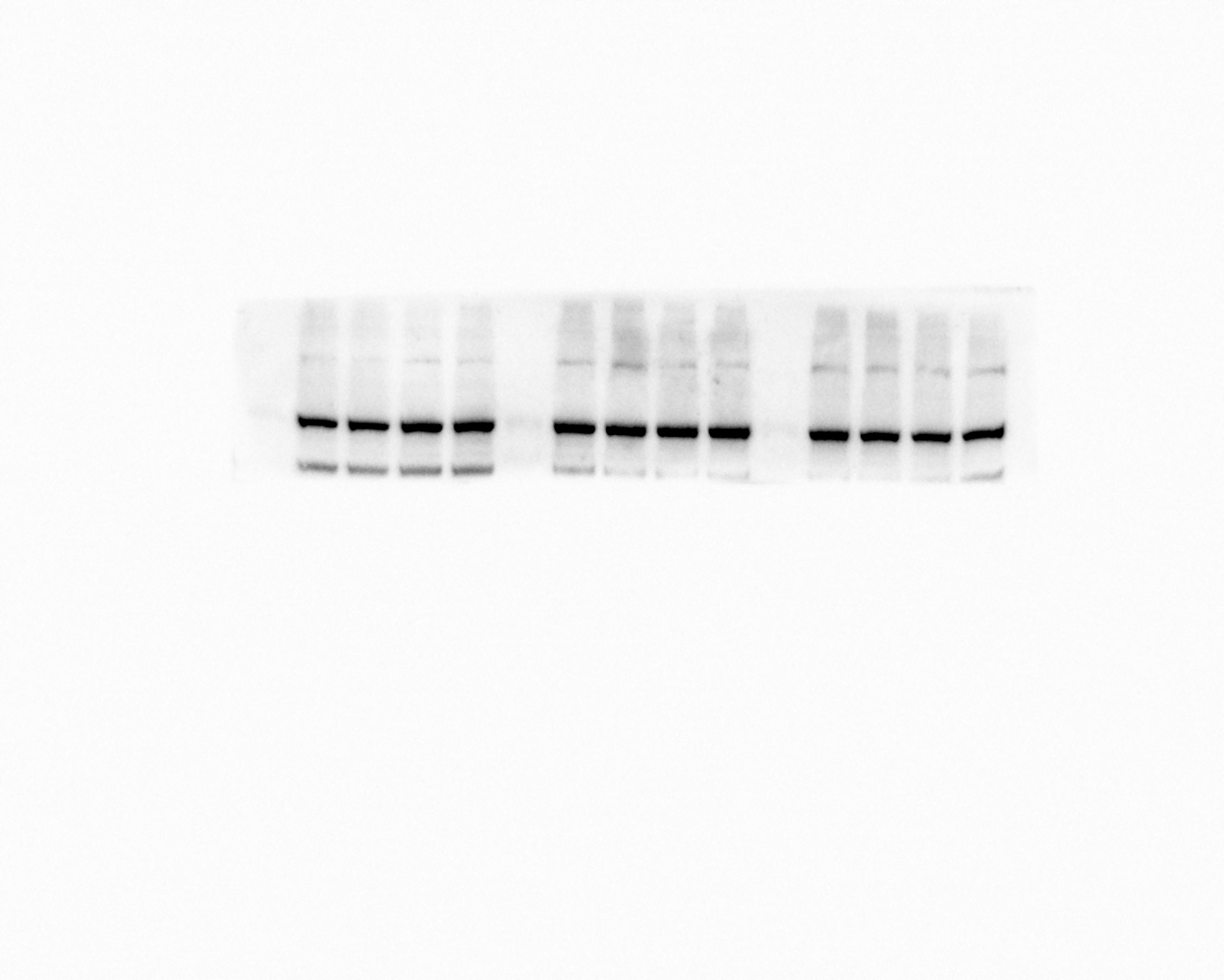

Supplement: Supplementary file 1 [file DataSheet1.ZIP › Original data/Supplementary figure 7-original data/IKK-BVA-ALI-Mice-1,2,3.tif]

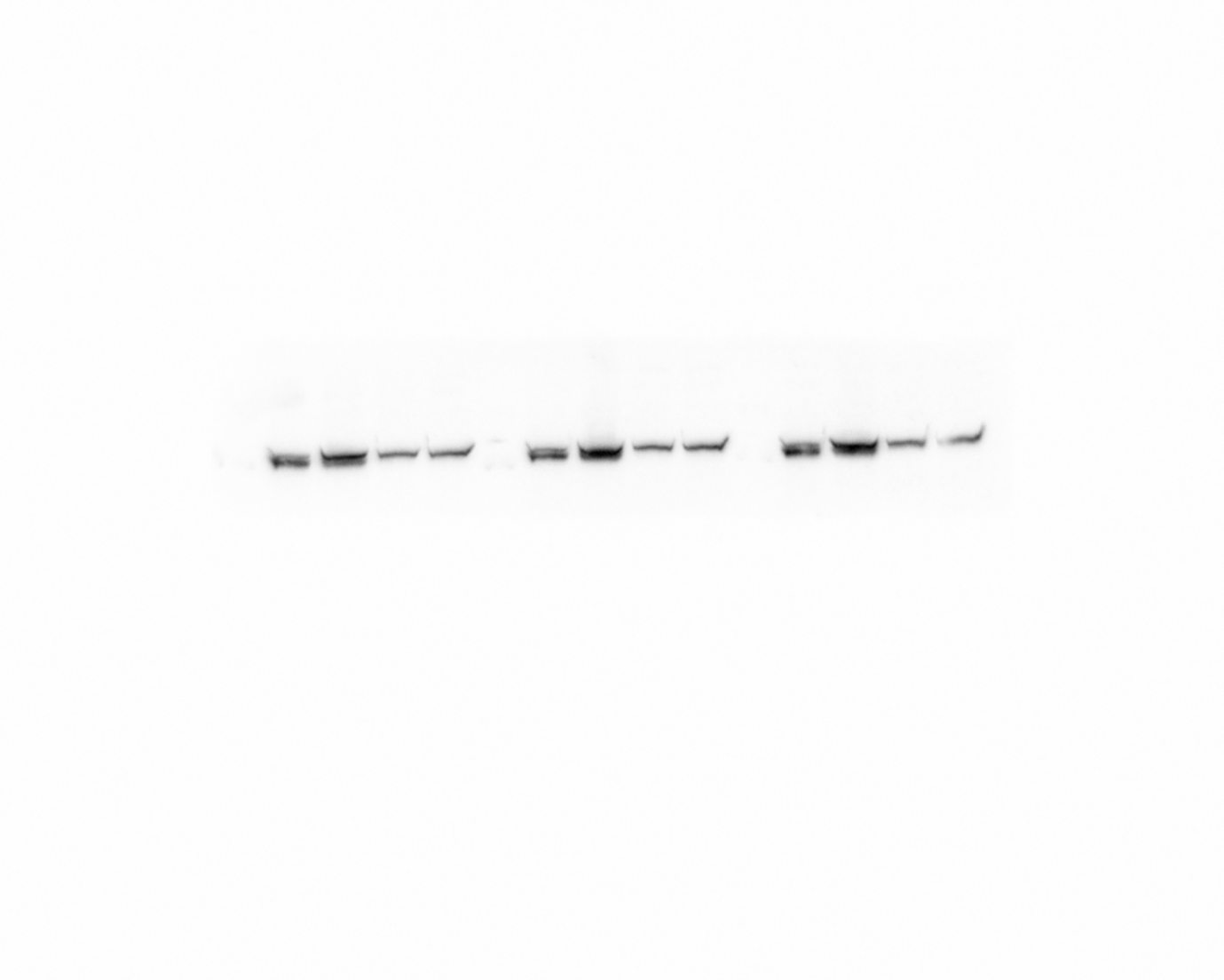

Supplement: Supplementary file 1 [file DataSheet1.ZIP › Original data/Supplementary figure 7-original data/p-IKK-BVA-ALI-Mice-1,2,3.tif]

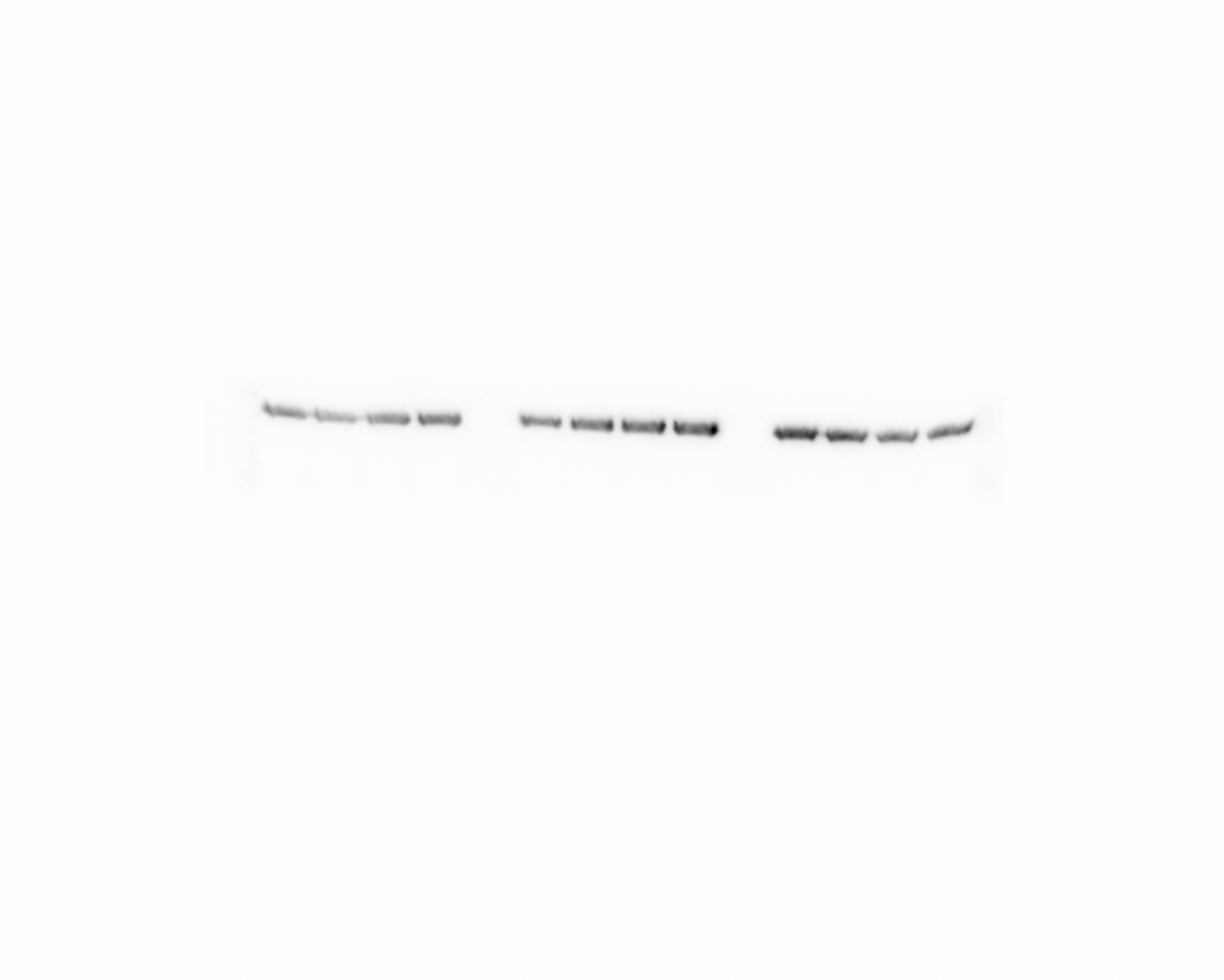

Supplement: Supplementary file 1 [file DataSheet1.ZIP › Original data/Supplementary figure 7-original data/Tubulin-IKK-BVA-ALI-Mice-1,2,3.tif]

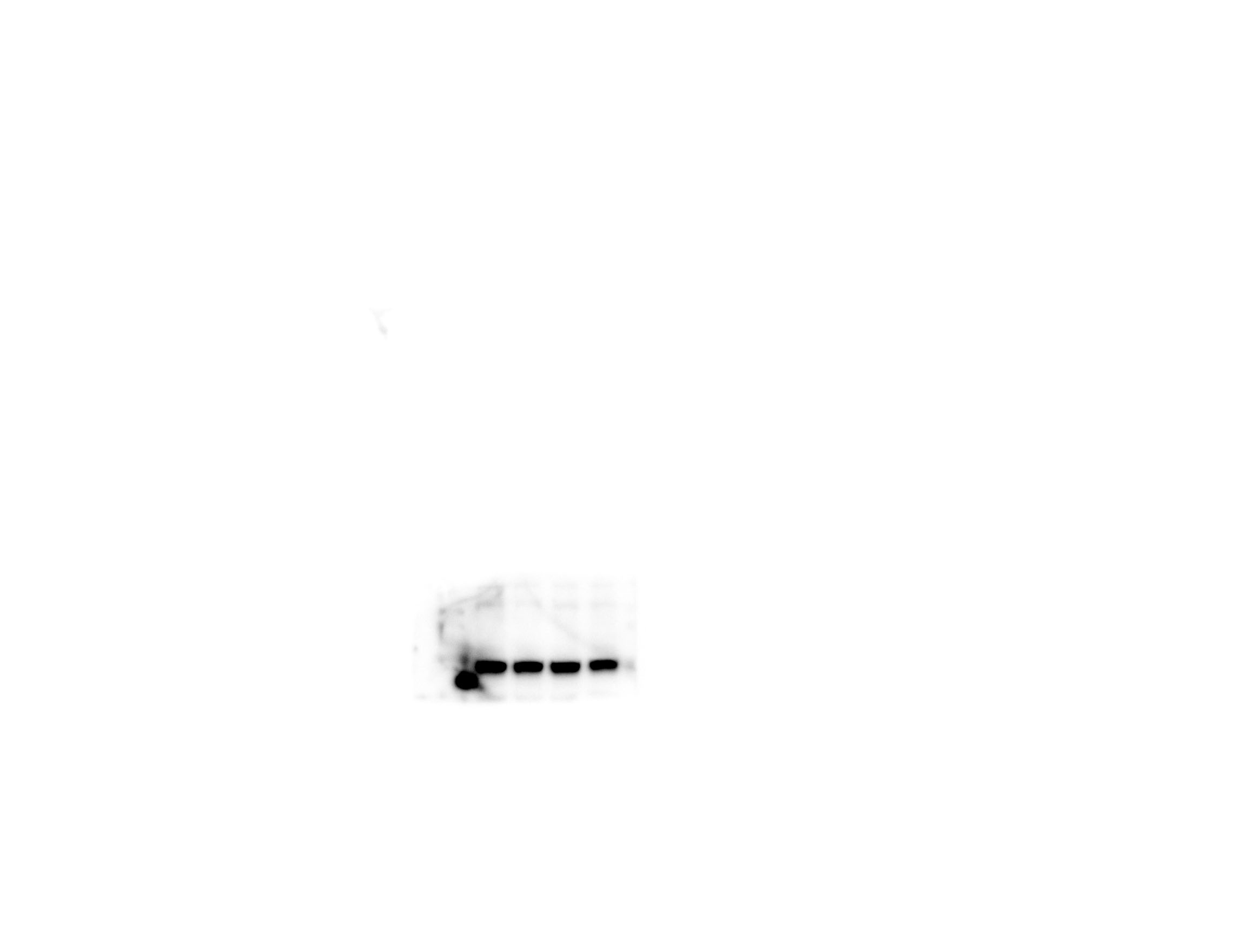

Supplement: Supplementary file 1 [file DataSheet1.ZIP › Original data/Supplementary figure 8-original data/IKK-DTT-1.jpg]

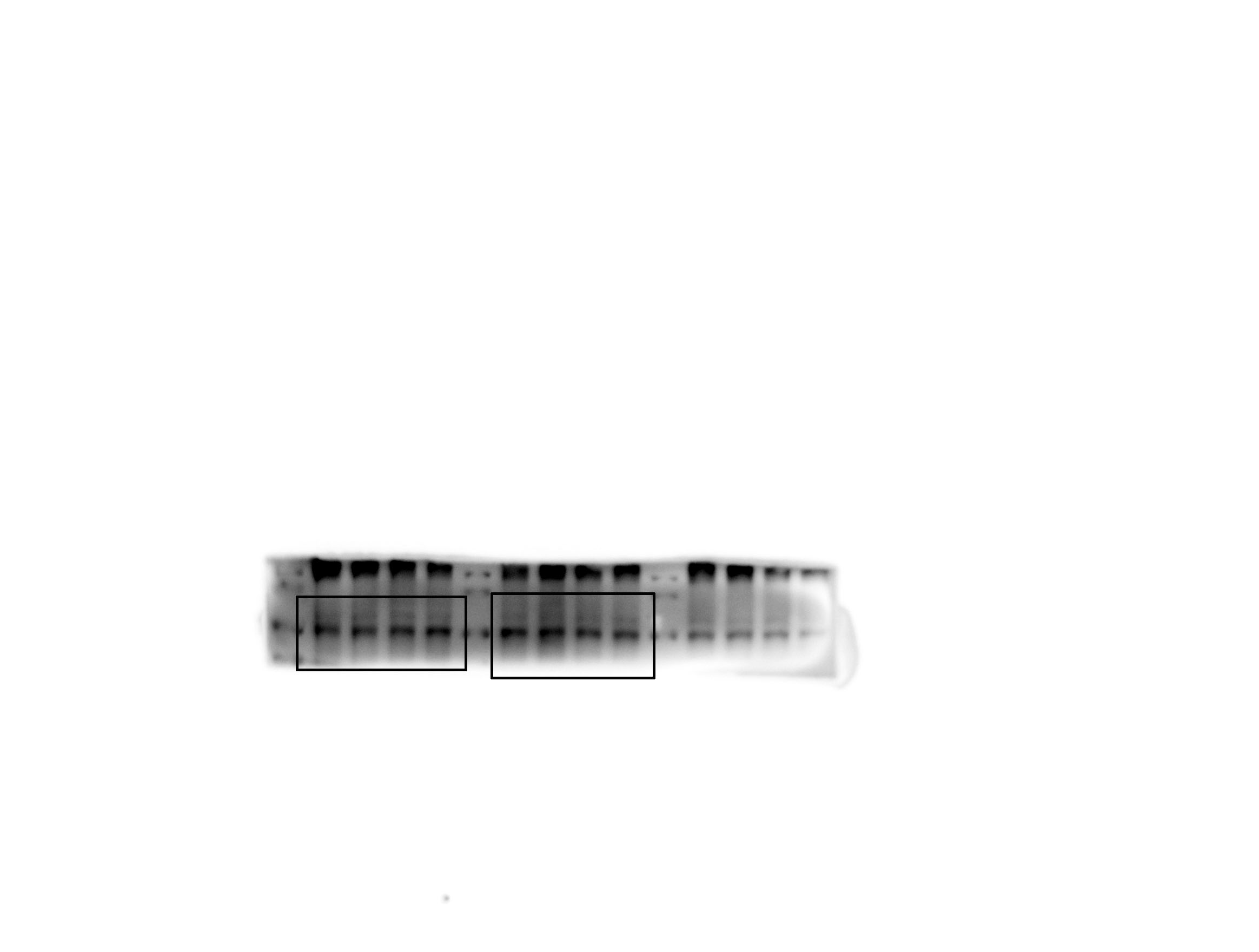

Supplement: Supplementary file 1 [file DataSheet1.ZIP › Original data/Supplementary figure 8-original data/IKK-DTT-2,3.jpg]

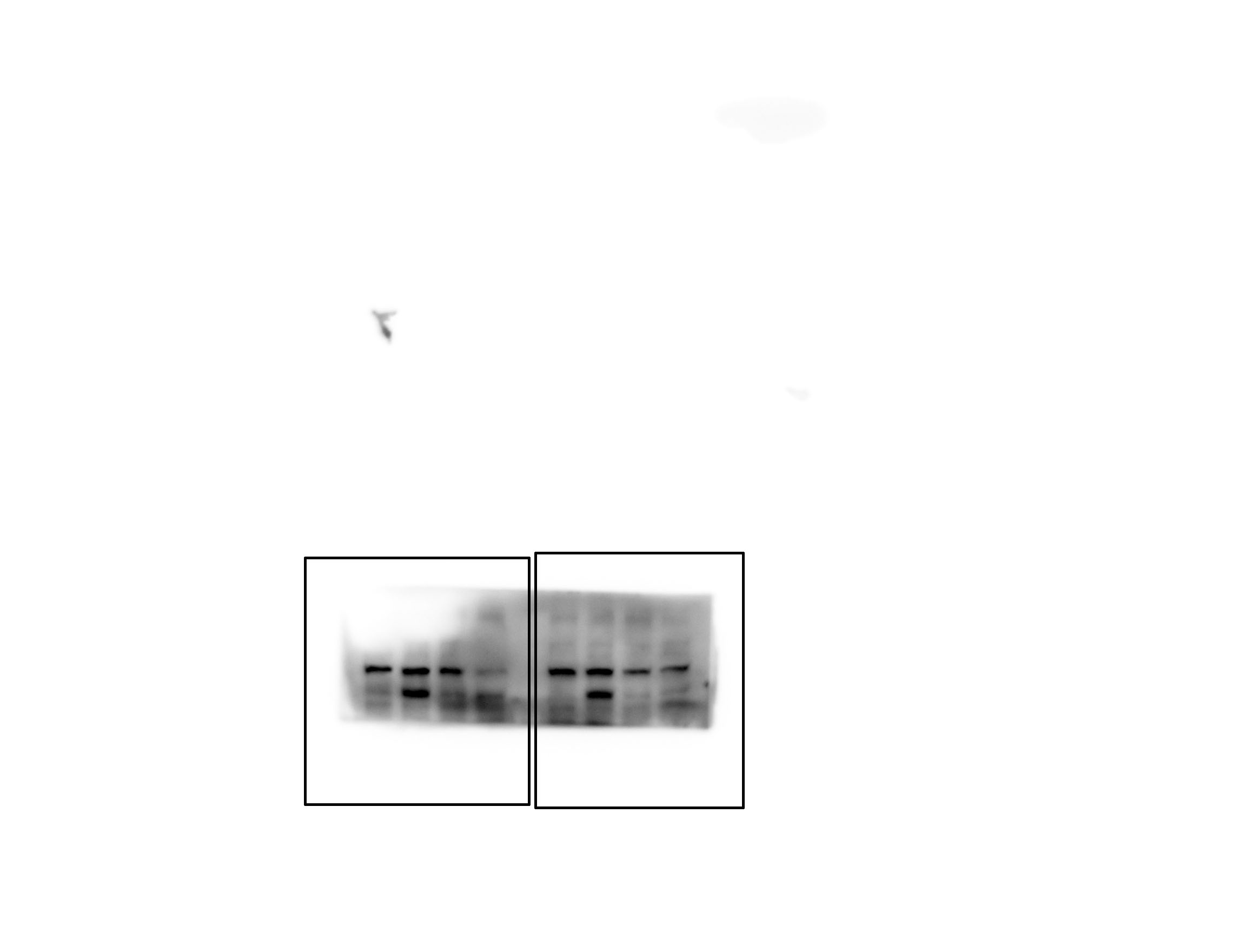

Supplement: Supplementary file 1 [file DataSheet1.ZIP › Original data/Supplementary figure 8-original data/P-IKK-DTT-BVA-1,2.jpg]
